# Comparative safety of tyrosine kinase inhibitors in the treatment of metastatic renal cell carcinoma: a systematic review and network meta-analysis

**DOI:** 10.3389/fphar.2023.1223929

**Published:** 2023-09-07

**Authors:** Kinga Krawczyk, Katarzyna Śladowska, Przemysław Holko, Paweł Kawalec

**Affiliations:** ^1^ Faculty of Health Sciences, Institute of Public Health, Jagiellonian University Medical College, Krakow, Poland; ^2^ Department of Nutrition and Drug Research, Faculty of Health Sciences, Institute of Public Health, Jagiellonian University Medical College, Krakow, Poland

**Keywords:** renal cell carcinoma, safety, tyrosine kinase inhibitors, meta-analysis, systematic review

## Abstract

**Objective:** This study aimed to compare the safety profile of tyrosine kinase inhibitors (TKIs) approved for use as monotherapy or combination therapy for the first-line treatment of adult patients with metastatic clear cell renal cell carcinoma (RCC).

**Methods:** A systematic review with frequentist network meta-analysis (NMA) was performed according to the Preferred Reporting Items for Systematic Reviews and Meta-Analyses guidelines. We included randomized controlled trials (RCTs) investigating the use of: cabozantinib, pazopanib, sorafenib, sunitinib, tivozanib, cabozantinib + nivolumab, lenvatinib + pembrolizumab, axitinib + avelumab, and axitinib + pembrolizumab in previously untreated adult patients with metastatic clear cell RCC. Eligible studies were identified by two reviewers in MEDLINE (via PubMed), EMBASE, and Cochrane Library. The risk of bias for RCTs was assessed using the Cochrane Collaboration tool. The P score was used to determine the treatment ranking. The mean probability of an event along with the relative measures of the NMA was considered with the treatment rankings.

**Results:** A total of 13 RCTs were included in the systematic review and NMA. Sorafenib and tivozanib used as monotherapy were the best treatment options. Sorafenib achieved the highest P score for treatment discontinuation due to adverse events (AEs), fatigue, nausea, vomiting of any grade, and hypertension of any grade or grade ≥3. Tivozanib achieved the highest P score for AEs, grade ≥3 AEs, dose modifications due to AEs, and grade ≥3 diarrhea. Sunitinib was the best treatment option in terms of diarrhea and dysphonia of any grade, while cabozantinib, pazopanib, and axitinib + pembrolizumab–in terms of grade ≥3 fatigue, nausea, and vomiting. TKIs used in combination were shown to have a poorer safety profile than those used as monotherapy. Lenvatinib + pembrolizumab was considered the worst option in terms of any AEs, grade ≥3 AEs, treatment discontinuation due to AEs, dose modifications due to AEs, fatigue of any grade, nausea, vomiting, and grade ≥3 nausea. Axitinib + avelumab was the worst treatment option in terms of dysphonia, grade ≥3 diarrhea, and hypertension, while cabozantinib + nivolumab was the worst option in terms of grade ≥3 vomiting. Interestingly, among the other safety endpoints, cabozantinib monotherapy had the lowest P score for diarrhea and hypertension of any grade.

**Conclusion:** The general safety profile, including common AEs, is better when TKIs are used as monotherapy vs. in combination with immunological agents. To confirm these findings, further research is needed, including large RCTs.

## 1 Introduction

Renal malignancies are relatively rare, with renal cell carcinoma (RCC) being the most common, accounting for approximately 90% of all cases (Hsieh et al., 2017). The incidence of kidney cancer peaks between the sixth and eighth decade of life and is estimated at 74,000 new cases annually in the United States (Siegel et al., 2019). There are several subtypes of RCC classified on the basis of microscopic examination of a tumor specimen. The most common subtypes include clear cell RCC (75%), papillary RCC (10%), and chromophobic RCC (5%) (Hsieh et al., 2017; Padala et al., 2020). Clear cell RCC is the most serious diagnosis, as this subtype is linked with the presence of distant metastases and the highest grade of histological malignancy at diagnosis (Hsieh et al., 2017; Protzel et al., 2012).

The prognosis of patients with RCC depends on the clinical stage of cancer. The 5-year survival rate is 80%–90% for patients with stage I cancer; 50%–70%, with stage II; 20%–30%, with stage III; and about 5%, with stage IV (Siegel et al., 2019; Padala et al., 2020). Most patients present with localized disease (stage I or II) that can be treated surgically; however, up to 20%–30% of patients who undergo surgical resection may relapse and develop metastases (Tyson and Chang, 2017). Moreover, about 25% of patients with RCC have locally advanced or metastatic disease at diagnosis, and in approximately 20%–40% of patients, localized primary tumors will metastasize (Osawa et al., 2019). Therefore, it is particularly important to choose an appropriate therapeutic option that would allow to improve survival and the quality of life of patients with advanced kidney cancer.

Treatment depends on the stage of cancer at diagnosis. For patients in early stages (I or II), the most common treatment options are surgical tumor excision and partial or complete nephrectomy. The standard therapeutic strategy in advanced kidney cancer has changed with the introduction of molecularly targeted drugs that selectively inhibit tumor growth without affecting the growth of other rapidly dividing cells. Targeted therapy for kidney cancer includes three groups of drugs: tyrosine kinase inhibitors (TKIs), mTOR serine-threonine kinase inhibitors, and anti–vascular endothelial growth factor (VEGF) monoclonal antibody (Hsieh et al., 2017). The new molecularly targeted drugs have vastly improved the prognosis of patients with advanced kidney cancer, with a significant increase in the median overall survival (Thomson et al., 2023).

In patients with RCC, changes in the von Hippel–Lindau gene, VHL, cause the activation of angiogenic factors such as an increase in VEGF levels. Thus, TKIs, which prevent cell division and growth of new blood vessels, seem to be the most effective therapeutic option (Thomson et al., 2023; Pal et al., 2012). The drugs precisely target the genetic mechanisms based on oncogenesis and proliferation of renal cancer cells (Roberto et al., 2021). According to the latest data from the National Cancer Institute, the following TKIs are currently approved for use by the Food and Drug Administration (FDA): sunitinib, sorafenib, pazopanib, tivozanib, lenvatinib, axitinib, and cabozantinib (cancer.gov). A network meta-analysis (NMA) showed no differences in the effectiveness of TKIs used as monotherapy (Manz et al., 2020). TKIs were reported to be highly effective in terms of improving the median progression-free survival and overall survival (Mihály et al., 2012; Motzer et al., 2006). The objective response rate ranged from 20% to 35% (Hutson et al., 2010; Wang et al., 2020). Studies conducted in recent years also provided the basis for approving TKI use in combination therapy for metastatic RCC. Hahn et al. (2019) reported that cabozantinib treatment, combination therapy with avelumab and axitinib, and combination therapy with pembrolizumab and axitinib have comparable efficacy and are the preferred treatment option for most patients with metastatic RCC (Hahn et al., 2019).

As each drug, especially anticancer drug, has a certain toxicity profile, it is often necessary to modify treatment to prevent the high rate of side effects and to control for side effects so that adequate therapy can be continued (Oh et al., 2014). However, to our knowledge, there have been no systematic reviews that would assess the safety profile of TKIs in a more comprehensive way by focusing on the risk of individual AEs. In addition, as new TKIs have been approved for use in the last few years, an update of the current knowledge is needed. We assumed that if individual TKIs have similar effectiveness, an in-depth assessment of the safety profile might help clinicians in decision-making on the best and safest therapy for individual patients.

To fill in the existing gaps in knowledge and evidence, we decided to compare the safety profile of TKIs used in adult patients with metastatic clear cell RCC. We conducted a systematic review with an NMA with the aim to perform a comprehensive safety assessment of selected TKIs approved for this indication.

## 2 Materials and methods

### 2.1 General principles

The systematic review was conducted according to the Preferred Reporting Items for Systematic Reviews and Meta-Analyses (PRISMA) guidelines (Hutton et al., 2015; Page et al., 2021) and guidelines for conducting and interpreting the NMA developed by the International Society for Pharmacoeconomics and Outcomes Research Task Force (Jansen et al., 2014) and Cipriani et al. (2013). The protocol of systematic review was registered in the International Prospective Register of Systematic Reviews (PROSPERO; registration number, CRD42022375275) (PROSPERO database, 2022).

### 2.2 Data sources and search

A comprehensive search of the three main databases: MEDLINE (via PubMed), EMBASE, and Cochrane Library, was conducted in November 2022. During the search, keywords related to the analyzed population and interventions were used, identified in medical subject heading (MeSH) terms or Emtree, combined with Boolean logical operators. The detailed search strategy is described in Supplementary Table S1. In addition, the trial registration database https://clinicaltrials.gov/ (the detailed search strategy is described in Supplementary Table S2), the reference lists of the most recent systematic reviews on TKI use in metastatic RCC, and the reference lists of the included studies were hand searched. Only articles written in English were included.

### 2.3 Inclusion criteria and trial section process

Full-text publications of prospective randomized controlled trials (RCTs) published in English, conducted in a group of adult patients with a clinical diagnosis of metastatic clear cell RCC, treated with TKIs as monotherapy or combination therapy, were included. The following TKI-based therapies approved by the European Medicines Agency (EMA) and/or the Food and Drug Administration (FDA) included: tivozanib, sunitinib, sorafenib, pazopanib, cabozantinib + nivolumab, lenvatinib + pembrolizumab, axitinib + avelumab, and axitinib + pembrolizumab compared with one another, with placebo, or with other therapy registered by the EMA or FDA for the first-line treatment of RCC. The safety outcomes of interest were as follows: 1) AEs (all grades and grade ≥3); 2) treatment discontinuation due to AEs; 3) dose modification due to AEs; and 4) individual AEs (all grades and grade ≥3) that are most commonly reported in the summary of products characteristics (SmPC Fortivda^®^, SmPC Sutent^®^, SmPC Votrient^®^, SmPC Inlyta^®^, SmPC Cabometyx^®^). These individual AEs included fatigue, diarrhea, nausea, vomiting, hypertension, and dysphonia. If no appropriate data were available in a full-text publication, information from clinical trial registries was allowed. The most recent available data were considered. Detailed inclusion and exclusion criteria for the systematic review and meta-analysis are described in Table 1 and Supplementary Table S3.

**TABLE 1:** Detailed inclusion and exclusion criteria for systematic review and meta-analysis.

	Inclusion criteria	Exclusion criteria
Population	Adult patients (or majority of patients) with metastatic clear cell RCC (or with clear cell component) not previously treated systemically (trials with ≥70% of patients previously untreated were eligible; the local treatment such surgery or radiotherapy were allowed)	Pediatric patients with RCC, patients previously treated, trials with no information about line of therapy, trials with patients with other than clear cell RCC
Intervention and comparators	Registered TKIs in monotherapy or in combination therapy (details about dosing provided in Supplementary Table S3): tivozanib, sunitinib, sorafenib, pazopanib, cabozantinib + nivolumab, lenvatinib + pembrolizumab, axitinib + avelumab, axitinib + pembrolizumab compared to each other or with placebo or with other therapy registered by EMA or FDA for first line treatment of RCC	Interventions not of interest (e.g., not approved for metastatic RCC); trials without direct comparison of safety of any of the mentioned interventions to any other included TKI or placebo or other therapy registered by EMA or FDA for first line treatment of RCC
Outcomes	Adverse events, grade ≥3 adverse events, discontinuation because adverse events, dose modification due to adverse events, individual adverse events (all grades and grade ≥3): fatigue, diarrhea, nausea, vomiting, hypertension, dysphonia	Trials or additional articles for included trials not reported defined outcomes
Study types	Randomized controlled trials	Non-randomized controlled, trials, observational studies, case reports, reviews, additional analysis of included trials, additional references for included trials without safety data or without newer safety data than reported in main publication, cross-sectional studies
Treatment period	Duration of treatment: until disease progression or as long as clinical benefit is observed or until unacceptable toxicity occurs	Trials in which the length of the treatment period was predetermined/restricted, regardless of progression, toxicity, or treatment benefit
Publication type	Full text articles, data from clinical trials registers were allowed to use	Abstracts, posters, editorials, letters
Language	English	Languages other than English

EMA, European Medicines Agency; FDA, Food and Drug Administration; TKIs, tyrosine kinase inhibitors; RCC, renal cell carcinoma.

The screening and selection of studies were carried out in accordance with the PRISMA guidelines (Hutton et al., 2015; Page et al., 2021) by two independent reviewers (KŚ, KK). First, the titles and abstracts of studies identified during the search were assessed, and a list of studies that initially met the inclusion criteria was prepared. Then, the full texts of the remaining articles were examined to determine whether they contained relevant information, considering all the inclusion and exclusion criteria for the analysis. The degree of compatibility between the reviewers was high (estimated at 97.5%). Conflicts in study selection at this stage were resolved by consensus and consultation with a third reviewer (PK), referring to the original article. At the end of the selection process, the final list of included trials was prepared.

### 2.4 Data extraction and quality assessment

Two independent reviewers (KŚ, KK) extracted data from included trials, using a predefined data extraction form. The following information was extracted to assess the homogeneity of trials: design (methodology), treatment regimens, size of the study arms, duration of treatment/exposure to the drug, and detailed patient characteristics including the stage and histological type of RCC, age, sex, performance status, previous surgery and/or radiation therapy, and prognosis according to Memorial Sloan-Kettering Cancer Center (MSKCC) criteria. The Cochrane risk-of-bias tool 2 (RoB2) for randomized trials (Higgins et al., 2019) was used to assess the bias of eligible RCTs. This tool allows an evaluation of the following domains: randomization process, deviations from intended intervention, missing data outcome, measurement of the outcome and selection of the reported results. The domain-based evaluation allows the assignment of the following ratings to each domain: low risk of bias (“+”), high risk of bias (“–”), or unclear risk of bias (“?”). The robvis tool (https://www.riskofbias.info/welcome/robvis-visualization-tool) was used to graphically present the results of the risk-of-bias assessment for individual trials.

### 2.5 Data analysis and synthesis

The NMA was conducted using the netmeta R software package (Rücker, 2012), which incorporates the graph-theoretic method of an NMA (vertices, treatments; edges, randomized comparisons) and provides a point estimate from the network along with 95% confidence intervals (CIs). This frequentist method is an alternative to a standard NMA conducted within the Bayesian framework (Neupane et al., 2014). In the NMA, we used consistency and random effects models with adjustment for multi-arm studies. All eligible treatments and their regimens with different doses or dosing intervals from the identified studies were included in the network, and each treatment in each dose regimen constituted one node (vertex in a graph).

All comparisons evaluated in the trials, including suboptimal and experimental dose regimens and treatments not assessed in the systematic review, were included in the NMA and presented in Supplementary Material. However, only the treatments of interest in their licensed dose regimens were presented (Supplementary Table S3). These treatments included: oral tivozanib (1.5 mg once daily for 3 weeks, followed by 1 week off), oral sunitinib (50 mg once daily for 4 weeks, followed by 2 weeks off), oral sorafenib (400 mg twice daily), oral pazopanib (800 mg once daily), oral cabozantinib (40 mg once daily) + intravenous nivolumab (240 mg once every 2 weeks), oral lenvatinib (20 mg once daily) + intravenous pembrolizumab (200 mg once every 3 weeks), oral axitinib (5 mg twice daily) + intravenous avelumab (10 mg per kilogram of bod weight every 2 weeks), and oral axitinib (5 mg twice daily) + intravenous pembrolizumab (200 mg once every 3 weeks).

The networks were created for each outcome with a similar definition in all trials. The heterogeneity of evidence was assessed using the Cochran’s Q test, I^2^ statistic, and tau (i.e., the square-root of between-study variance). The consistency of the network was assessed using a design-based decomposition of Cochran’s Q, the splitting approach, and comparison with direct evidence (Freeman et al., 2019). The funnel plot for “small-study effects” was used to assess publication bias.

The P score, a frequentist equivalent of the surface under the cumulative ranking curve, was used to determine the treatment ranking. A higher P score indicates better treatment safety (i.e., lower risk of AEs) (Rücker and Schwarzer, 2015). The treatment ranking alone should be interpreted with caution, because it provides information only about the probability that a treatment is the best while not directly incorporating the effect size of the difference between treatments. The mean probability of an event along with the relative measures of the NMA should be taking into account with the treatment rankings (Manz et al., 2020; Bhatnagar et al., 2014). The mean probability of an event for each treatment was calculated using the odds ratio from the NMA and the mean probability for sunitinib. The latter was obtained from the meta-analysis of the sunitinib arm from all trials included in the NMA, using the random effects model based on the Freeman-Tukey (double arcsine) transformed proportion.

## 3 Results

### 3.1 Search results and included studies

**FIGURE 1:**
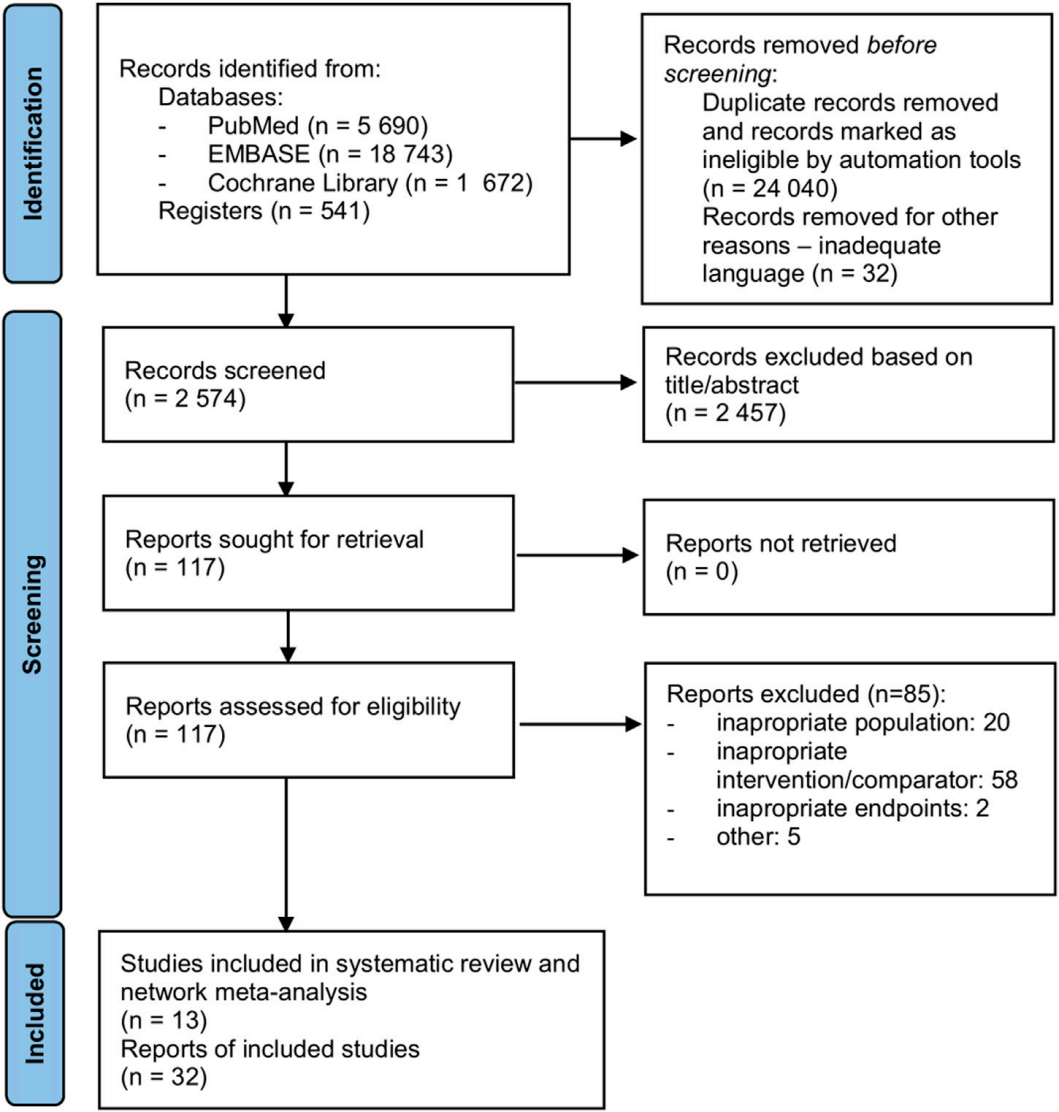
Search flow diagram.

During the database search, a total of 2,574 possibly relevant references were screened, of which 2,372 were excluded after screening the titles and abstracts (Figure 1). After careful consideration of 117 articles assessed in the full-text review, 85 were excluded (Supplementary Table S4). Finally, 13 trials, described in 32 references (7,125 patients randomized), were included in the review and meta-analysis. The methodology of included trials is characterized in Table 2, and the baseline characteristics of patients are provided in Supplementary Table S5.

**TABLE 2:** Methodology of trials included in systematic review and network meta-analysis.

Study	Methodology	Comparison and the number of randomized patients	Median duration of treatment (range)
NCT00098657, NCT00083889 Motzer et al. (2007), Motzer et al. (2009), NCT00083889, (3889), NCT00098657, (8657)	RTC, partially-blinded (blinded only for primary efficacy endpoint analysis), phase III, multicenter, parallel groups	Sunitinib orally at a dose of 50 mg once daily for 4 weeks, followed by 2 weeks without treatment (*N* = 375) vs.	11.0 months (<1–41 months) in the sunitinib group and 4.0 months (range <1–40 months) in the interferon alpha-2a group
		Interferon alpha-2a subcutaneously at a dose of 9 MU thrice a week (N = 375)	
NCT00117637 Escudier et al. (2009), NCT00117637 (1176)	RCT, open, phase II, multicenter, parallel groups	Sorafenib orally at a dose of 400 mg twice daily (*N* = 97) vs.	6.0 months (0.2–13.8) in the sorafenib and 5.5 months (0.4–7.5) in inferferon alpha-2a group
		Interferon alfa-2a subcutaneously at a dose of 9 MU thrice a week (N = 92)	
TIVO Motzer et al. (2013a), NCT01030783, (1030)	RCT, open (only independent radiology review blinded), phase III, multicenter, parallel groups	Sorafenib orally at a dose of 400 mg twice daily (*N* = 257) vs.	12.0 months in tivozanib and 9.5 months in sorafenib group
		Tivozanib orally at a dose of 1.5 mg once daily every day for 3 weeks followed by 1 week off (*N* = 260)	
Alliance A031203 CABOSUN Choueiri et al. (2017), Choueiri et al. (2018), NCT01835158 (1835)	RCT, open, phase II, multicenter, parallel groups	Sunitinib orally at a dose of 50 mg for 4 weeks, followed by 2 weeks without treatment (*N* = 78) vs.	6.5 months (IQR 2.8–16.5) in the cabozantinib and 3.1 months (IQR 2.0–8.2) in sunitinib group
		Cabozantinib orally at a dose of 60 mg once daily (*N* = 79)	
COMPARZ Motzer et al. (2013b), NCT00720941 (2094)	RCT, open, phase III, multicenter, parallel group	Pazopanib orally at a dose of 800 mg once daily (*N* = 557) vs.	8.0 months (0–40.0) in the pazopanib and 7.6 months (0–38.0) in sunitinib group
		Sunitinib orally at a dose of 50 mg once daily for 4 weeks, followed by 2 weeks without treatment (*N* = 553)	
SWITCH^a^ Eichelberg et al. (2015), NCT00732914 (2914)	RCT, open, phase III, multicenter, cross-over (but results for first line-treatment provided)	Sorafenib orally at a dose of 400 mg twice daily (*N* = 182) vs.	During first-line treatment: 37.5 weeks (SD = 37.4) in sorafenib group and 43.9 weeks (SD = 44.3) in sunitinib group
		Sunitinib orally at a dose of 50 mg once daily for 4 weeks, followed by 2 weeks without treatment (*N* = 183)	
SWITCH II^a^ Retz et al. (2019), NCT01613846 (1613)	RCT, open, phase III, multicenter, cross-over (but results for first line-treatment provided)	Sorafenib orally at a dose of 400 mg twice daily (*N* = 189) vs.	During first-line treatment: 3.9 months (0.0–42.2) for sorafenib and 5.7 months (0.3–43.3) for a pazopanib group
		Pazopanib orally at a dose of 800 mg once daily (*N* = 188)	
CROSS-J -RCC^a^ Tomita et al. (2017), Tomita et al. (2020), NCT01481870 (1481)	RCT, open, phase III, multicenter, cross-over (but results for first line-treatment provided)	Sunitinib orally at a dose of 50 mg once daily for 4 weeks, followed by 2 weeks without treatment (*N* = 60) vs.	During first-line treatment: 6.7 months (0.1–45.3) for sunitinib and 6.1 months (0.3–46.1) for sorafenib group
		Sorafenib orally at a dose of 400 mg twice daily (*N* = 64)	
TemPa Tannir et al. (2020)	RCT, open (only response to treatment assessed by blind investigator), phase II, parallel group	Temsirolimus intravenously at a dose of 25 mg twice a week (*N* = 35) vs.	Not reported, but median PFS for temsirolimus group was 2.7 months and 5.2 months for pazopanib group
		Pazopanib orally at a dose of 800 mg one daily (*N* = 34)	
CheckMate 9ER Motzer et al. (2022a), Choueiri et al. (2021), NCT03141177, (1411)	RCT, open (blinded only for primary efficacy endpoint analysis), phase III, multicenter, parallel group	Nivolumab intravenously at a dose of 240 mg once every 2 weeks + cabozantinib orally at a dose of 40 mg once daily (*N* = 323) vs.	14.3 months (0.2–27.3) for nivolumab + cabozantinib and 9.2 months (0.8–27.6) for sunitinib
		Sunitinib orally at a dose of 50 mg once daily for 4 weeks, followed by 2 weeks without treatment (*N* = 328)	Total median observation time of 18.1 months
KEYNOTE-426 Rini et al. (2019), Powles et al. (2020), NCT02853331 (2853)	RCT, open (blinded only for primary efficacy endpoint analysis), phase III, multicenter, parallel group	Pembrolizumab intravenously at a dose of 200 mg once every 3 weeks + axitinib orally at a dose of 5 mg twice daily (*N* = 432) vs.	The median duration of any treatment was 10.4 months (0.03–21.2) in the pembrolizumab + axitinib group and 7.8 months (0.07–20.5) in the sunitinib group
		Sunitinib orally at a dose of 50 mg once daily for 4 weeks, followed by 2 weeks without treatment (*N* = 429)	Total median observation time of 30.6 (23.4–38.4) months
CLEAR Motzer et al. (2021), NCT02811861 (2811)	RCT, open, phase III, multicenter, parallel group	Lenvatinib orally at a dose of 20 mg once daily + pembrolizumab intravenously at a dose of 200 mg once every 3 weeks (*N* = 355) vs.	17.0 months (0.1–39.1) in the lenvatinib + pembrolizumab, 11.0 months (0.1–40.0) in the lenvatinib + everolimus group, and 7.8 months (0.1–37.0) in the sunitinib group. Median observation period for the total survival of 26.6 months
		Lenvatinib orally at a dose of 18 mg once daily + everolimus orally at a dose of 5 mg once daily (*N* = 357) vs.	
		Sunitinib orally at a dose of 50 mg once daily for 4 weeks, followed by 2 weeks without treatment (*N* = 357)	
JAVELIN Renal 101 Rini et al. (2022), Motzer et al. (2019), NCT02684006 (2684)	RCT, open (blinded only for primary efficacy endpoint analysis), phase III, multicenter, parallel group	Avelumab intravenously at a dose of 10 mg per kilogram of body weight every 2 weeks + axitinib orally at a dose of 5 mg twice daily (*N* = 442) vs.	8.6 months (0.5–25.3) in patients who received avelumab, 9.0 months (0.02–24.9) in patients who received axitinib, and 7.3 months (0.2–23.0) in the sunitinib group
		Sunitinib orally at a dose of 50 mg once daily for 4 weeks, followed by 2 weeks without treatment (*N* = 444)	During the first indirect analysis, the minimum observation period was 6 months

^a^ After disease progression, treatment was changed to an alternative drug. Only first-line data were used in the meta-analysis; RCT, randomized clinical trial; IQR, interquartile range; SD, standard deviation.

#### 3.1.1 Homogeneity of included trials and risk-of-bias assessment

**FIGURE 2:**
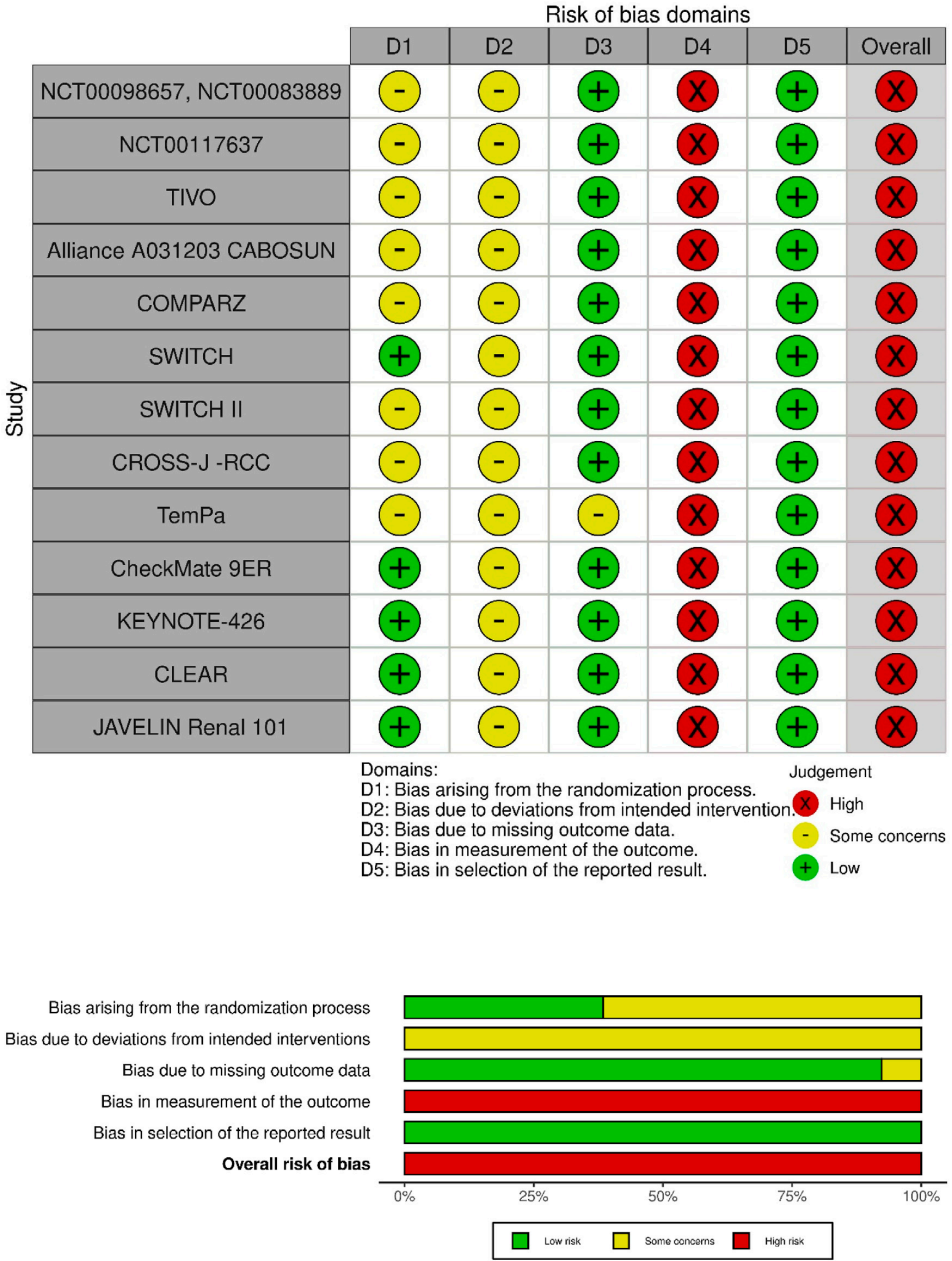
Risk-of-bias 2 assessment.

Most studies were multicenter randomized phase III trials, except phase II NCT00117637 (Escudier et al., 2009; NCT00117637, 1176), the Alliance A031203 CABOSUN (Choueiri et al., 2017; Choueiri et al., 2018; NCT01835158, 1835), and TemPa (Tannir et al., 2020). Ten trials had a parallel design, while the remaining three trials [SWITCH (Eichelberg et al., 2015; NCT00732914, 2914), SWITCH II (Retz et al., 2019; NCT01613846, 1613) and CROSS-J–RCC (Tomita et al., 2017; Tomita et al., 2020; NCT01481870, 1481)] had a sequential cross-over design, that is, after disease progression during the first-line randomized treatment, patients received the therapy used in the other group. In line with the inclusion criteria for systematic review and meta-analysis, only data for the first-line setting were used in the case of studies with a sequential cross-over design. All included trials were open label; however, in some studies [NCT00098657/NCT00083889 (Motzer et al., 2007; Motzer et al., 2009; NCT00098657, 8657; NCT00083889, 3889), TIVO (Motzer et al., 2013a; NCT01030783, 1030), TemPa (Tannir et al., 2020), CheckMate 9ER (Motzer et al., 2022a; Choueiri et al., 2021; NCT03141177, 1411), KEYNOTE-426 (Rini et al., 2019; Powles et al., 2020; NCT02853331, 2853), and JAVELIN Renal 101 (Rini et al., 2022; Motzer et al., 2019; NCT02684006, 2684)], only the analysis of the primary endpoint (survival outcomes such as progression-free survival) was assessed by a blinded investigator or a blinded radiology review. This means that in all studies, both patients and physicians/medical staff involved in the safety evaluation were unblinded, resulting in a high risk-of-bias assessment in the Cochrane risk-of-bias tool 2 (Figure 2).

According to the inclusion criteria and baseline characteristics, participants in all studies were patients with advanced or metastatic (most patients) RCC with clear cell histology or with a clear cell component. The most common metastatic sites were the lungs, lymph nodes, and bones. In most trials, all patients were systemic therapy naïve, except the TIVO trial, where >70% of patients were systemic therapy naïve. The median age of patients in included trials ranged from 59 to 68 years, and patients had generally good performance status (Eastern Cooperative Oncology Group [ECOG] score of 0–1). Most patients had favorable or intermediate prognosis according to MSKCC criteria, except the TemPa trial, where about 70% of participants had poor prognosis (Tannir et al., 2020). The most common TKI therapy was sunitinib monotherapy. In four trials [CheckMate 9ER (Motzer et al., 2022a; Choueiri et al., 2021; NCT03141177, 1411), KEYNOTE-426 (Rini et al., 2019; Powles et al. 2020; NCT02853331, 2853), CLEAR (Motzer et al., 2021; NCT02811861, 2811) and JAVELIN Renal 101 (Rini et al., 2022; Motzer et al., 2019; NCT02684006, 2684)], TKIs were used in combination with anti-PD-1 monoclonal antibodies (nivolumab, pembrolizumab, or avelumab) (Supplementary Table S5). The therapy was continued until disease progession or unacceptable toxicity occurred, which was in line with the recommendations in the summary of product characteristics.

### 3.2 NMA results

Thirteen trials had sufficient homogeneity to be included in the NMA (Supplementary Figure S1; Supplementary Tables S5, S6). Not all predefined endpoints were reported in each trial, and for grade ≥3 dysphonia, it was impossible to conduct an NMA. The final number of trials for each endpoint is presented in Supplementary Table S7. The input data for NMA for the overall safety profile were presented in the Supplementary Table S8; for the detailed safety profile results - selected adverse events in all grades in Supplementary Table S9 and for selected grade ≥3 adverse events in Supplementary Table S10.

### 3.3 Rakings of TKIs

The P score–based ranking of TKIs (and interventions used as comparators in included trials) is presented in Table 3 and Supplementary Table S11 (all therapies from included trials).

**TABLE 3:** Overall ranking of TKIs used as monotherapy and as combination therapy based on the P score for assessed endpoints. Numbers in the parenthesis indicate the therapy position in the ranking.

Intervention	Adverse events	Grade ≥3 adverse events	Discontinuation due to adverse events	Dose change due to adverse events	Fatigue	Diarrhea	Nausea	Vomiting	Hypertension	Dysphonia	Grade ≥3 fatigue	Grade ≥3 diarrhea	Grade ≥3 nausea	Grade ≥3 vomiting	Grade ≥3 hypertension
TIV	0.871 (1)	0.901 (1)	0.662 (4)	0.998 (1)	0.631 (3)	0.757 (2)	0.435 (6)	—	0.494 (5)	—	0.496 (5)	0.801 (1)	0.548 (4)	—	0.463 (4)
SOR	0.606 (4)	0.705 (2)	0.729 (1)	0.647 (3)	0.862 (1)	0.397 (6)	0.844 (1)	0.929 (1)	0.847 (1)	—	0.729 (2)	0.254 (8)	0.569 (3)	0.687 (2)	0.823 (1)
AXI + AVE	0.422 (6)	0.551 (3)	—	0.446 (5)	0.287 (8)	0.338 (7)	0.545 (3)	0.514 (4)	0.171 (7)	0.153 (6)	0.341 (8)	0.183 (9)	0.523 (5)	0.609 (4)	0.231 (9)
SUN	0.527 (5)	0.534 (4)	0.641 (5)	0.412 (6)	0.371 (6)	0.828 (1)	0.317 (8)	0.416 (5)	0.622 (4)	1.000 (1)	0.277 (9)	0.766 (2)	0.484 (7)	0.425 (6)	0.601 (3)
AXI + PEM2	0.817 (3)	0.528 (5)	0.184 (7)	0.727 (2)	0.332 (7)	0.520 (3)	0.511 (5)	0.649 (2)	0.647 (3)	0.346 (4)	0.720 (3)	0.305 (7)	0.492 (6)	0.765 (1)	0.450 (5)
PAZ	0.295 (7)	0.460 (6)	0.726 (2)	0.521 (4)	0.709 (2)	0.500 (4)	0.394 (7)	0.346 (7)	0.329 (6)	—	0.606 (4)	0.656 (4)	0.656 (1)	0.574 (5)	0.388 (6)
CAB	0.857 (2)	0.427 (7)	0.667 (3)	0.249 (7)	0.540 (4)	0.207 (9)	0.580 (2)	0.392 (6)	0.057 (9)	0.383 (3)	0.764 (1)	0.734 (3)	0.595 (2)	0.628 (3)	0.320 (7)
CAB + NIV	0.273 (8)	0.289 (8)	0.533 (6)	—	0.505 (5)	0.275 (8)	0.524 (4)	0.627 (3)	0.704 (2)	0.691 (2)	0.464 (6)	0.449 (5)	0.356 (8)	0.073 (8)	0.603 (2)
LEN + PEM	0.167 (9)	0.080 (9)	0.103 (8)	0.026 (8)	0.199 (9)	0.435 (5)	0.245 (9)	0.156 (8)	0.165 (8)	0.321 (5)	0.342 (7)	0.352 (6)	0.195 (9)	0.171 (7)	0.234 (8)

AXI + AVE, axitinib + avelumab; AXI + PEM2–axitinib + pembrolizumab; CAB, cabozantinib; CAB + NIV, cabozantinib + nivolumab; LEN + PEM, lenvatinib + pembrolizumab; PAZ, pazopanib; SOR, sorafenib; SUN, sunitinib; TIV, tivozanib.

The results indicated that individual drugs and combination therapies rank differently depending on the safety endpoint. The TKIs sorafenib and tivozanib were shown to be the best treatment options: sorafenib had the highest P score in terms of treatment discontinuation due to AEs, fatigue, nausea, vomiting, and hypertension of any grade, and grade ≥3 hypertension. Tivozanib had the highest P score in terms of any AEs, grade ≥3 AEs, dose modifications due to AEs, and grade ≥3 diarrhea. The best treatment option in terms of diarrhea and dysphonia was sunitinib, while cabozantinib, pazopanib, and axitinib + pembrolizumab were ranked as the best options in terms of fatigue, nausea, and vomiting (all grade ≥3).

Generally, TKIs in combination with other drugs were found to have a poorer safety profile than TKI monotherapies. Lenvatinib + pembrolizumab was ranked as the worst option in terms of any AEs, grade ≥3 AEs, treatment discontinuation due to AEs, dose modifications due to AEs, fatigue, nausea, and vomiting of any grade, and grade ≥3 nausea. Axitinib + avelumab was the worst option in terms of dysphonia of any grade, grade ≥3 diarrhea, and grade ≥3 hypertension, while cabozantinib + nivolumab was the worst option in terms of grade ≥3 vomiting. Interesingly, considering the remaining safety endpoints, cabozantinib monotherapy showed the lowest P score for diarrhea and hypertension of any grade.

#### 3.3.1 General safety profile

The general safety profile was assessed in terms of any AEs, grade ≥3 AEs, treatment discontinuation due to AEs, and dose modifications due to AEs. Most trials reported AEs as treatment-emergent AEs irrespective of their relation to the therapy used. Two trials [NCT00098657/NCT00083889 (Motzer et al., 2007; Motzer et al., 2009; NCT00098657, 8657; NCT00083889, 3889 and NCT00117637 (Escudier et al., 2009; NCT00117637, 1176)] reported individual AEs only as treatment-related AEs, so it was impossible to use these results in the NMA. All included trials generally used a similar definition of treatment discontinuation due to AEs and recorded AEs using the Common Terminology Criteria for Adverse Events, which allowed us to conduct a credible NMA (Supplementary Table S6).

There were no differences between TKIs (monotherapy and combination therapy) in the risk of any AEs, except a reduced risk of AEs for: 1) tivozanib vs. sorafenib (*p* = 0.006), sunitinib (*p* = 0.048), pazopanib (*p* = 0.009), and lenvatinib + pembrolizumab (*p* = 0.018); and 2) axitinib + pembrolizumab vs. lenvatinib + pembrolizumab (*p* = 0.032) (Table 4; Supplementary Figure S2; Supplementary Table S12). The adjusted mean risk of any AEs was generally similar for the treatments, with the highest risk for lenvatinib + pembrolizumab (99.8%; 95% CI: 98.4%, 100.0%), and the lowest risk for tivozanib (95.9%; 95% CI: 84.4%, 98.6%) and cabozantinib (93.8%; 95% CI: 43.4%, 99.5%) (Table 5).

**TABLE 4:** Results of a comparative analysis of TKIs used as monotherapy and as combination therapy (at approved doses) in terms of: A–adverse events; B–grade ≥3 adverse events, C–treatment discontinuation due to adverse events, D–dose modifications due to adverse events.

A								
TIV	—	—	0.31 (0.14–0.71)	—	—	—	—	—
1.55 (0.06–42.70)	CAB	—	—	0.15 (0.01–2.93)	—	—	—	—
0.81 (0.09–6.91)	0.52 (0.02–15.21)	AXI + PEM2	—	0.29 (0.06–1.38)	—	—	—	—
0.31 (0.14–0.71)	0.20 (0.01–5.05)	0.39 (0.05–2.83)	SOR	0.80 (0.21–3.03)	—	0.25 (0.03–2.22)	—	—
0.23 (0.05–0.99)	0.15 (0.01–2.93)	0.29 (0.06–1.38)	0.73 (0.22–2.44)	SUN	0.67 (0.11–4.05)	0.49 (0.09–2.70)	0.33 (0.03–3.20)	0.19 (0.02–1.64)
0.16 (0.02–1.56)	0.10 (0.00–3.25)	0.19 (0.02–2.09)	0.49 (0.06–4.27)	0.67 (0.11–4.05)	AXI + AVE	—	—	—
0.10 (0.02–0.56)	0.06 (0.00–1.72)	0.12 (0.01–1.02)	0.31 (0.07–1.46)	0.43 (0.10–1.76)	0.63 (0.06–6.25)	PAZ	—	—
0.08 (0.01–1.13)	0.05 (0.00–2.09)	0.09 (0.01–1.50)	0.24 (0.02–3.16)	0.33 (0.03–3.20)	0.49 (0.03–8.88)	0.78 (0.05–11.27)	CAB + NIV	—
0.04 (0.00–0.59)	0.03 (0.00–1.12)	0.05 (0.00–0.78)	0.14 (0.01–1.64)	0.19 (0.02–1.64)	0.28 (0.02–4.67)	0.45 (0.03–5.89)	0.58 (0.03–13.14)	LEN + PEM

B								
TIV	0.69 (0.48–1.00)	—	—	—	—	—	—	—
0.69 (0.48–1.00)	SOR	—	0.84 (0.56–1.26)	—	0.81 (0.53–1.24)	—	—	—
0.60 (0.34–1.05)	0.86 (0.56–1.32)	AXI + AVE	0.98 (0.73–1.32)	—	—	—	—	—
0.59 (0.36–0.95)	0.85 (0.62–1.16)	0.98 (0.73–1.32)	SUN	1.00 (0.74–1.34)	0.95 (0.73–1.24)	0.89 (0.45–1.75)	0.79 (0.56–1.12)	0.54 (0.38–0.78)
0.59 (0.33–1.03)	0.85 (0.55–1.30)	0.98 (0.65–1.49)	1.00 (0.74–1.34)	AXI + PEM2	—	—	—	—
0.56 (0.34–0.91)	0.81 (0.59–1.11)	0.94 (0.64–1.37)	0.95 (0.75–1.21)	0.95 (0.65–1.40)	PAZ	—	—	—
0.52 (0.23–1.20)	0.75 (0.36–1.59)	0.87 (0.42–1.83)	0.89 (0.45–1.75)	0.89 (0.42–1.86)	0.93 (0.45–1.92)	CAB	—	—
0.46 (0.26–0.84)	0.67 (0.42–1.07)	0.78 (0.49–1.22)	0.79 (0.56–1.12)	0.79 (0.50–1.25)	0.83 (0.54–1.27)	0.89 (0.41–1.91)	CAB + NIV	—
0.32 (0.17–0.58)	0.46 (0.29–0.74)	0.53 (0.34–0.85)	0.54 (0.38–0.78)	0.54 (0.34–0.87)	0.57 (0.37–0.88)	0.61 (0.28–1.32)	0.69 (0.42–1.14)	LEN + PEM

C							
SOR	1.58 (0.55–4.52)	—	0.95 (0.30–3.03)	0.64 (0.28–1.45)	—	—	—
1.01 (0.46–2.25)	PAZ	—	—	1.25 (0.47–3.37)	—	—	—
0.95 (0.24–3.82)	0.94 (0.22–4.02)	CAB	—	0.90 (0.26–3.08)	—	—	—
0.95 (0.30–3.03)	0.94 (0.23–3.84)	1.00 (0.16–6.12)	TIV	—	—	—	—
0.86 (0.45–1.65)	0.85 (0.39–1.85)	0.90 (0.26–3.08)	0.90 (0.24–3.41)	SUN	0.83 (0.30–2.31)	0.37 (0.13–1.00)	0.28 (0.10–0.78)
0.71 (0.21–2.40)	0.70 (0.19–2.55)	0.75 (0.15–3.71)	0.75 (0.14–4.02)	0.83 (0.30–2.31)	CAN + NIV	—	—
0.31 (0.09–1.05)	0.31 (0.09–1.11)	0.33 (0.07–1.62)	0.33 (0.06–1.75)	0.37 (0.13–1.00)	0.44 (0.11–1.87)	AXI + PEM2	—
0.24 (0.07–0.82)	0.24 (0.07–0.87)	0.26 (0.05–1.26)	0.26 (0.05–1.37)	0.28 (0.10–0.78)	0.34 (0.08–1.46)	0.77 (0.19–3.24)	LEN + PEM

D							
TIV	—	0.22 (0.11–0.42)	—	—	—	—	—
0.26 (0.10–0.70)	AXI + PEM2	—	—	—	0.59 (0.33–1.05)	—	—
0.22 (0.11–0.42)	0.83 (0.40–1.74)	SOR	0.69 (0.36–1.32)	—	0.99 (0.52–1.90)	—	—
0.18 (0.08–0.41)	0.69 (0.33–1.43)	0.83 (0.50–1.35)	PAZ	—	0.76 (0.44–1.30)	—	—
0.16 (0.06–0.42)	0.60 (0.27–1.34)	0.72 (0.35–1.48)	0.87 (0.43–1.79)	AXI + AVE	0.98 (0.56–1.71)	—	—
0.16 (0.07–0.34)	0.59 (0.33–1.05)	0.71 (0.45–1.12)	0.86 (0.55–1.35)	0.98 (0.56–1.71)	SUN	0.62 (0.27–1.41)	0.28 (0.15–0.50)
0.10 (0.03–0.30)	0.37 (0.13–1.00)	0.44 (0.17–1.12)	0.53 (0.21–1.36)	0.61 (0.23–1.64)	0.62 (0.27–1.41)	CAB	—
0.04 (0.02–0.12)	0.16 (0.07–0.38)	0.20 (0.09–0.42)	0.24 (0.11–0.50)	0.27 (0.12–0.61)	0.28 (0.15–0.50)	0.45 (0.16–1.23)	LEN + PEM

Data presented as ORs with 95% CIs. CAB, cabozantinib; PAZ, pazopanib; SOR, sorafenib; SUN, sunitinib; TIV, tivozanib; CAB + NIV, cabozantinib + nivolumab; LEN + PEM, lenvatinib + pembrolizumab; AXI + AVE, axitinib + avelumab; AXI + PEM2, axitnib + pembrolizumab. The results of direct comparisons are presented above the abbreviations of TKIs. On a gray background there is a symbol of appropriate intervention and on a white background statistically significant results are bolded.

**TABLE 5:** Mean probability of adverse events with 95% CIs in the brackets.

Intervention	Adverse events	Grade ≥3 adverse events	Discontinuation due to adverse events	Dose change due to adverse events	Fatigue	Diarrhea	Nausea	Vomiting	Hypertension	Dysphonia	Grade ≥3 fatigue	Grade ≥3 diarrhea	Grade ≥3 nausea	Grade ≥3 vomiting	Grade ≥3 hypertension
TIV	95.9% (84.4%–98.6%)	59.6% (47.7%–67.8%)	20.8% (6.5%–44.3%)	10.3% (5.0%–16.1%)	40.0% (27.9%–44.4%)	46.8% (29.4%–60.2%)	32.9% (17.7%–47.9%)	n/a	43.7% (30.6%–54.2%)	n/a	5.9% (1.3%–13.9%)	3.5% (1.1%–7.5%)	1.1% (0.0%–23.6%)	n/a	18.5% (7.9%–35.0%)
SOR	98.7% (95.7%–99.4%)	68.1% (60.9%–72.0%)	21.6% (12.5%–29.8%)	34.4% (25.0%–38.6%)	34.6% (28.3%–33.1%)	59.2% (48.4%–64.6%)	22.4% (15.8%–26.5%)	2.9% (0.6%–10.8%)	33.6% (26.2%–38.4%)	n/a	3.8% (1.5%–5.5%)	9.8% (4.9%–13.3%)	1.1% (0.2%–3.9%)	0.5% (0.0%–6.6%)	11.8% (6.8%–18.0%)
CAB	93.8% (43.4%–99.5%)	73.9% (59.0%–83.1%)	20.8% (7.1%–41.7%)	54.4% (34.5%–67.2%)	42.3% (27.1%–50.1%)	66.7% (46.7%–78.8%)	28.9% (15.9%–41.8%)	22.3% (11.8%–34.6%)	63.0% (45.5%–75.0%)	26.6% (7.4%–55.2%)	3.1% (0.8%–6.7%)	4.3% (1.6%–7.7%)	1.0% (0.1%–7.4%)	0.8% (0.1%–4.7%)	22.7% (9.9%–41.3%)
AXI + PEM2	96.6% (85.6%–99.0%)	71.6% (65.3%–74.9%)	8.0% (3.1%–16.0%)	30.4% (19.7%–37.1%)	47.2% (40.4%–45.1%)	56.0% (42.6%–63.9%)	31.4% (22.1%–37.6%)	17.6% (13.0%–20.8%)	39.7% (31.1%–45.3%)	27.1% (17.3%–33.3%)	3.7% (1.3%–6.0%)	9.1% (5.4%–10.2%)	1.6% (0.1%–9.2%)	0.4% (0.0%–2.1%)	19.0% (10.2%–30.0%)
PAZ	99.6% (98.3%–99.9%)	72.6% (67.4%–74.8%)	21.8% (11.3%–32.9%)	38.9% (28.8%–43.2%)	39.2% (34.1%–35.9%)	56.7% (45.7%–62.3%)	34.1% (25.9%–38.3%)	22.6% (18.3%–24.6%)	48.4% (40.7%–52.6%)	n/a	4.9% (2.2%–6.2%)	5.4% (3.7%–5.4%)	0.8% (0.1%–2.8%)	1.1% (0.5%–1.4%)	20.1% (12.1%–29.3%)
SUN	99.0% (98.6%–99.4%)	71.6% (68.9%–74.2%)	19.1% (16.0%–22.5%)	42.5% (36.0%–49.3%)	46.6% (37.8%–55.6%)	46.6% (41.5%–51.7%)	35.5% (31.0%–40.1%)	21.5% (19.0%–24.1%)	40.6% (37.0%–44.1%)	3.6% (2.7%–4.5%)	8.7% (5.1%–13.1%)	4.7% (3.1%–6.5%)	1.6% (0.9%–2.5%)	1.6% (0.9%–2.5%)	16.4% (15.0%–18.0%)
CAB + NIV	99.7% (96.9%–100.0%)	76.1% (69.2%–79.9%)	16.4% (6.6%–30.5%)	n/a	43.8% (36.0%–43.0%)	63.2% (49.4%–71.0%)	31.0% (21.3%–38.0%)	17.9% (12.8%–21.8%)	38.0% (28.7%–44.5%)	17.8% (10.0%–24.2%)	6.4% (2.1%–10.9%)	7.4% (3.8%–9.4%)	3.1% (0.1%–29.0%)	9.2% (1.2%–32.7%)	15.7% (7.8%–26.8%)
AXI + AVE	99.3% (96.1%–99.8%)	71.2% (64.9%–74.5%)	n/a	42.1% (29.5%–49.0%)	48.0% (41.4%–45.7%)	61.2% (48.0%–68.7%)	30.6% (21.6%–36.5%)	20.0% (15.1%–23.1%)	54.4% (44.9%–60.0%)	33.3% (22.0%–39.9%)	8.2% (2.9%–13.1%)	11.1% (5.9%–14.1%)	1.4% (0.1%–7.0%)	0.9% (0.3%–1.8%)	24.7% (13.8%–37.6%)
LEN + PEM	99.8% (98.4%–100.0%)	82.2% (76.3%–85.4%)	6.3% (2.4%–13.0%)	72.7% (59.6%–78.4%)	50.1% (42.5%–48.7%)	58.6% (44.9%–66.7%)	38.1% (27.3%–45.1%)	27.9% (21.3%–32.1%)	54.5% (44.4%–60.7%)	26.9% (17.0%–33.2%)	8.4% (2.9%–13.5%)	8.6% (4.9%–10.0%)	6.6% (0.6%–33.0%)	3.8% (1.4%–6.1%)	24.4% (13.5%–37.5%)

CAB, cabozantinib; PAZ, pazopanib; SOR, sorafenib; SUN, sunitinib; TIV, tivozanib; CAB + NIV, cabozantinib + nivolumab; LEN + PEM, lenvatinib + pembrolizumab; AXI + AVE, axitinib + avelumab; AXI + PEM2–axitinib + pembrolizumab; n/a–not assessable.

Tivozanib was associated with a lower risk of grade ≥3 AEs compared with sunitinib (*p* = 0.030), pazopanib (*p* = 0.019), cabozantinib + nivolumab (*p* = 0.011), and lenvatinib + pembrolizumab (*p* < 0.001) (Table 4; Supplementary Figure S2; Supplementary Table S13). A higher risk of grade ≥3 AEs was found for lenvatinib + pembrolizumab compared with most monotherapies (tivozanib, *p* < 0.001; sorafenib, *p* = 0.002; sunitinib, *p* = 0.001; pazopanib, *p* = 0.012) and axitinib combination therapies (axitinib + avelumab, *p* = 0.008; axitinib + pembrolizumab, *p* = 0.011). The adjusted mean risk of grade ≥3 AEs was the highest for lenvatinib + pembrolizumab (82.2%; 95% CI: 76.3%, 85.4%), while the lowest risk was noted for tivozanib (59.6%; 95% CI: 47.7%, 67.8%). Axitinib combination therapies were ranked the best among combination therapies; axitinib + avelumab had the lowest adjusted mean risk of grade ≥3 AEs (71.2%; 95% CI: 64.9%, 74.5%) (Table 5).

There were no differences between TKIs (monotherapy and combination therapy) in terms of treatment discontinuation due to AEs, except lenvatinib + pembrolizumab compared with sorafenib (*p* = 0.022), pazopanib (*p* = 0.030), and sunitinib (0.015). Interestingly, the adjusted mean risk of treatment discontinuation due to AEs was the highest for tivozanib (20.8%; 95% CI: 6.5%, 44.3%) sorafenib (21.6%; 95% CI: 12.5%, 29.8%), cabozantinib (20.8%; 95% CI: 7.1%, 41.7%), and pazopanib (21.8%; 95% CI: 11.3%, 32.9%), and the lowest for lenvatinib + pembrolizumab (6.3%; 95% CI: 2.4%, 13.0%) (Tables 4, 5; Supplementary Figure S2; Supplementary Table S14). Considering dose modifications due to AEs (irrespective of their relation to treatment), tivozanib reduced the risk of any dose modifications as compared with all other TKI monotherapies and all combination therapies (*p* < 0.05). On the other hand, lenvatinib + pembrolizumab increased the risk of dose modifications due to AEs compared with tivozanib, sorafenib, pazopanib, sunitinib, axitinib + pembrolizumab, and axitinib + avelumab (*p* < 0.05). The highest adjusted mean risk of dose modifications due to AEs was noted for lenvatinib + pembrolizumab (72.7%; 95% CI: 59.6%, 78.4%), while the lowest–for tivozanib (10.3%; 95% CI: 5.0%, 16.1%) (Tables 4, 5; Supplementary Figure S2; Supplementary Table S15).

#### 3.3.2 Gastrointestinal adverse events

The individual AEs reported in the references were divided into two categories: gastrointestinal (diarrhea, nausea, and vomiting) and other (fatigue, hypertension, and dysphonia). Data for any AEs and grade ≥3 AEs were presented.

Sunitinib treatment was associated with a lower risk of diarrhea of any grade as compared with sorafenib (*p* = 0.023), axitinib + avelumab (*p* = 0.030), cabozantinib + nivolumab (*p* = 0.018), and cabozantinib (*p* = 0.048) (Supplementary Figure S3; Supplementary Table S16). The adjusted mean risk of diarrhea of any grade differed between interventions. The mean risk of diarrhea of any grade was the highest for cabozantinib (66.7%; 95% CI: 46.7%, 78.8%) and cabozantinib + nivolumab (63.2%; 95% CI: 49.9%, 71.0%) and the lowest for sunitinib (46.6%; 95% CI: 41.5%, 51.7%) and tivozanib (46.8%; 95% CI: 29.4%, 60.2%) (Table 5).

For grade ≥3 diarrhea, there were no significant differences between TKIs (monotherapy and combination therapy), except the lower risk for tivozanib vs. sorafenib (*p* = 0.024) and sunitinib vs. lenvatinib + pembrolizumab (*p* = 0.032), axitinib + pembrolizumab (*p* = 0.013), sorafenib (*p* = 0.039), and axitinib + avelumab (*p* = 0.008) (Supplementary Figure S4; Supplementary Table S17). The adjusted mean risk of grade ≥3 diarrhea was generally low and differed between interventions. The mean risk of grade ≥3 diarrhea was the highest for axitinib + avelumab (11.1%; 95% CI: 5.9%, 14.1%) and the lowest for tivozanib (3.5%; 95% CI: 1.1%, 7.5%) (Table 5).

There were no differences between TKIs in the risk of nausea of any grade except for sorafenib vs. pazopanib (*p* = 0.011), sunitinib (*p* = 0.003), and lenvatinib + pembrolizumab (*p* = 0.023) (Supplementary Figure S3; Supplementary Table S18). The adjusted mean risk of nausea of any grade was similar among interventions, with the highest risk for lenvatinib + pembrolizumab (38.1%; 95% CI: 27.3%, 45.1%) and the lowest risk for sorafenib (22.4%; 95% CI: 15.8%, 26.5%) (Table 5).

There were no significant differences among TKIs used as monotherapy or as combination therapy for grade ≥3 nausea. The mean risk of grade ≥3 nausea was low and was similar among interventions, with the highest risk for lenvatinib + pembrolizumab (6.6%; 95% CI: 0.6%, 33.0%) and the lowest risk for pazopanib (0.8%; 95% CI: 0.1%, 2.8%) (Table 5; Supplementary Figure S4; Supplementary Table S19).

As for vomiting, sorafenib treatment was associated with a lower risk of vomiting of any grade compared with the other interventions such as axitinib + pembrolizumab (*p* = 0.015), cabozantinib + nivolumab (*p* = 0.015), axitinib + avelumab (*p* = 0.009), sunitinib (*p* = 0.005), cabozantinib (*p* = 0.010), pazopanib (*p* = 0.004), and lenvatinib + pembrolizumab (*p* = 0.002) (Supplementary Figure S3; Supplementary Table S20). In addition, treatment with axitinib + pembrolizumab was associated with a lower risk of vomiting than lenvatinib + pembrolizumab (*p* = 0.022). Treatment with cabozantinib + nivolumab outperformed that with lenvatinib + pembrolizumab (*p* = 0.036) (Supplementary Table S20). The mean risk of vomiting of any grade was the highest for lenvatinib + pembrolizumab (27.9%; 95% CI: 21.3%–32.1%) and the lowest for sorafenib (2.9%; 95% CI: 0.6%, 10.8%) (Table 5).

For grade ≥3 vomiting, there were no differences between TKIs (either as monotherapy or as combination therapy), except for a lower risk of vomiting for axitinib + pembrolizumab vs. cabozantinib + nivolumab (*p* = 0.039) (Supplementary Figure S4; Supplementary Table S21). The mean risk of grade ≥3 vomiting was low and was similar in all interventions, except for cabozantinib + nivolumab (9.2%; 95% CI: 1.2%, 32.7%) and lenvatinib + pembrolizumab (3.8%; 95% CI: 1.4%, 6.1%). The mean risk of grade ≥3 vomiting was the lowest for axitinib + pembrolizumab (0.4%; 95% CI: 0.0%, 2.1%) and for sorafenib (0.5%; 95% CI: 0.0%, 6.6%) (Table 5).

#### 3.3.3 Other individual adverse events

Other AEs (all grades and grade ≥3) included fatigue, hypertension, and dysphonia. There were no differences between TKIs in the risk of fatigue of any grade, except for sorafenib vs. sunitinib (*p* = 0.001), axitinib + pembrolizumab (*p* = 0.011), axitinib + avelumab (*p* = 0.006), and lenvatinib + pembrolizumab (*p* = 0.003) and for pazopanib vs. sunitinib (*p* = 0.007), axitinib + avelumab (*p* = 0.044), and lenvatinib + pembrolizumab (*p* = 0.022) (Supplementary Figure S3; Supplementary Table S22). The adjusted mean risk of fatigue of any grade was the highest for lenvatinib + pembrolizumab (50.1%; 95% CI: 42.5%, 48.7%) and axitinib + avelumab (48.0%; 95% CI: 41.4%, 45.7%), and the lowest for sorafenib (34.6%; 95% CI: 28.3%, 33.1%) (Table 5).

For grade ≥3 fatigue, there were no significant differences between TKIs (monotherapy and combination therapy) (Supplementary Figure S4; Supplementary Table S23). The mean risk of grade ≥3 fatigue was low and similar in all interventions. The lowest risk was noted for cabozantinib (3.1%; 95% CI: 0.8%, 6.7%), axitinib + pembrolizumab (3.7%; 95% CI: 1.3%, 6.0%), and sorafenib (3.8%; 95% CI: 1.5%, 5.5%) (Table 5).

Treatment with sorafenib was associated with a lower risk of hypertension of any grade as compared with pazopanib (*p* = 0.001), axitinib + avelumab (*p* = 0.001), lenvatinib + pembrolizumab (*p* = 0.002), and cabozantinib (*p* = 0.003). In addition, the following treatments were less likely to cause hypertension of any grade: 1) cabozantinib + nivolumab vs. axitinib + avelumab (*p* = 0.021), lenvatinib + pembrolimus (*p* = 0.024), and cabozantinib (*p* = 0.015); 2) axitinib + pembrolizumab vs. axitinib + avelumab (*p* = 0.030), lenvatinib + pembrolizumab (*p* = 0.034), and cabozantinib (*p* = 0.021); and 3) sunitinib vs. pazopanib (*p* = 0.047), axitinib + avelumab (*p* = 0.004), lenvatinib + pembrolizumab (*p* = 0.006), and cabozantinib (*p* = 0.012) (Supplementary Figure S3; Supplementary Table S24). The adjusted mean risk of hypertension of any grade differed between interventions. The risk was the highest for cabozantinib (63.0%; 95% CI: 45.5%, 75.0%) and the lowest for sorafenib (33.6%; 95% CI: 45.5%, 75.0%) (Table 5).

There were no significant differences between TKIs (monotherapy and combination therapy) in the risk of grade ≥3 hypertension (Supplementary Figure S4; Supplementary Table S25). The adjusted mean risk of grade ≥3 hypertension was the highest for axitinib + avelumab (24.7%; 95% CI: 13.8%, 37.6%), lenvatinib + pembrolizumab (24.4%; 95% CI: 13.5%, 37.5%), and cabozantinib (22.7%; 95% CI: 9.9%, 41.3%) and the lowest for sorafenib (11.8%; 95% CI: 6.8%, 43.1%) (Table 5).

Treatment with sorafenib was associated with a lower risk of dysphonia of any grade as compared with cabozantinib + nivolumab (*p* = 0.000), cabozantinib (*p* = 0.003), axitinib + pembrolizumab (*p* = 0.000), lenvatinib + pembrolizumab (*p* = 0.000), and axitinib + avelumab (*p* = 0.000) (Supplementary Figure S3; Supplementary Table S26). The adjusted mean risk of dysphonia of any grade differed between interventions. The risk was the highest for axitinib + avelumab (33.3%; 95% CI: 22.0%, 39.9%) and the lowest for sunitinib (3.6%; 95% CI: 2.7%, 4.5%) (Table 5).

### 3.4 Assessment of the networks

There was no heterogeneity in the NMA of vomiting (any grade and grade ≥3) and dysphonia due to network design excluding comparisons other than those with sunitinib. A low overall heterogeneity of the effect sizes was observed in the networks of any AEs (I^2^ = 0%, *p* = 0.764), grade ≥3 AEs (I^2^ = 0%, *p* = 0.383), fatigue (I^2^ = 0%, *p* = 0.655), grade ≥3 diarrhea (I^2^ = 0%, *p* = 0.625), nausea (I^2^ = 35.7%, *p* = 0.211), hypertension (I^2^ = 25.1%, *p* = 0.263), and grade ≥3 fatigue (I^2^ = 29.1%, *p* = 0.244). Moderate heterogeneity was observed in the network of grade ≥3 nausea (I^2^ = 49.8%, *p* = 0.136) and grade ≥3 hypertension (I^2^ = 49.8%, *p* = 0.141), while moderate to substantial heterogeneity was observed in the network of dose modifications due to AEs (I^2^ = 59.7%, *p* = 0.093). Considerable heterogeneity was observed only for the network of treatment discontinuation due to AEs (I^2^ = 75.1%, *p* = 0.007).

There was no significant between-design heterogeneity (inconsistency) in any network, except that of treatment discontinuation due to AEs (*p* = 0.003; Supplementary Table S27). Therefore, the results of this network should be interpreted with caution.

The evidence for the comparison of pazopanib vs. sorafenib (SWITCH II trial), sorafenib vs. sunitinib (SWITCH, CROSS-J-RCC trials), and pazopanib vs. sunitinib (COMPARZ trial) was the major contributor to the observed heterogeneity in the network of treatment discontinuation due to AEs. A considerable, but not significant, dissagreament between direct and indirect evidence was observed for those comparisons (*p*-value from 0.202 to 0.242). The relative difference between NMA results and clinical trial results was 56% for pazopanib vs. sorafenib, 35% for sorafenib vs. sunitinib, and 32% for pazopanib vs. sunitinib. A difference of more 10% between NMA results and clinical trial results for those comparisons was also found for the networks of AEs, dose modifications due to AEs, diarrhea, hypertension, grade ≥3 nausea, grade ≥3 fatigue, and grade ≥3 hypertension.

Furthermore, some dissagreement was observed for sorafenib vs. interferon α (NCT00117637) and sunitinib vs. interferon α (NCT00098657/NCT00083889 trial) in the network of dose modifications due to AEs.

Overall, the odds ratios from all networks (direct and indirect evidence combined) were similiar to direct evidence. No publication bias was found in any of the networks, however, there are too few studies to reliably assess this effect.

## 4 Discussion

In recent years, the number of approved first-line therapies for metastatic clear cell RCC has been gradually increasing. Considering the limited availability of high-quality RCTs allowing direct comparisons, there is still a strong need for a reliable indirect comparison of approved TKIs. Patients with metastatic RCC generally have poor prognosis and limited overall survival. According to clinical guidelines, the selection of therapy in metastatic RCC should be guided by disease stage, risk stratification, comorbidities, and safety profile. Most systematic reviews with NMA published to date (Hahn et al., 2019; Heo et al., 2021; Kartolo et al., 2021) focused primarily on aspects related to efficacy, assessing and comparing individual TKIs in terms of overall survival, progression free-survival, or response to treatment according to RECIST criteria. As for safety, recent NMAs were limited to general safety endpoints such as the overall frequency of AEs, grade ≥3 AEs, or treatment discontinuation due to AEs (Liu et al., 2021). So far, there were no analyses that would compare all approved TKIs (used as monotherapy and in cobmination) with respect to the risk of individual AEs.

In this study, we assessed the most common individual AEs as well as individual AEs of grade ≥3, which may have significant effects on treatment and may require additional therapy. By combining the direct and indirect evidence from 13 RCTs that also assessed TKIs in combination with immunotherapy, we were able to conduct a more comprehensive analysis, and our findings may be useful for clinicians, patients, and healthcare decision makers. Considering TKIs as monotherapy, our NMA showed that sorafenib and tivozanib were the best treatment options: sorafenib ranked highest for treatment discontinuation due to AEs, fatigue of any grade, nausea, vomiting, hypertension (any grade or grade ≥3), while tivozanib had the highest P score for any AEs, dose modifications due to AEs, and grade ≥3 diarrhea. In addition, tivozanib was associated with a significantly lower risk of grade ≥3 AEs compared with sunitinib, pazopanib, cabozantinib + nivolumab, and lenvatinib + pembrolizumab. As TKIs have antiangiogenic properties, hypertension is recognized as one of the most common side effects of this drug class and a potential marker of treatment effectiveness (Liu et al., 2021). The highest rate of hypertension of any grade and grade ≥3 was noted for cabozantinib, axitinib + avelumab, and lenvatinib + pembrolizumab, and these options were ranked as most effective based on meta-analyses by Nocera et al. (2022) and Liu et al. (2021) (assessing combination therapies) and by Manz et al. (2020) (assessing TKIs used only as monotherapy). The other results obtained in this NMA are also in line with the study by Manz et al. (2020) owed that tivozanib had the most favorable safety profile in terms of grade 3 or 4 AEs and was associated with a significantly lower risk of side effects when compared with other TKIs.

This NMA also showed that TKIs used in combination are less safe than TKIs used as monotherapy. The combination of lenvatinib and pembrolizumab was ranked as the worst option based on the highest mean risk of AEs of any grade, treatment discontinuation due to AEs, dose modifications due to AEs, and grade ≥3 nausea. There was a significantly higher risk of grade ≥3 AEs with the combination of lenvatinib and pembrolizumab compared with most monotherapies and other combination therapies. Combination therapies with axitinib were ranked as the best combination options.

Rizzo et al. (2022) conducted a meta-analysis in which they assessed the occurrence of AEs of any grade and grade ≥3 in studies comparing sunitinib monotherapy and a combination of immunotherapy with a TKI. The relative risk was similar in patients receiving combination therapy and sunitinib monotherapy. However, combination therapy was associated with an increased risk of diarrhea (any grade and grade ≥3), hypothyroidism (any grade or grade ≥3), decreased appetite (grade ≥3), increased aspartate aminotransferase levels (grade ≥3), and increased alanine transaminase levels (any grade). The results of our meta-analysis are consistent with those obtained by Rizzo et al., (2022) and suggest that the risk of treatment emergent AEs should be carefully considered when selecting a combination therapy in patients with metastatic RCC. In an NMA by Nocera et al (2022), based on a ranking quantifying the lowest likelihood of grade ≥3 AEs, sunitinib showed the lowest toxicity (*p* = 0,74), followed by axitinib + pembrolizumab (*p* = 0.47), cabozantinib + nivolumab (*p* = 0.22), and lenvatinib + pembrolizumab (*p* = 0.06) with the highest probability of grade ≥3 AEs. In an NMA by Quhal et al. (2021), the highest probability of treatment discontinuation related to AEs was shown for lenvatinib in combination with pembrolizumab. This was in contrast to an NMA by Liu et al. (2021), in which the most severe AEs were associated with axitinib in combinaiton with pembrolizumab.

NMA conducted by Manz et al. (2020) showed that cabozantinib, sunitinib, pazopanib, and tivozanib do not differ significantly in terms of efficacy, but tivozanib was associated with a more favorable safety profile in terms of grade ≥3 toxicity, simillary as in our NMA. Therefore, the relative toxicity of these first-line TKIs may play a more significant role than comparisons of efficacy in treatment decisions and planning future clinical trials (Nocera et al., 2022).

Our meta-analysis has some limitations that should be considered when interpreting the results. First, we included only studies on TKIs approved by the European Medicines Agency (EMA) or the Food and Drug Administration (FDA) for use as monotherapy or in combination for the first-line treatment of patients with metastatic RCC. Studies concerning, for example, the use of axitinib as monotherapy in previously untreated patients were excluded, because axitinib is currently approved for use as first-line treatment only in combination with avelumab or pembrolizumab. Second, some of the assessed interventions may differ in terms of efficacy (Heo et al., 2021), because these drugs are usually used until disease progression or unacceptable toxicity occurs, which may result in a different duration of exposure to treatment. On the one hand, a more appropriate measure in this scenario would be a comparison of exposure-adjusted incidence rate of AEs, especially in studies with long-term follow-up (Kartolo et al., 2021), but on the other hand, most of the published studies assessing TKIs reported only the percentages of patients experiencing AEs. To avoid potential diffrences in the duration of exposure to the same intervention between different studies due to different baseline characteristics of patients, inclusion criteria in our review were limited to the stage of the disease (metastatic), the line of treatment (first line) and the histological type of cancer (clear cell). The included studies were generally well balanced. Due to the similar mechanism of action, the differences in the duration of exposure to treatment between TKI monotherapies in the included studies were relatively small. The longest duration of exposure to treatment was observed for studies evaluating the combinations of TKIs with immunotherapy, which may be one of the reasons for the generally worse safety profile of combination therapies vs. monotherapies with TKIs. Another reason is that patients receiving combination therapies are treated with two drugs with different mechanisms of action and overlapping adverse reactions. We included only RCTs because they have the highest credibility. Nevertheless, in included trials, people involved in safety assessment (both patients and physicians) were not blinded (in some studies, only the persons/committee who assess the results for the primary end point, i.e., survival rates, were blinded). This was the main reason why the risk of performance bias was assessed as high. It can be assumed that the risk of bias related to incomplete blinding was similar in all included studies. There was some disagreement between direct and indirect evidence in pazopanib trials, for example, in terms of the rate of grade ≥3 nausea: the CROSS-J-RCC trial reported a rate of 0%, while other trials reported some cases of nausea. Some differences in baseline characteristics between pazopanib trials may cause the heterogeneity of results. The higher rate of grade ≥3 nausea in the TemPa trial may be due to the fact that >50% of patients had an ECOG performance status of 2, while in the remaining studies on pazopanib, <50% of patients had an ECOG performance status of 0. Furthermore, not all included trials reported the assessed safety outcomes. The results from clinical trials registries could not be used, because they report these endpoints in a different way: only serious AEs or nonserious AEs. Furthermore, the results presented in registries are not official results, and, by definition, they have lower reliability than data from full-text publications. Sometimes, individual but rare AEs are not reported because of the threshold used in a publication (e.g., only AEs that occurred in at least 10% or 20% of patients in either group). Therefore, it was impossible to conduct an NMA in terms of grade ≥3 dysphonia.

According to the latest clinical ASCO, ESMO and NCCN guidelines for the treatment of metastatic clear cell RCC, the TKIs still play an important role in the first-line setting (Rathmell et al., 2022; European Society for Medical Oncology, 2022; Motzer et al., 2022b). Patients with favorable-risk disease who require systemic therapy may be offered an immunotherapy with an immune checkpoint inhibitor in combination with a vascular endothelial growth factor receptor (VEGFR TKI); patients with intermediate or poor risk should be offered a doublet regimen (immune checkpoint inhibitor in combination with a VEGFR TKI or TKIs as monotherapy). For selected patients, monotherapy with either an immune checkpoint inhibitor or a VEGFR TKI may be offered depending on comorbidities and general health (Rathmell et al., 2022; Motzer et al., 2022b).

In summary, when choosing the appropriate therapy for individual patients, clinicians should consider the overall safety profile of TKIs as well as the prevalence of the most common AEs (particularly specific AEs), rather than looking at efficacy. Despite several limitations, this systematic review with NMA is the first original study to provide new data on the relative safety of various TKIs, focusing on the AEs (all grades and grade ≥3), treatment discontinuation due to AEs, dose modification due to AEs, and the risk of specific AEs that are most commonly listed in the summary of products characteristics (i.e., fatigue, diarrhea, nausea, vomiting, hypertension, and dysphonia). Since this approach has not been used in previous systematic reviews, our study provides the most up-to-date results in terms of an in-depth comparative safety analysis of TKIs used alone or in combination. Our findings underscore the importance of considering monotherapy with TKIs as the preferred way to achieve improved safety outcomes, especially when compared with combination therapy based on immune drugs. The results may help clinicians and patients choose the best treatment option from a wide range of available TKIs. Moreover, they may serve as guidance for healthcare policymakers in developing reimbursement policies.

## Data Availability

The original contributions presented in the study are included in the article/Supplementary Material, further inquiries can be directed to the corresponding author.
