# Supplementary material for: Comparative safety of tyrosine kinase inhibitors in the treatment of metastatic renal cell carcinoma: a systematic review and network meta-analysis
Source: Front Pharmacol. 2023 Sep 7;14:1223929. doi: 10.3389/fphar.2023.1223929 (PMC10512702; doi:10.3389/fphar.2023.1223929)
Supplement: Supplementary file 1 [file DataSheet1.docx]

# ***Supplementary Materials***

**Comparative safety of tyrosine kinase inhibitors in the treatment of metastatic renal cell carcinoma: a systematic review and network meta-analysis**

Kinga Krawczyk^1^, Katarzyna Śladowska^2^, Przemysław Holko^2^, Paweł Kawalec^2*^

^1^ Faculty of Health Sciences, Institute of Public Health, Jagiellonian University Medical College, Krakow, Poland

^2^ Department of Nutrition and Drug Research, Faculty of Health Sciences, Institute of Public Health, Jagiellonian University Medical College, Krakow, Poland

*** Correspondence:** Corresponding Author: pawel.kawalec@uj.edu.pl

## **Search strategy**

**Supplementary Table 1. Search strategy used in PubMed, EMBASE and Cochrane Library for tyrosine kinase inhibitors used in patients with metastatic renal cell carcinoma (last search: November 2022).**

| **Number** | **Keywords** | **Search result** | | |
| --- | --- | --- | --- | --- |
|  |  | **PubMed^1^** | **Embase^2^** | **Cochrane^3^** |
| **Population** | | | | |
| **#1** | *(kidney OR renal) AND (carcinoma OR carcinomas OR cancer OR cancers OR neoplasm OR neoplasms OR tumor OR tumors OR tumour OR tumours OR neoplasia) ^1, 3^*  *kidney OR renal AND (carcinoma OR carcinomas OR cancer OR cancers OR neoplasm OR neoplasms OR tumor OR tumors OR tumour OR tumours OR neoplasia) ^2^* | 237 924 | 239 051 | 15 889 |
| **#2** | *(renal OR kidney) AND cell carcinoma ^1, 3^*  *renal OR kidney AND cell AND carcinoma ^2^* | 73 332 | 120 442 | 4 772 |
| **#3** | *RCC ^1, 3^*  *rcc ^2^* | 22 466 | 32 286 | 1 940 |
| **#4** | ***#1 OR #2 OR #3*** | **243 575** | **393 385** | **15 973** |
| **TKIs** | | | | |
| **#5** | *tivozanib OR fotivda OR ‘AV 951’ OR ‘AV951 cpd’ OR ‘AV-951’ OR ‘KRN 951’ OR KRN951 ^1, 2, 3^* | 155 | 838 | 112 |
| **#6** | *Sunitinib OR sutent OR ‘suo 11248’ OR ‘suo11248’ OR ‘pha2909040ad’* | 7 160 | 27 047 | 1 360 |
| **#7** | *Sorafenib OR nexavar OR ‘BAY 545-9085’ OR ‘BAY-545-9085’ OR ‘BAY 43-9006’ OR bay4390*  *(Sorafenib OR nexavar OR ‘BAY 545 9085’ OR ‘BAY545 9085’ OR ‘BAY 43 9006’ OR bay4390) ^3^* | 11 121 | 36 672 | 2 061 |
| **#8** | *Pazopanib OR votrient OR ‘gw 786034’ OR gw786034 OR ‘GW 786034B’ OR GW786034B OR ‘GW-786034B’ OR ‘GW 780604’ OR GW780604* | 2 206 | 10 161 | 623 |
| **#9** | *Cabozantinib OR Cometriq OR ‘XL 184’ OR ‘XL-184’ OR ‘BMS 907351’ OR BMS907351 OR ‘BMS-907351’* | 1 463 | 6 309 | 532 |
| **#10** | *Lenvatinib OR Lenvima OR ‘E 7080’ OR ‘E-7080’ OR ‘ER-203492-00’ OR E7080*  *(Lenvatinib OR Lenvima OR ‘E 7080’ OR ‘E7080’ OR ‘ER 203492 00’ OR E7080) ^3^* | 1 720 | 5 385 | 750 |
| **#11** | *Axitinib OR inlyta OR ‘AG 013736’ OR AG013736 OR ‘AG-013736’* | 1 394 | 6 705 | 388 |
| **#12** | ***#5 OR #6 OR #7 OR #8 OR #9 OR #10 OR #11*** | **20 327** | **62 376** | **4 752** |
| **Interventions + population** | | | | |
| **#13** | ***#4 AND #12*** | **5 690** | **18 743** | **1 672** |
| **#14** | ***#13**** | **282** | **623** | **1 272** |
| **#15** | ***#14^*** | **268** | **605** | **-** |

TKIs – tyrosine kinase inhibitors; **filters used in PubMed = Humans; Randomized controlled trial, Controlled clinical trial; filters used in Embase = Humans, Randomized controlled trial, Controlled clinical trial AND [embase]/lim NOT [medline]/lim; filters used in Cochrane: AND randomized OR random OR RCT OR randomly OR randomization OR allocation OR allocated, Cochrane Central Register of Controlled Trials; ^ languages: English.*

**Supplementary Table 2. Search strategy for tyrosine kinase inhibitors used in patients with metastatic renal cell carcinoma on the website of clinical trials (last search: November 2022).**

| **Register (website)** | **Keywords** | **Search results** |
| --- | --- | --- |
| **Clinicaltrials.gov**  **https://clinicaltrials.gov/)** | *(tivozanib OR sunitinib OR sorafenib OR pazopanib OR cabozantinib OR lenvatinib OR axitinib) AND (Renal Cell Carcinoma OR RCC)* | *541*  *Study type: Interventional (clinical trial): 429* |

## **Detailed inclusion criteria**

**Supplementary Table 3. Detailed Inclusion and exclusion criteria for systematic review and meta-analysis.**

|  | **Inclusion criteria** | **Exclusion criteria** |
| --- | --- | --- |
| **Population** | Adult patients with majority of patients with metastatic clear cell RCC (or with clear cell component) not previously treated systemically (except surgery: trials with ≥70% of patients previously untreated were eligible; the local treatment such surgery or radiotherapy were allowed) | Pediatric patients with RCC, patients previously treated, trials with no information about line of therapy, trials with patients with other than clear cell RCC |
| **Intervention and comparators** | Registered TKIs in monotherapy or in combination therapy (details about dosing provided in table below): tivozanib, sunitinib, sorafenib, pazopanib, cabozantinib + nivolumab, lenvatinib + pembrolizumab, axitinib + avelumab, axitinib + pembrolizumab compared to each other or with placebo or with other therapy registered by EMA or FDA for first line treatment of RCC | Interventions not of interest (eg not approved for metastatic RCC); trials not including directly comparison of safety of any of the mentioned interventions to any other included TKI or placebo or other therapy registered by EMA or FDA for first line treatment of RCC |
| **Outcomes** | Adverse events, grade ≥3 adverse events , discontinuation because adverse events, dose modification due to adverse events, individual adverse events (all grades and grade ≥3): fatigue, diarrhea, nausea, vomiting, hypertension, dysphonia | Trials or additional articles for included trials not reported defined outcomes |
| **Study types** | Randomized controlled trials | Non-randomized controlled, trials, observational studies, case reports, reviews, additional analysis of included trials, additional references for included trials without safety data or without newer safety data than reported in main publication, cross-sectional studies |
| **Treatment period** | Duration of treatment: disease progression or as long as clinical benefit is observed or until unacceptable toxicity occurs | Trials in which the length of the treatment period was predetermined/restricted, regardless of progression, toxicity, or treatment benefit |
| **Publication type** | Full text articles, data from clinical trials registers were allowed to use | Abstracts, posters, editorials, letters |
| **Language** | English | Languages other than English |

EMA - European Medicines Agency; FDA - Food and Drug Administration; TKIs – tyrosine kinase inhibitors; RCC – renal cell carcinoma.

**Supplementary Table 4. TKIs registered by EMA and/or FDA at first line treatment of RCC with their dosing and administration route.**

| **TKIs– active substance** | **Trade name** | **Dosing and administration route** |
| --- | --- | --- |
| **Tyrosine kinase inhibitors - monotherapy** | | |
| cabozantinib (EMA, FDA) | Cabometyx^®^ | Dosing: cabozantinib at a dose of 60 mg once a day (PO)  Duration of treatment: until the patient is no longer clinically benefiting from therapy or until unacceptable toxicity occurs |
| pazopanib (FDA, EMA) | Votrient^®^ | Dosing: pazopanib at a dose of 800 mg once a day (PO)  Duration of treatment: no data |
| sorafenib (EMA, FDA) | Nexavar^®^ | Dosing: sorafenib at a dose of 400 mg (two tablets of 200 mg) twice a day (equivalent to a total daily dose of 800 mg)  Duration of treatment: as long as clinical benefit is observed or until unacceptable toxicity occurs |
| sunitinib (EMA, FDA) | Sutant, Sunitinib accord^®^ | Dosing: sunitinib at a dose of 50 mg once a day (PO), for 4 weeks, followed by a 2 weeks without treatment (Schedule 4/2) to comprise a complete cycle of 6 weeks  Duration of treatment: until disease progression |
| tivozanib (EMA, FDA) | Fotivda^®^ | Dosing: tivozanib at a dose of 1340 microgram once a day (PO) for 21 days, followed by a 7 day without treatment to comprise one complete treatment cycle of 4 weeks  Duration of treatment: until disease progression or unacceptable toxicity |
| **Tyrosine kinase inhibitors – combined therapy** | | |
| cabozantinib + nivolumab (EMA, FDA) | Cabometyx^®^ in combination with nivolumab | Dosing: cabozantinib at a dose of 40 mg once a day (PO) in combination with nivolumab administered intravenously at either 240 mg every 2 weeks or 480 mg every 4 weeks.  Duration of treatment: cabozantinib until disease progression or unacceptable toxicity. Nivolumab should be continued until disease progression, unacceptable toxicity, or up to 24 months in patients without disease progression |
| lenvatinib + pembrolizumab (EMA, FDA) | Kisplyx^®^/Lenvima^®^ in combination with pembrolizumab | Dosing: lenvatinib at a dose of 20 mg (two 10-mg capsules) (PO) once a day in combination with pembrolizumab either 200 mg every 3 weeks or 400 mg every 6 weeks administered as an intravenous infusion over 30 minutes  Duration of treatment: lenvatinib treatment should continue until disease progression or unacceptable toxicity. Pembrolizumab should be continued until disease progression, unacceptable toxicity or the maximum duration of therapy as specified for pembrolizumab |
| axitinib + avelumab (EMA*, FDA*) | Inlyta^®^ in combination with avelumab | Dosing: axitinib at a dose of 5 mg (PO) twice a day with avelumab intravenously at a dose of 800 mg every 2 weeks.  Duration of treatment: until disease progression or unacceptable toxicity |
| axitinib + pembrolizumab (EMA*, FDA*) | Inlyta^®^  in combination with pembrolizumab | Dosing: axitinib at a dose of 5 mg (PO) twice a day with pembrolizumab intravenously at a dose of 200 mg every 3 weeks or 400 mg every 6 weeks  Duration of treatment: until disease progression or unacceptable toxicity |

Sources: EMA (European Medicines Agency https://www.ema.europa.eu/en) and FDA (Food and Drug Administration https://www.fda.gov/) websites; PO – per os (orally); TKIs - tyrosine kinase inhibitors. *according to EMA and FDA, axitinib in monotherapy is registered only for the treatment of adult patients with advanced renal cell carcinoma (RCC) after failure of prior treatment with sunitinib or a cytokine.

## **List of excluded trials**

**Supplementary Table 5. List of trials excluded from systematic review and network meta-analysis after full text analysis.**

| **Reason for exclusion** | **References** |
| --- | --- |
| **Inappropriate population** | Pal, SK., et al. (2021). A comparison of sunitinib with cabozantinib, crizotinib, and savolitinib for treatment of advanced papillary renal cell carcinoma: a randomised, open-label, phase 2 trial. Lancet . Feb 20;397(10275):695-703.  Bergmann, L., et al. (2020). A Randomized Phase IIa Trial with Temsirolimus versus Sunitinib in Advanced Non-Clear Cell Renal Cell Carcinoma: An Intergroup Study of the CESAR Central European Society for Anticancer Drug Research-EWIV and the Interdisciplinary Working Group on Renal Cell Cancer (IAGN) of the German Cancer Society. Oncol Res Treat;43(7-8):333-339.  Choueiri, TK., et al. (2020). Efficacy of Savolitinib vs Sunitinib in Patients With MET-Driven Papillary Renal Cell Carcinoma: The SAVOIR Phase 3 Randomized Clinical Trial . JAMA Oncol . Aug 1;6(8):1247-1255.  Armstrong, AJ., et al. (2016). Everolimus versus sunitinib for patients with metastatic non-clear cell renal cell carcinoma (ASPEN): a multicentre, open-label, randomised phase 2 trial . Lancet Oncol . Mar;17(3):378-388.  Tannir, NM., et al. (2016). Everolimus Versus Sunitinib Prospective Evaluation in Metastatic Non-Clear Cell Renal Cell Carcinoma (ESPN): A Randomized Multicenter Phase 2 Trial . Eur Urol . May;69(5):866-74.  Zhao, F., et al. (2018). Fatigue among patients with renal cell carcinoma receiving adjuvant sunitinib or sorafenib: patient-reported outcomes of ECOG-ACRIN E2805 trial. Support Care Cancer. Jun;26(6):1889-1895.  Haas, NB., et al. (2016). Adjuvant sunitinib or sorafenib for high-risk, non-metastatic renal-cell carcinoma (ECOG-ACRIN E2805): a double-blind, placebo-controlled, randomised, phase 3 trial. Lancet. May 14;387(10032):2008-16.  Motzer, RJ., et al; PROTECT study investigators. (2021). Adjuvant Pazopanib Versus Placebo After Nephrectomy in Patients With Localized or Locally Advanced Renal Cell Carcinoma: Final Overall Survival Analysis of the Phase 3 PROTECT Trial. Eur Urol. Mar;79(3):334-338.  Motzer, RJ., et al; PROTECT investigators. (2017). Randomized Phase III Trial of Adjuvant Pazopanib Versus Placebo After Nephrectomy in Patients With Localized or Locally Advanced Renal Cell Carcinoma. J Clin Oncol. = Dec 10;35(35):3916-3923.  Sternberg, CN., et al. (2018). Pazopanib Exposure Relationship with Clinical Efficacy and Safety in the Adjuvant Treatment of Advanced Renal Cell Carcinoma. Clin Cancer Res. Jul 1;24(13):3005-3013.  Eisen, T., et al. (2020). Adjuvant Sorafenib for Renal Cell Carcinoma at Intermediate or High Risk of Relapse: Results From the SORCE Randomized Phase III Intergroup Trial. J Clin Oncol. Dec 1;38(34):4064-4075.  Wood, SY., et al. (2020). Understanding the adverse event experience in the S-TRAC adjuvant trial of sunitinib for high-risk renal cell carcinoma. Future Oncol. Feb;16(4):39-47.  Ravaud, A., et al. (2016). Adjuvant Sunitinib in High-Risk Renal-Cell Carcinoma after Nephrectomy. N Engl J Med Dec 8;375(23):2246-2254.  Staehler, M.,et al. (2018). Adjuvant sunitinib in patients with high-risk renal cell carcinoma: safety, therapy management, and patient-reported outcomes in the S-TRAC trial. Ann Oncol. Oct 1;29(10):2098-2104.  George, DJ.,et al. (2019). Phase III Trial of Adjuvant Sunitinib in Patients with High-Risk Renal Cell Carcinoma: Exploratory Pharmacogenomic Analysis. Clin Cancer Res. Feb 15;25(4):1165-1173.  Gross-Goupil, M.,et al. (2018). Axitinib versus placebo as an adjuvant treatment of renal cell carcinoma: results from the phase III, randomized ATLAS trial. Ann Oncol. Dec 1;29(12):2371-2378. |
| **Inappropriate population - results only in specific subpopulations** | Tamada, S.,et al. (2022). Pembrolizumab plus axitinib versus sunitinib in metastatic renal cell carcinoma: outcomes of Japanese patients enrolled in the randomized, phase III, open-label KEYNOTE-426 study. Int J Clin Oncol. Jan;27(1):154-164.  Tomita, Y.,et al. (2022). Efficacy and safety of avelumab plus axitinib in elderly patients with advanced renal cell carcinoma: extended follow-up results from JAVELIN Renal 101. ESMO Open. Apr;7(2):100450.  Uemura, M.,et al. (2020). Avelumab plus axitinib vs sunitinib for advanced renal cell carcinoma: Japanese subgroup analysis from JAVELIN Renal 101. Cancer Sci. Mar;111(3):907-923.  Albiges, L.,et al. (2022). First-line Nivolumab plus Ipilimumab Versus Sunitinib in Patients Without Nephrectomy and With an Evaluable Primary Renal Tumor in the CheckMate 214 Trial. Eur Urol. Mar;81(3):266-271.  Tomita, Y.,et al. (2020). Nivolumab plus ipilimumab versus sunitinib in previously untreated advanced renal-cell carcinoma: analysis of Japanese patients in CheckMate 214 with extended follow-up. Jpn J Clin Oncol. Jan 24;50(1):12-19. |
| **Inappropriate line of treatment (patients previously treated systemically - pharmacologically)** | Grünwald, V.,et al. (2022). A Prospectivly Randomized Phase-II Trial of Axitinib versus Everolimus as Second-Line Therapy in Metastatic Renal Cell Carcinoma (BERAT Study). Oncol Res Treat.;45(5):272-280.  Voss, MH.,et al. (2019). A phase 2, randomized trial evaluating the combination of dalantercept plus axitinib in patients with advanced clear cell renal cell carcinoma. Cancer. Jul 15;125(14):2400-2408.  Escudier, B.,et al. (2014). Axitinib versus sorafenib in advanced renal cell carcinoma: subanalyses by prior therapy from a randomised phase III trial. Br J Cancer. Jun 10;110(12):2821-8. AXIS  Donskov, F.,et al. (2020). Outcomes based on age in the phase III METEOR trial of cabozantinib versus everolimus in patients with advanced renal cell carcinoma. Eur J Cancer. Feb;126:1-10.  Choueiri, TK.,et al; METEOR investigators. (2016). Cabozantinib versus everolimus in advanced renal cell carcinoma (METEOR): final results from a randomised, open-label, phase 3 trial. Lancet Oncol. Jul;17(7):917-927.  Choueiri, TK., et al; METEOR Investigators. (2015). Cabozantinib versus Everolimus in Advanced Renal-Cell Carcinoma. N Engl J Med. Nov 5;373(19):1814-23.  Rini, BI.,et al. (2020). Tivozanib versus sorafenib in patients with advanced renal cell carcinoma (TIVO-3): a phase 3, multicentre, randomised, controlled, open-label study. Lancet Oncol. Jan;21(1):95-104.  Pal, SK.,et al. (2020). Final Overall Survival Results from a Phase 3 Study to Compare Tivozanib to Sorafenib as Third- or Fourth-line Therapy in Subjects with Metastatic Renal Cell Carcinoma. Eur Urol. Dec;78(6):783-785.  Pal, SK.,et al. (2022). Assessing the Safety and Efficacy of Two Starting Doses of Lenvatinib Plus Everolimus in Patients with Renal Cell Carcinoma: A Randomized Phase 2 Trial. Eur Urol. Sep;82(3):283-292.  Kollmannsberger, C., et al. (2021). A Randomized Phase II Study of AGS-16C3F Versus Axitinib in Previously Treated Patients with Metastatic Renal Cell Carcinoma. Oncologist. Mar;26(3):182-e361.  Motzer, RJ.,et al. (2016). Independent assessment of lenvatinib plus everolimus in patients with metastatic renal cell carcinoma. Lancet Oncol. Jan;17(1):e4-5.  Motzer, RJ., et al. (2015). Lenvatinib, everolimus, and the combination in patients with metastatic renal cell carcinoma: a randomised, phase 2, open-label, multicentre trial. Lancet Oncol. Nov;16(15):1473-1482. |
| **Inappropriate project of the trial (therapy in the comparator arm not registered in the first line)** | Lee, JL., et al. (2015). RandomizEd phase II trial of Sunitinib four weeks on and two weeks off versus two weeks on and one week off in metastatic clear-cell type REnal cell carcinoma: RESTORE trial. Ann Oncol.;26:2300–5.  Motzer, RJ., et al. (2012). Randomized phase II trial of sunitinib on an intermittent versus continuous dosing schedule as first-line therapy for advanced renal cell carcinoma. J Clin Oncol;30:1371–7.  Cella, D., et al. (2015). Fatigue in patients with advanced renal cell carcinoma receiving sunitinib on an intermittent versus continuous dosing schedule in a randomized phase II trial. Cancer medicine, 3(5), 1353‐1358.  Cirkel, GA., etal. (2017). Alternating Treatment With Pazopanib and Everolimus vs Continuous Pazopanib to Delay Disease Progression in Patients With Metastatic Clear Cell Renal Cell Cancer: The ROPETAR Randomized Clinical Trial. JAMA Oncol. Apr 1;3(4):501-508.  Motzer, RJ., et al. (2014). Phase II randomized trial comparing sequential first-line everolimus and second-line sunitinib versus first-line sunitinib and second-line everolimus in patients with metastatic renal cell carcinoma. J Clin Oncol. Sep 1;32(25):2765-72.  Zhou, AP., et al. (2019). Anlotinib Versus Sunitinib as First-Line Treatment for Metastatic Renal Cell Carcinoma: A Randomized Phase II Clinical Trial. Oncologist. Aug;24(8):e702-e708.  Hutson, TE.,et al. (2013). Axitinib versus sorafenib as first-line therapy in patients with metastatic renal-cell carcinoma: a randomised open-label phase 3 trial. Lancet Oncol. Dec;14(13):1287-94.  Hutson, TE., et al. (2017). Axitinib Versus Sorafenib in First-Line Metastatic Renal Cell Carcinoma: Overall Survival From a Randomized Phase III Trial. Clin Genitourin Cancer. Feb;15(1):72-76.  Sheng, X., et al. (2019). First-line axitinib versus sorafenib in Asian patients with metastatic renal cell carcinoma: exploratory subgroup analyses of Phase III data. Future Oncol. Jan;15(1):53-63.  Eisen, T., et al. (2015). A randomised, phase II study of nintedanib or sunitinib in previously untreated patients with advanced renal cell cancer: 3-year results. Br J Cancer. Oct 20;113(8):1140-7.  Rodriguez-Vida, A., et al. (2020). Randomised Phase II study comparing alternating cycles of sunitinib and everolimus vs standard sequential administration in first-line metastatic renal carcinoma (SUNRISES study). BJU Int;126(5):559-567.  Escudier, B.,et al. (2014). Randomized, controlled, double-blind, cross-over trial assessing treatment preference for pazopanib versus sunitinib in patients with metastatic renal cell carcinoma: PISCES Study. J Clin Oncol. May 10;32(14):1412-8.  Négrier, S., et al. (2011). Temsirolimus and bevacizumab, or sunitinib, or interferon alfa and bevacizumab for patients with advanced renal cell carcinoma (TORAVA): a randomised phase 2 trial. Lancet Oncol. Jul;12(7):673-80.  Abdelaziz, LA., et al. (2021). Tolerability and outcome of sunitinib by giving 4/2 schedule versus 2/1 schedule in metastatic renal cell carcinoma patients: a prospective randomized multi-centric Egyptian study. Wspolczesna onkologia, 24(4), 221‐228.  Motzer, RJ., et al. (2018). Nivolumab plus Ipilimumab versus Sunitinib in Advanced Renal-Cell Carcinoma. N Engl J Med Apr 5;378(14):1277-1290.  Albiges, L., et al. (2020). Nivolumab plus ipilimumab versus sunitinib for first-line treatment of advanced renal cell carcinoma: extended 4-year follow-up of the phase III CheckMate 214 trial. ESMO Open. Nov;5(6):e001079.  Motzer, RJ., et al. (2019). Nivolumab plus ipilimumab versus sunitinib in first-line treatment for advanced renal cell carcinoma: extended follow-up of efficacy and safety results from a randomised, controlled, phase 3 trial. Lancet Oncol. 2019 Oct;20(10):1370-1385.  Cella, D.,et al. (2019). Patient-reported outcomes of patients with advanced renal cell carcinoma treated with nivolumab plus ipilimumab versus sunitinib (CheckMate 214): a randomised, phase 3 trial. Lancet Oncol. Feb;20(2):297-310.  Tannir, NM., et al. (2021). Efficacy and safety of nivolumab plus ipilimumab versus sunitinib in first-line treatment of patients with advanced sarcomatoid renal cell carcinoma. Clin Cancer Res. Jan 1;27(1):78-86.  Regan, MM., et al. (2021). Treatment-free Survival after Immune Checkpoint Inhibitor Therapy versus Targeted Therapy for Advanced Renal Cell Carcinoma: 42-Month Results of the CheckMate 214 Trial Clinical cancer research;27(24):6687-6695.  Motzer, RJ., et al. (2022). Conditional survival and long-term efficacy with nivolumab plus ipilimumab versus sunitinib in patients with advanced renal cell carcinoma. Cancer. Jun 1;128(11):2085-2097.  Motzer, RJ., et al. (2020). Survival outcomes and independent response assessment with nivolumab plus ipilimumab versus sunitinib in patients with advanced renal cell carcinoma: 42-month follow-up of a randomized phase 3 clinical trial. J Immunother Cancer. Jul;8(2):e000891.  Pal, SK., et al. (2020). Patient-reported outcomes in a phase 2 study comparing atezolizumab alone or with bevacizumab vs sunitinib in previously untreated metastatic renal cell carcinoma. BJU Int. Jul;126(1):73-82.  McDermott, DF., et al. (2018). Clinical activity and molecular correlates of response to atezolizumab alone or in combination with bevacizumab versus sunitinib in renal cel carcinoma. Nat Med; 24: 749–57.  Atkins, MB., et al. (2020). Patient-Reported Outcomes from the Phase III Randomized IMmotion151 Trial: Atezolizumab + Bevacizumab versus Sunitinib in Treatment-Naïve Metastatic Renal Cell Carcinoma. Clin Cancer Res. Jun 1;26(11):2506-2514.  Motzer, RJ., et al. (2022). Final Overall Survival and Molecular Analysis in IMmotion151, a Phase 3 Trial Comparing Atezolizumab Plus Bevacizumab vs Sunitinib in Patients With Previously Untreated Metastatic Renal Cell Carcinoma. JAMA Oncol. Feb 1;8(2):275-280.  Rini, BI., et al. (2019). Atezolizumab plus bevacizumab versus sunitinib in patients with previously untreated metastatic renal cell carcinoma (IMmotion151): a multicentre, open-label, phase 3, randomised controlled trial. Lancet. Jun 15;393(10189):2404-2415.  Figlin, RA.,et al; ADAPT study group. (2020). Results of the ADAPT Phase 3 Study of Rocapuldencel-T in Combination with Sunitinib as First-Line Therapy in Patients with Metastatic Renal Cell Carcinoma. Clin Cancer Res. May 15;26(10):2327-2336.  Rini, BI., et al. (2016). IMA901, a multipeptide cancer vaccine, plus sunitinib versus sunitinib alone, as first-line therapy for advanced or metastatic renal cell carcinoma (IMPRINT): a multicentre, open-label, randomised, controlled, phase 3 trial. Lancet Oncol. Nov;17(11):1599-1611.  Procopio, G., et al; Italian Trials in Medical Oncology (ITMO) group. (2013). Overall survival for sorafenib plus interleukin-2 compared with sorafenib alone in metastatic renal cell carcinoma (mRCC): final results of the ROSORC trial. Ann Oncol. Dec;24(12):2967-71.  Jonasch, E., et al et al. (2010). Upfront, randomized, phase 2 trial of sorafenib versus sorafenib and low-dose interferon alfa in patients with advanced renal cell carcinoma: clinical and biomarker analysis. Cancer. Jan 1;116(1):57-65.  Lindskog, M., et al. (2022). Ilixadencel, a Cell-based Immune Primer, plus Sunitinib Versus Sunitinib Alone in Metastatic Renal Cell Carcinoma: a Randomized Phase 2 Study. European urology open science, 40, 38‐45.  Hainsworth, JD., et al. (2016). A Randomized, Open-Label Phase 2 Study of the CXCR4 Inhibitor LY2510924 in Combination with Sunitinib Versus Sunitinib Alone in Patients with Metastatic Renal Cell Carcinoma (RCC). Target Oncol. Oct;11(5):643-653.  Ratain, MJ., et al. (2006). Phase II placebo-controlled randomized discontinuation trial of sorafenib in patients with metastatic renal cell carcinoma. J Clin Oncol;24(16):2505-12.  Nosov, DA., et al. (2012). Antitumor activity and safety of tivozanib (AV-951) in a phase II randomized discontinuation trial in patients with renal cell carcinoma. J Clin Oncol;30: 1678–85.  Tomita, Y., et al. (2019). Patients with metastatic renal cell carcinoma who benefit from axitinib dose titration: analysis from a randomised, double-blind phase II study. BMC Cancer. Jan 7;19(1):17  Rini, BI., et al. (2015). Axitinib dose titration: analyses of exposure, blood pressure and clinical response from a randomized phase II study in metastatic renal cell carcinoma. Ann Oncol. 2015 Jul;26(7):1372-7.  Kollmannsberger, C., et al. (2021). A Randomized Phase II Study of AGS-16C3F Versus Axitinib in Previously Treated Patients with Metastatic Renal Cell Carcinoma. Oncologist. Mar;26(3):182-e361.  Sternberg, CN., et al. (2014). An open-label extension study to evaluate safety and efficacy of pazopanib in patients with advanced renal cell carcinoma. Oncology;87(6):342-50.  Sternberg, CN., et al. (2013). A randomised, double-blind phase III study of pazopanib in patients with advanced and/or metastatic renal cell carcinoma: final overall survival results and safety update. Eur J Cancer. Apr;49(6):1287-96.  Sternberg, CN., et al. (2010). Pazopanib in locally advanced or metastatic renal cell carcinoma: results of a randomized phase III trial. J Clin Oncol. Feb 20;28(6):1061-8.  Voss, MH., et al. (2019). A phase 2, randomized trial evaluating the combination of dalantercept plus axitinib in patients with advanced clear cell renal cell carcinoma. Cancer. Jul 15;125(14):2400-2408.  Sternberg, CN.,et al. (2014). An open-label extension study to evaluate safety and efficacy of pazopanib in patients with advanced renal cell carcinoma. Oncology;87(6):342-50.  Liu, C., et al. (2017). Efficacy of Sunitinib in the Treatment of Advanced Renal Cell Carcinoma and Its Effect on Survival Time. Anti-Tumor Pharmacy 7:2 (195-199)  Wang, Q., et al . (2019). Analysis of efficacy of sorafenib combined with vascular endothelial growth factor inhibitor Avastin in renal cell carcinoma. Journal of B.U.ON, 24(4), 1638‐1643. |
| **Abstracts; additional references for which the full text is available** | Motzer, RJ., et al. (2011). Randomized phase II multicenter study of the efficacy and safety of sunitinib on the 4/2 versus continuous dosing schedule as first-line therapy of metastatic renal cel carcinoma: Renal Effect Trial. J Clin Oncol;29:LBA308. |
| **No endpoints/**  **outcomes of interest** | Choueiri, TK., et al. (2020). Updated efficacy results from the JAVELIN Renal 101 trial: first-line avelumab plus axitinib versus sunitinib in patients with advanced renal cell carcinoma. Ann Oncol. 202031(8):1030-1039.  Hahn, OM., et. al. (2008). Dynamic contrast-enhanced magnetic resonance imaging pharmacodynamic biomarker study of sorafenib in metastatic renal carcinoma. J Clin Oncol. Oct 1;26(28):4572-8. |
| **Cumulative analysis of results from several studies** | Rini, BI., et al.. (2021). Time to Resolution of Axitinib-Related Adverse Events After Treatment Interruption in Patients With Advanced Renal Cell Carcinoma. Clin Genitourin Cancer. Oct;19(5):e306-e312.  Vano, YA.,et al. (2022). Nivolumab, nivolumab-ipilimumab, and VEGFR-tyrosine kinase inhibitors as first-line treatment for metastatic clear-cell renal cell carcinoma (BIONIKK): a biomarker-driven, open-label, non-comparative, randomised, phase 2 trial. Lancet Oncol. May;23(5):612-624. |
| **Inappropriate language** | Rexer, H., et al. (2020). A Phase 2, Randomized, Open-Label Study of Nivolumab Combined with Ipilimumab Versus Sunitinib Monotherapy in Subjects with Previously Untreated and Advanced (unresectable or metastatic) non-clear Cell Renal Cell Carcinoma - SUNNIFORECAST AN 41/16 der AUO. Aktuelle Urologi, 51(3), 236‐238. |
| **No full text available** | Alba, SO., et al . (2019). Cabozantinib for the treatment of advanced renal cell carcinoma in treatment-naive adults. European journal of clinical pharmacy, 21(3), 160‐163. |

## **Homogeneity analysis and patient’s characteristics.**

**Supplementary Table 6. Baseline characteristic of patients from trials included in network meta-analysis – homogeneity analysis.**

| **Trial** | **Trial arms** | **Diagnosis – type of RCC** | **Clear-cell histologic component** | **Common sites of metastases, %** | | | | **Age, median (range) [years]** | **Sex,**  **% of female/**  **% of male** | **ECOG performance status^, %** | | | **Previous nephrectomy, %** | **Previous radiation therapy, %** | **MSKCC* risk factor, %** | | |
| --- | --- | --- | --- | --- | --- | --- | --- | --- | --- | --- | --- | --- | --- | --- | --- | --- | --- |
|  |  |  |  | **Lung** | **Liver** | **Bone** | **Lymph nodes** |  |  | **0** | **1** | **2** |  |  | **0**  (low, favorable) | **1-2**  (inter-mediate) | **3**  (high/  poor) |
| **NCT00098657, NCT00083889**  **[36]-[39]** | Sunitinib PO at a dose of 50 mg once a day for 4 weeks, followed by 2 weeks without treatment | mRCC | RCC with a clear cell component but no specific percentage of patients | 78 | 26 | 30 | 58 | 62.0  (27-87) | 29/71 | 62.0 | 38.0 | - | 91.0 | 14.0 | 38.0 | 56.0 | 6.0 |
|  | Interferon alpha-2a SC at a dose of 9 MU thrice a week |  |  | 79 | 24 | 30 | 53 | 59.0  (34-85) | 28/72 | 61.0 | 39.0 | - | 89.0 | 14.0 | 34.0 | 59.0 | 7.0 |
| **NCT00117637**  **[40]-[41]** | Sorafenib PO at a dose of 400 mg twice a day | Unresectable and/or metastatic RCC (>80% with metastases) | 87.8% of all randomly assigned patient cases were purely clear cell histology, and 12.2% were predominantly clear cell histology | 86.6 | 24.7 | 32 | 55.7 | 62.0  (34-78) | 33/67 | 57.7 | 42.3 | - | 97.9 | 22.7 | 53.6 | 45.4 | 1.0 |
|  | Interferon alpha-2a SC at a dose of 9 MU thrice a week |  |  | 80.4 | 20.7 | 37 | 46.7 | 62.5  (18-80) | 43.5/56.5 | 53.3 | 46.7 | - | 90.2 | 13.0 | 51.1 | 47.8 | 0.0 |
| **TIVO**  **[42]- [43]** | Sorafenib PO at a dose of 400 mg twice a day | Recurrence or/and mRCC (>70% without previous systemic therapy) | RCC with a clear cell component but no specific percentage of patients | 82 | 26 | 23 | 70 | 59  (23-85) | 26/74 | 54.0 | 46.0 | - | - | - | 34.0 | 62.0 | 4.0 |
|  | Tivozanib PO at a dose of 1.5 mg once a day every day for 3 weeks followed by 1 week off |  |  | 79 | 19 | 20 | 65 | 59  (23-83) | 29/71 | 45.0 | 55.0 | - | - | - | 27.0 | 67.0 | 7.0 |
| **Alliance A031203 CABOSUN**  **[44]-[46]** | Sunitinib PO at a dose of 50 mg once a day for 4 weeks, followed by 2 weeks without treatment | Advanced RCC or mRCC (not amenable to curative surgery or radiotherapy) | RCC with a clear cell component but no specific percentage of patients | - | - | 35.9 | - | 64  (31-87) | 26.9/73.1 | 46.2 | 41.0 | 12.8 | 76.9 | - | - | 80.8 | 19.2 |
|  | Cabozantinib PO at a dose of 60 mg once a day |  |  |  |  | 36.7 |  | 63  (40-82) | 16.5/83.5 | 45.6 | 41.8 | 12.7 | 72.2 | - | - | 81.0 | 19.0 |
| **COMPARZ**  **[47]-[48]** | Sunitinib PO at a dose of 50 mg once a day for 4 weeks, followed by 2 weeks without treatment | Advanced RCC or mRCC | RCC with a clear cell component but no specific percentage of patients | 77 | 20 | 15 | 45 | 62  (23-86) | 25/75 | 76.0 | 24.0 | - | 84.0 | 8.0 | 27.0 | 59.0 | 9.0 |
|  | Pazopanib PO at a dose of 800 mg once a day |  |  | 76 | 15 | 20 | 40 | 61  (18-88) | 29/71 | 75.0 | 25.0 | - | 82.0 | 8.0 | 27.0 | 58.0 | 12.0 |
| **SWITCH**  **[49]-[50]** | Sorafenib PO at a dose of 400 mg twice a day | Advanced RCC or mRCC | 90.0% | 79 | 20 | 12 | 48 | 64  (39-84) | 24/76 | 66.0 | 31.0 | - | 92.0 | 8.8 | 39.0 | 59.0 | 0.5 |
|  | Sunitinib PO at a dose of 50 mg once a day for 4 weeks, followed by 2 weeks without treatment |  | 84.0% | 72 | 24 | 17 | 40 | 65  (40-83) | 26/74 | 60.0 | 38.0 | 0.6 | 92.0 | 13.0 | 45.0 | 51.0 | 0.5 |
| **SWITCH II**  **[51]- [52]** | Sorafenib PO at a dose of 400 mg twice a day | Advanced RCC or mRCC | 89.0% | 69 | 17 | 20 | - | 68  (31-84) | 28/72 | 68.0 | 32.0 | - | 98.0 | - | 50.0 | 48.0 | 2.0 |
|  | Pazopanib PO at a dose of 800 mg once a day |  | 85.0% | 74 | 20 | 20 | - | 68  (26-86) | 27/73 | 70.0 | 29.0 | - | 99.0 | - | 48.0 | 47.0 | 3.0 |
| **CROSS-J -RCC**  **[53]-[55]** | Sunitinib PO at a dose of 50 mg once a day for 4 weeks, followed by 2 weeks without treatment | mRCC | RCC with a clear cell component but no specific percentage of patients | 70 | 7 | 23 | 33 | 67  (41-79) | 19/81 | - | - | - | 88.3 in both group | - | 21.0 | 78.9 | - |
|  | Sunitinib PO at a dose of 50 mg once a day for 4 weeks, followed by 2 weeks without treatment |  |  | 74 | 10 | 33 | 24 | 66  (44-79) | 16/84 | - | - | - |  | - | 22.0 | 77.8 | - |
| **TemPa**  **[56]** | Temsirolimus IV at a dose of 25 mg twice a week | Advanced RCC or mRCC | RCC with a clear cell component but no specific percentage of patients | Metastatic disease in more than one organ site | | | | 61  (42-80) | 31.4/68.6 | 2.9 | 40.0 | 57.1 | 42.9 | - | - | 31.4 | 68.6 |
|  | Pazopanib PO at a dose of 800 mg once a day |  |  |  |  |  |  | 61  (37-74) | 17.7/82.3 | 2.9 | 35.3 | 61.8 | 44.1 | - | - | 23.5 | 76.5 |
| **CheckMate 9ER**  **[57]- [59]** | Nivolumab IV at a dose of 240 mg once every 2 weeks + cabozantinib PO at a dose of 40 mg once a day | Advanced RCC | RCC with a clear cell component but no specific percentage of patients | 73.7 | 22.6 | 24.1 | 40.2 | 62  (29-90) | 22.9/77.1 | 79.6 | 20.4 | - | 68.7 | 14.2 | 22.9 | 58.2 | 18.9 |
|  | Sunitinib PO at a dose of 50 mg once a day for 4 weeks, followed by 2 weeks without treatment |  |  | 75.9 | 16.2 | 22.0 | 39.9 | 61  (28-86) | 29.3/70.7 | 73.5 | 25.9 | - | 71.0 | 13.7 | 22.0 | 57.3 | 20.7 |
| **KEYNOTE-426**  **[60]- [62]** | Pembrolizumab IV at a dose of 200 mg once every 3 weeks + axitinib PO at a dose of 5 mg twice a day | Recurrent or metastatic RCC (no previous systemic therapy) | RCC with a clear cell component but no specific percentage of patients | 72.2 | 15.3 | 23.8 | 46.1 | 62  (30-89) | 28.7/71.3 | - | - | - | 82.6 | 9.5 | 31.9 | 55.1 | 13.0 |
|  | Sunitinib PO at a dose of 50 mg once a day for 4 weeks, followed by 2 weeks without treatment |  |  | 72.0 | 16.6 | 24.0 | 45.9 | 61  (26-90) | 25.4/74.6 | - | - | - | 83.4 | 9.3 | 30.5 | 57.3 | 12.1 |
| **CLEAR**  **[63]- [64]** | Lenvatinib PO at a dose of 20 mg once a day + pembrolizumab IV at a dose of 200 mg once every 3 weeks | Advanced RCC (finally all with at least one metastatic site) | RCC with a clear cell component but no specific percentage of patients | 70.1 | 16.9 | 23.9 | 47.9 | 64  (34-88) | 28.2/71.8 | 83.1 | 16.9 | - | 73.8 | - | 27.0 | 63.9 | 9.0 |
|  | Lenvatinib PO at a dose of 18 mg once a day + everolimus PO at a dose of 5 mg once a day |  |  | 68.6 | 17.4 | 24.1 | 45.7 | 62  (32-86) | 25.5/74.5 | 80.1 | 19.6 | - | 72.8 | - | 27.5 | 63.6 | 9.0 |
|  | Sunitinib PO at a dose of 50 mg once a day for 4 weeks, followed by 2 weeks without treatment |  |  | 66.9 | 17.1 | 27.2 | 44.5 | 61  (29-82) | 23.0/77.0 | 82.4 | 17.4 | - | 77.0 | - | 27.2 | 63.9 | 9.0 |
| **JAVELIN Renal 101**  **[65]- [67]** | Avelumab IV at a dose of 10 mg per kilogram of body weight every 2 weeks + axitinib PO at a dose of 5 mg twice a day | Advanced or metastatic RCC (finally all with at least one RECIST-defined tumor sites) | RCC with a clear cell component but no specific percentage of patients | - | | | | 62  (29-83) | 28.5/71.5 | - | - | - | 79.6 | - | 21.7 | 64.0 | 11.5 |
|  | Sunitinib PO at a dose of 50 mg once a day for 4 weeks, followed by 2 weeks without treatment |  |  |  |  |  |  | 61  (27-88) | 22.5/77.5 | - | - | - | 80.0 | - | 22.5 | 66.0 | 10.1 |

* for study TemPa, CheckMate 9ER, KEYNOTE-426 the prognostic risk score was assessed using the IMDC scale, where  (0 [favorable] vs. 1 or 2 [intermediate] vs. 3 to 6 [poor]); ^ for the COMPARZ, SWITCH II, CheckMate 9ER and CLEAR study, Karnofsky performance status was shown, which correspond to the ECOG scale: ECOG grade 0 corresponds to "100 - normal, no evidence of disease; 90 - able to carry out normal activities, minor signs or symptoms of disease" on the Karnofsky scale and grade 1 on the ECOG scale corresponds to "80 - normal activity with exertion, some signs or symptoms of disease; 70 - takes care of himself, but is unable to carry out normal activities or perform active work”; PO – per os (orally); SC – subcutaneously; IV – intravenously; RCC – renal-cell carcinoma; mRCC - metastatic renal-cell carcinoma; ECOG - an Eastern Cooperative Oncology Group performance status.

**Supplementary Table 7. Definition of safety outcomes and methods of safety analysis in included trials.**

| **Trial** | **Methods of reporting adverse events** | **Discontinuation due to AE** | **Method of safety analysis** |
| --- | --- | --- | --- |
| **NCT00098657, NCT00083889**  **[36]-[39]** | Treatment-related AE  *Listed are all treatment-related adverse events of interest and those occurring in at least 10% of patients in the sunitinib group*  Adverse events were graded with the use of the Common Terminology Criteria for Adverse Events of the National Cancer Institute, version 3.0. | Discontinuation due to AE | All randomized patients, 15 patients excluded from safety analysis because did not received interferon alfa. |
| **NCT00117637**  **[40]- [41]** | TEAE and drug-related individual AE  *TEAE occurred in ≥ 5% of patients in any treatment group*  *TEAE ≥3* o*ccurred in ≥ 2% of patients in any treatment group*  According to National Cancer Institute Common Terminology Criteria for Adverse Events, version 3.0 | Discontinuation due to TEAE | Patients given one or more doses of sorafenib or IFN-α-2a were evaluated at each visit for safety |
| **TIVO [42]- [43]** | TEAE  *Occurred in ≥ 10% of patients in any treatment group*  TEAE were collected throughout the patients’ participation, including a period of 30 days after the last dose of study drug. AEs were graded according to the National Cancer Institute’s Common Terminology Criteria for Adverse Events version 3.0. | Discontinuation due to TEAE | All randomized patients receiving at least one dose of study drug. |
| **Alliance A031203 CABOSUN [44]- [46]** | AE/TEAE  *All-causality adverse events reported in at least 20% of patients in either study group are shown*  AE severity grades were assessed by the investigator using the National Cancer Institute Common Terminology Criteria for Adverse Events (version 4.0) | Discontinuation due to AE | All randomized patients receiving at least one dose of study drug. |
| **COMPARZ [47]-[48]** | AE/TEAE  *Occurred in ≥10% patients in any treatment group.*  AE were graded according to the Common Terminology Criteria for Adverse Events of the National Cancer Institute, version 3.0. | Discontinuation due to AE | All randomized patients receiving at least one dose of study drug. |
| **SWITCH [49]-[50]** | TEAE  *Any-grade TEAE in >20% of patients in either arm, or grade ¾ TEAEs in >3% of patients in either arm.*  Safety reported according to National Cancer Institute Common Terminology Criteria for Adverse Events v3.0. | AEs leading to withdrawal | All randomized patients receiving at least one dose of study drug. |
| **SWITCH II**  **[51]-[52]** | TEAE  Safety and tolerability according to National Cancer Institute Common Terminology Criteria for Adverse Events v4.03 | Any TEAE leading to treatment stop | Safety and tolerability of the two treatment sequences  were assessed for all patients, having received at least  one dose of study drug |
| **CROSS-J –RCC [53]-[55]** | AE  *All-causality AEs6457 detected in > 40% of patients*  AEs were graded using the National Cancer Institute Common Terminology Criteria for Adverse Events, version 3.0 | Discontinued therapy because of treatment-related AE | All randomized patients receiving at least one dose of study drug. |
| **TemPa [56]** | TEAE  *Occurred with ≥10% incidence during first-line therapy*  TEAEs were graded according to the National Cancer Institute Common Terminology Criteria for Adverse Events v4.0. | Discontinuation due to TEAE | All randomized patients. |
| **CheckMate 9ER**  **[57]-[59]** | AE  *AE of any cause that occurred in at least 10% of patients in either group while patients were receiving the assigned treatment or within 30 days after the end of the trial treatment period.*  Adverse events were graded according to the National Cancer Institute Common Terminology Criteria for Adverse Events, version 4.0 | Discontinuation of a trial drug due to adverse events of any cause | All randomized patients receiving at least one dose of study drug. |
| **KEYNOTE-426**  **[60]-[62]** | AE and separately treatment-related AE  *Occurred in ≥10% patients in any treatment group*  AE graded according to the National Cancer Institute Common Terminology Criteria for Adverse Events, version 4.0 | Treatment-related adverse events led to discontinuation | All randomized patients receiving at least one dose of study drug. |
| **CLEAR [63]-[64]** | AE (the definition suggests TEAE)  *AE of any cause that emerged or worsened during treatment in at least 25% of the patients in any treatment group*  AE were monitored and recorded with the use of the Common Terminology Criteria for Adverse Events, version 4.03 | Discontinuation due to TEAE | All randomized patients receiving at least one dose of study drug. |
| **JAVELIN Renal 101 [65]**-**[67]** | AE (the definition suggests TEAE)  *AE of any grade that occurred during treatment in 10% or More of patients or AEs of grade 3 or higher that occurred in 5% or more of patients*  Adverse events were graded according to the National Cancer Institute Common Terminology Criteria for Adverse Events, version 4.03 | Discontinuation due to AE | All randomized patients receiving at least one dose of study drug. |

AE – adverse events; TEAE – treatment-emergent adverse events.

## **NMA input**

**Supplementary Figure 1. An example network diagram considering all included trials. The therapies of interest are marked in green; the thickness of the lines joining the interventions is related to the number of trials for the comparison. AXI+AVE – axitinib + avelumab, AXI+PEM2 – axitnib + pembrolizumab, CAB – cabozantinib, CAB+NIV – cabozantinib + nivolumab, INF – interferon alfa, LEN+PEM – lenvatinib + pembrolizumab, PAZ – pazopanib, SOR – sorafenib, SUN – sunitinib, TEM – temsirolimus, TIV – tivozanib.**


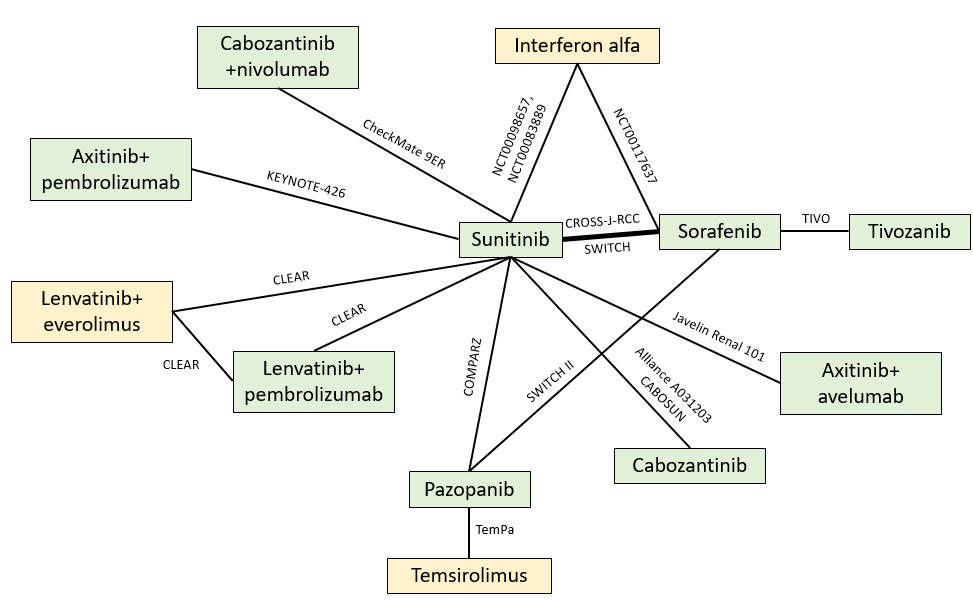


**Supplementary Table 8. The number of trials included in NMA for each endopint.**

| **Endopint** | **Number of trials included in NMA** | **Acronyms of trials included in NMA** |
| --- | --- | --- |
| **Adverse events** | 9 | TIVO; Alliance A031203 CABOSUN; COMPARZ; SWITCH; SWITCH II; CheckMate 9ER; KEYNOTE-426; CLEAR; JAVELIN Renal 101 |
| **Adverse events ≥3 grade** | 11 | NCT00117637; TIVO; Alliance A031203 CABOSUN; COMPARZ; SWITCH; SWITCH II; CROSS-J –RCC; CheckMate 9ER; KEYNOTE-426; CLEAR; JAVELIN Renal 101 |
| **Discontinuation of study drug due to adverse events** | 13 | NCT00098657, NCT00083889; NCT00117637; TIVO; Alliance A031203 CABOSUN; COMPARZ; SWITCH; SWITCH II; CROSS-J –RCC; TemPa; CheckMate 9ER; KEYNOTE-426; CLEAR; JAVELIN Renal 101 |
| **Dosage change due to adverse events** | 10 | NCT00098657, NCT00083889; NCT00117637; TIVO; Alliance A031203 CABOSUN; COMPARZ; SWITCH; SWITCH II; KEYNOTE-426; CLEAR; JAVELIN Renal 101 |
| **Fatigue (grade 3 of more)** | 11 | TIVO; Alliance A031203 CABOSUN; COMPARZ; SWITCH; SWITCH II; CROSS-J-RCC; TemaPa; CheckMate 9ER; KEYNOTE-426; CLEAR; JAVELIN Renal 101 |
| **Diarrhea (grade 3 of more)** | 11 | TIVO; Alliance A031203 CABOSUN; COMPARZ; SWITCH; SWITCH II; CROSS-J-RCC; TemaPa; CheckMate 9ER; KEYNOTE-426; CLEAR; JAVELIN Renal 101 |
| **Nausea (grade 3 of more)** | 11 | TIVO; Alliance A031203 CABOSUN; COMPARZ; SWITCH; SWITCH II; CROSS-J-RCC; TemaPa; CheckMate 9ER; KEYNOTE-426; CLEAR; JAVELIN Renal 101 |
| **Vomiting (grade 3 of more)** | 8 | Alliance A031203 CABOSUN; COMPARZ; CROSS-J-RCC; TemaPa; CheckMate 9ER; KEYNOTE-426; CLEAR; JAVELIN Renal 101 |
| **Hypertension (grade 3 of more)** | 11 | TIVO; Alliance A031203 CABOSUN; COMPARZ; SWITCH; SWITCH II; CROSS-J-RCC; TemaPa; CheckMate 9ER; KEYNOTE-426; CLEAR; JAVELIN Renal 101 |
| **Dysphonia (grade 3 of more)** | 6 | It was impossible to conduct the NMA because of too small numer if trials with reported grade ≥3 of dysphonia |

**Supplementary Table 9. General safety profile.**

| **Trial** | **Comparison** | **AE/TEAE, n/N (%)** | **AE/TEAE grade 3 or more, n/N (%)** | **Discontinuation of study drug due to AE, n/N (%)** | **Dosage modification due to AE, n/N (%)** |
| --- | --- | --- | --- | --- | --- |
| **NCT00098657, NCT00083889**  **[36]-[39]** | Sunitinib PO at a dose of 50 mg once a day for 4 weeks, followed by 2 weeks without treatment | Only treatment related AE reported | | 70/375 (19.0%) | 188/375 (50.0%) |
|  | Interferon alpha SU at a dose of 9 MU thrice a week |  |  | 86/360 (23.0%) | 97/360 (27.0%) |
| **NCT00117637 [40]-[41]** | Sorafenib PO at a dose of 400 mg twice a day | only treatment related AE reported | 68/97 (70.1%) | 11/97 (11.3%) | 32/97 (33.0%) |
|  | Interferon alpha-2a SU at a dose 9 MU thrice a week |  | 49/90 (54.4%) | 14/90 (15.2%) | 24/90 (26.0%) |
| **TIVO [42]-[43]** | Sorafenib PO at a dose of 400 mg twice a day | 249/257 (97.0%) | 179/257 (70.0%) | 18/257 (7.0%) | 111/257 (43.0%) |
|  | Tivozanib PO at a dose of 1.5 mg once a day every day for 3 weeks followed by 1 week off | 235/259 (91.0%) | 159/259 (61.0%) | 19/259 (7.3%) | 37/259 (14.0%) |
| **Alliance A031203 CABOSUN [44]-[46]** | Sunitinib PO at a dose of 50 mg once a day for 4 weeks, followed by 2 weeks without treatment | 72/72 (100%) | 47/72 (65.0%) | 16/72 (20.0%) | 25/72 (35.0%) |
|  | Cabozantinib PO at a dose of 60 mg once a day | 75/78 (96.0%) | 53/78 (68.0%) | 16/78 (21.0%) | 36/78 (46.0%) |
| **COMPARZ [47]-[48]** | Sunitinib PO at a dose of 50 mg once a day for 4 weeks, followed by 2 weeks without treatment | 544/548 (99.3%) | 311/548 (56.8%) [grade 3]  402/548 (73.4%) [grade 3+4] | 112/548 (20.4%) | 279/548 (51.0%) |
|  | Pazopanib PO at a dose of 800 mg once a day | 552/554 (99.6%) | 327/554 (59.0%) [grade 3]  412/554 (74.4%) [grade 3-4] | 135/554 (24.4%) | 244/554 (44.0%) |
| **SWITCH [49]-[50]** | Sorafenib PO at a dose of 400 mg twice a day | 172/177 (97.0%) | 117/177 (66.0%) | 33/177 (19.0%) | 65/177 (37.0%) |
|  | Sunitinib PO at a dose of 50 mg once a day for 4 weeks, followed by 2 weeks without treatment | 172/176 (98.0%) | 118/176 (67.0%) | 52/176 (30.0%) | 65/176 (37.0%) |
| **SWITCH II [51]- [52]** | Sorafenib PO at a dose of 400 mg twice a day | 179/183 (98.0%) | 108/183 (59.0%) | 61/183 (33.0%) | 64/183 (35.0%) |
|  | Pazopanib PO at a dose of 800 mg once a day | 182/183 (99.5%) | 117/183 (64.0%) | 44/183 (24.0%) | 80/183 (44.0%) |
| **CROSS-J –RCC [53]- [55]** | Sunitinib PO at a dose of 50 mg once a day for 4 weeks, followed by 2 weeks without treatment | - | 50/57 (79.4%) | 13/57 (22.8%) | - |
|  | Sorafenib PO at a dose of 400 mg twice a day | - | 48/63 (84.2%) | 12/63 (19.0%) | - |
| **TemPa [56]** | Temsirolimus IV at a dose of 25 mg twice a week | - | - | 3/35 (8.0%) | - |
|  | Pazopanib PO at a dose of 800 mg once a day | - | - | 2/34 (6.0%) | - |
| **CheckMate 9ER [57]-[59]** | Nivolumab IV at a dose of 240 mg once every 2 weeks + cabozantinib PO at a dose of 40 mg once a day | 319/320 (99.7%) | 241/320 (75.3%) | 63/320 (19.7%)  [6.6% discontinued nivolumab only, 7.5% discontinued cabozantinib only, and 5.6% discontinued both nivolumab and cabozantinib] | - |
|  | Sunitinib PO at a dose of 50 mg once a day for 4 weeks, followed by 2 weeks without treatment | 317/320 (99.1%) | 226/320 (70.6%) | 54/320 (16.9%) | - |
| **KEYNOTE-426* [60]-[62]** | Pembrolizumab IV at a dose of 200 mg once every 3 weeks + axitinib PO at a dose of 5 mg twice a day | 422/429 (98.%) | 303/429 (75.8) [grade 3 or more] | 131/429 (30.5%) of either drug  46/429 (10,7%) of both drugs | 87/429 (20.3%) |
|  | Sunitinib PO at a dose of 50 mg once a day for 4 weeks, followed by 2 weeks without treatment | 423/425 (99.5%) | 300/425 (70.6%) [grade 3 or more] | 59/425 (13.9%) | 128/425 (30.1%) |
| **CLEAR [63]-[64]** | Lenvatinib PO at a dose of 20 mg once a day + pembrolizumab IV at a dose of 200 mg once every 3 weeks | 351/352 (99.7%) | 290/352 (82.4%) | 131/352 (37.2%)  [90/352 (25.6%) of lenvatynib; 101/252 (28.7%) of pembrolizumab; 47/352 (13.4%) of both drugs] | 276/352 (78.4%) of both drugs |
|  | Lenvatinib PO at a dose of 18 mg once a day + everolimus PO at a dose of 5 mg once a day | 354/355 (99.7%) | 295/355 (83.1%) | 96/355 (27.0%)  [78/355 (22.0%) of lenvatynib; 88/355 (24.8%) of everolimus; 67/355 (18.9%) of both drugs] | 260/355 (73.2%) of both drugs |
|  | Sunitinib PO at a dose of 50 mg once a day for 4 weeks, followed by 2 weeks without treatment | 335/340 (98.5%) | 244/340 (71.8%) | 49/340 (14.4%) | 171/340 (50.3%) |
| **JAVELIN Renal 101**  **[65]-[67]** | Avelumab IV at a dose of 10 mg per kilogram of body weight every 2 weeks + axitinib PO at a dose of 5 mg twice a day | 432/434 (99.5%) | 309/434 (71.2%) | 33/434 (7.6%) | 183/434 (42.2%) |
|  | Sunitinib PO at a dose of 50 mg once a day for 4 weeks, followed by 2 weeks without treatment | 436/439 (99.3%) | 314/439 (71.5%) | 59/439 (13.4%) | 187/439 (42.6%) |

For study KEYNOTE-426 values given for the first period of treatment; IV – intravenously; SU – subcutaneously; PO – per os (orally).

**Supplementary Table 10. Detailed safety profile – selected adverse events all grades.**

| **Trial** | **Comparison** | **Fatigue** | **Diarrhea** | **Nausea** | **Vomiting** | **Hypertension** | **Dysphonia** |
| --- | --- | --- | --- | --- | --- | --- | --- |
| **NCT00098657, NCT00083889**  **[36]-[39]** | Sunitinib PO at a dose of 50 mg once a day for 4 weeks, followed by 2 weeks without treatment | Only treatment related AE reported | | | | | |
|  | Interferon alpha SU at a dose of 9 MU thrice a week |  |  |  |  |  |  |
| **NCT00117637 [40]-[41]** | Sorafenib PO at a dose of 400 mg twice a day | Only treatment related AE reported | | | | | |
|  | Interferon alpha-2a SU at a dose 9 MU thrice a week |  |  |  |  |  |  |
| **TIVO [42]-[43]** | Sorafenib PO at a dose of 400 mg twice a day | 41/257 (16.0%) | 84/257 (33.0%) | 19/257 (7.0%) | - | 88/257 (34.0%) | 12/257 (5.0%) |
|  | Tivozanib PO at a dose of 1.5 mg once a day every day for 3 weeks followed by 1 week off | 50/259 (19.0%) | 59/259 (23.0%) | 31/259 (12.0%) | - | 115/259 (44.0%) | 55/259 (21.0%) |
| **Alliance A031203 CABOSUN [44]-[46]** | Sunitinib PO at a dose of 50 mg once a day for 4 weeks, followed by 2 weeks without treatment | 49/72 (68.1%) | 39/72 (54.2%) | 28/72 (38.9%) | 16/72 (22,2%) | 32/72 (31.1%) | 2/72 (2.8%) |
|  | Cabozantinib PO at a dose of 60 mg once a day | 50/78 (64.0%) | 57/78 (73.0%) | 25/78 (32.1%) | 18/78 (23.1%) | 52/78 (66.7.%) | 17/78 (21.8%) |
| **COMPARZ [47]-[48]** | Sunitinib PO at a dose of 50 mg once a day for 4 weeks, followed by 2 weeks without treatment | 344/548 (62.8%) | 315/548 (57.5%) | 250/548 (45.6%) | 146/548 (26.6%) | 223/548 (40.7%) | - |
|  | Pazopanib PO at a dose of 800 mg once a day | 302/554 (54.5%) | 348/554 (62.8%) | 247/554 (44.6%) | 155/554 (27.9%) | 257/554 (46.4%) | - |
| **SWITCH [49]-[50]** | Sorafenib PO at a dose of 400 mg twice a day | 56/177 (32.0%) | 96/177 (54.0%) | 39/177 (22.0%) | - | 57/177 (32.0%) | - |
|  | Sunitinib PO at a dose of 50 mg once a day for 4 weeks, followed by 2 weeks without treatment | 70/176 (40.0%) | 70/176 (40.0%) | 53/176 (30.0%) | - | 58/176 (33.0%) | - |
| **SWITCH II [51]- [52]** | Sorafenib PO at a dose of 400 mg twice a day | 68/183 (37.0%) | 102/183 (56.0%) | 45/183 (25.0%) | - | 49/183 (27.0%) | - |
|  | Pazopanib PO at a dose of 800 mg once a day | 82/183 (45.0%) | 109/183 (60.0%) | 66/183 (36.0%) | - | 82/183 (45.0%) | - |
| **CROSS-J –RCC [53]- [55]** | Sunitinib PO at a dose of 50 mg once a day for 4 weeks, followed by 2 weeks without treatment | 33/57 (58.0%) | 14/57 (25.0%) | 19/57 (33.0%) | 13/57 (23.0%) | 32/57 (56.0%) | - |
|  | Sorafenib PO at a dose of 400 mg twice a day | 28/63 (44.0%) | 28/63 (44.0%) | 7/63 (11.0%) | 2/63 (3.0%) | 28/63 (44.0%) | - |
| **TemPa [56]** | Temsirolimus IV at a dose of 25 mg twice a week | 16/35 (46.0%) | 6/35 (17.0%) | 7/35 (20.0%) | 2/35 (6.0%) | 4/35 (11.0%) | - |
|  | Pazopanib PO at a dose of 800 mg once a day | 22/34 (65.0%) | 24/34 (71.0%) | 21/34 (62.0%) | 15/34 (44.0%) | 18/34 (53.0%) | - |
| **CheckMate 9ER [57]-[59]** | Nivolumab IV at a dose of 240 mg once every 2 weeks + cabozantinib PO at a dose of 40 mg once a day | 103/320 (32.2%) | 204/320 (63.8%) | 85/320 (26.6%) | 55/320 (17.2%) | 111/320 (34.7%) | 55/320 (17.2%) |
|  | Sunitinib PO at a dose of 50 mg once a day for 4 weeks, followed by 2 weeks without treatment | 111/320 (34.7%) | 151/320 (47.2%) | 98/320 (30.6%) | 66/320 (20.6%) | 119/320 (37.2%) | 11/320 (3.4%) |
| **KEYNOTE-426* [60]-[62]** | Pembrolizumab IV at a dose of 200 mg once every 3 weeks + axitinib PO at a dose of 5 mg twice a day | 165/429 (38.5%) | 233/429 (54.3%) | 119/429 (27.7%) | 65/429 (15.2%) | 191/429 (44.5%) | 109/429 (25.4%) |
|  | Sunitinib PO at a dose of 50 mg once a day for 4 weeks, followed by 2 weeks without treatment | 161/425 (37.9%) | 191/425 (44.9%) | 134/425 (31.5%) | 79/425 (18.6%) | 193/425 (45.4%) | 14/425 (3.3%) |
| **CLEAR [63]-[64]** | Lenvatinib PO at a dose of 20 mg once a day + pembrolizumab IV at a dose of 200 mg once every 3 weeks | 141/352 (40.1%) | 216/352 (61.4%) | 126/352 (35.8%) | 92/352 (26.1%) | 195/352 (55.4%) | 105/352 (29.8%) |
|  | Lenvatinib PO at a dose of 18 mg once a day + everolimus PO at a dose of 5 mg once a day | 149/355 (42.0%) | 236/355 (66.5%) | 141/355 (39.7%) | 113/355 (31.8%) | 162/355 (45.6%) | 84/355 (23.7%) |
|  | Sunitinib PO at a dose of 50 mg once a day for 4 weeks, followed by 2 weeks without treatment | 125/340 (36.8%) | 168/340 (49.4%) | 113/340 (33.2%) | 68/340 (20.0%) | 141/340 (41.5%) | 14/340 (4.1%) |
| **JAVELIN Renal 101 [65]-[67]** | Avelumab IV at a dose of 10 mg per kilogram of body weight every 2 weeks + axitinib PO at a dose of 5 mg twice a day | 180/434 (41.5%) | 270/434 (62.2%) | 148/434 (34.1%) | 80/434 (18.4%) | 215/434 (49.5%) | 133/434 (30.6%) |
|  | Sunitinib PO at a dose of 50 mg once a day for 4 weeks, followed by 2 weeks without treatment | 176/439 (40.1%) | 209/439 (47.6%) | 172/439 (39.2%) | 87/439 (19.8%) | 158/439 (36.0%) | 14/439 (3.2%) |

* for study KEYNOTE-426 values given for the first period of treatment; IV – intravenously; SU – subcutaneously; PO – per os (orally).

**Supplementary Table 11. Detailed safety profile – selected grade ≥3 adverse events ^.**

| **Trial** | **Comparison** | **Grade ≥3 fatigue** | **Grade ≥3 diarrhea** | **Grade ≥3 nausea** | **Grade ≥3 vomiting** | **Grade ≥3 hypertension** | **Grade ≥3 dysphonia** |
| --- | --- | --- | --- | --- | --- | --- | --- |
| **NCT00098657, NCT00083889**  **[36]-[39]** | Sunitinib PO at a dose of 50 mg once a day for 4 weeks, followed by 2 weeks without treatment | Only treatment related AE reported | | | | | |
|  | Interferon alpha SU at a dose of 9 MU thrice a week |  |  |  |  |  |  |
| **NCT00117637 [40]-[41]** | Sorafenib PO at a dose of 400 mg twice a day | Only treatment related AE reported | | | | | |
|  | Interferon alpha-2a SU at a dose 9 MU thrice a week |  |  |  |  |  |  |
| **TIVO [42]-[43]** | Sorafenib PO at a dose of 400 mg twice a day | 9/257 (4.0%)  [grade 3] | 17/257 (7.0%) [grade 3] | 1/257 (0.4%) [grade 3] | - | 45/257 (18.0%) [grade 3]  46/257 (18.0%) [grade 3+4] | -0/257 (0%) |
|  | Tivozanib PO at a dose of 1.5 mg once a day every day for 3 weeks followed by 1 week off | 14/259 (5.0%)  [grade 3] | 6/259 (2.0%)  [grade 3] | 1/259 (0.4%) [grade 3] | - | 66/259 (25.0%) [grade 3]  [70/259 (27.0%) [grade 3+4] | 0/259 (0%) |
| **Alliance A031203 CABOSUN [44]-[46]** | Sunitinib PO at a dose of 50 mg once a day for 4 weeks, followed by 2 weeks without treatment | 12/72 (16.7%) | 8/72 (11.1%) | 3/72 (4.2%) | 2/72 (2.8%) | 14/72 (19.4%) [grade 3]  15/72 (20.8%)  [grade 3+4] | 1/72 (1.4%) |
|  | Cabozantinib PO at a dose of 60 mg once a day | 5/78 (6.4%) | 8/78 (10.3%) | 2/78 (2.6%) | 1/78 (1.3%) | 22/78 (28.2%) | 1/78 (1.3%) |
| **COMPARZ [47]-[48]** | Sunitinib PO at a dose of 50 mg once a day for 4 weeks, followed by 2 weeks without treatment | 92/548 (16.8%) [grade 3]  94/548 (17.2) [grade 3+4] | 39/548 (7.1%) [grade 3]  42/548 (7.7%) [grade 3+4] | 12/548 (2.2%) | 16/548 (2.9%) | 80/548 (14.6%) [grade 3]  81/548 (14.8%) [grade 3+4] | - |
|  | Pazopanib PO at a dose of 800 mg once a day | 58/554 (10.5%) [grade 3]  59/554 (10.6%) [grade 3+4] | 49/554 (8.8%) | 12/554 (2.2%) | 11/554 (1.9%) | 81/554 (14.6%) [grade 3]  82/554 (14.8%) [grade 3+4] | - |
| **SWITCH [49]-[50]** | Sorafenib PO at a dose of 400 mg twice a day | 8/177 (4.5%) | 9/177 (5.1%) | 2/177 (1.1%) | - | 16/177 (9.0%) | - |
|  | Sunitinib PO at a dose of 50 mg once a day for 4 weeks, followed by 2 weeks without treatment | 13/176 (7.4%) | 5/76 (2.8%) | 3/176 (1.7%) | - | 21/176 (12.0%) | - |
| **SWITCH II [51]- [52]** | Sorafenib PO at a dose of 400 mg twice a day | 3/183 (2.0%) | 11/183 (6.0%) | 5/183 (3.0%) | - | 17/183 (9.0%) | - |
|  | Pazopanib PO at a dose of 800 mg once a day | 3/183 (2.0%) | 6/183 (3.0%) | 1/183 (1.0%) | - | 40/183 (22.0%) | - |
| **CROSS-J –RCC**  **[53]- [55]** | Sunitinib PO at a dose of 50 mg once a day for 4 weeks, followed by 2 weeks without treatment | 9/57 (16.0%) | 0/57 (0.0%) | 3/57 (5.0%) | 1/57 (2.0%) | 10/57 (18.0%) | - |
|  | Sorafenib PO at a dose of 400 mg twice a day | 1/63 (2.0%) | 4/63 (6.0%) | 0/63 (0.0%) | 0/63 (0.0%) | 12/63 (19.0%) | - |
| **TemPa [56]** | Temsirolimus IV at a dose of 25 mg twice a week | 6/35 (17.0%) | 0/35 (0.0%) | 0/35 (0.0%) | 0/35 (0.0%) | 2/35 (6.0%) | - |
|  | Pazopanib PO at a dose of 800 mg once a day | 7/34 (21.0%) | 1/34 (3.0%) | 3/34 (9.0%) | 2/34 (6.0%) | 10/34 (29.0%) | - |
| **CheckMate 9ER [57]-[59]** | Nivolumab IV at a dose of 240 mg once every 2 weeks + cabozantinib PO at a dose of 40 mg once a day | 11/320 (3.4%) | 22/320 (6.9%) | 2/320 (0.6%) | 6/320 (1.9%) | 40/320 (12.5%) | 1/320 (0.3%) |
|  | Sunitinib PO at a dose of 50 mg once a day for 4 weeks, followed by 2 weeks without treatment | 15/320 (4.7%) | 14/320 (4.4%) | 1/320 (0.3%) | 1/320 (0.3%) | 42/320 (13.1%) | 0/320 (0.0%) |
| **KEYNOTE-426* [60]-[62]** | Pembrolizumab IV at a dose of 200 mg once every 3 weeks + axitinib PO at a dose of 5 mg twice a day | 12/429 (2.8%) | 39/429 (9.1%) | 4/429 (0.9%) | 1/429 (0.2%) | 95/429 (22.1%) | 1/429 (0.2%) |
|  | Sunitinib PO at a dose of 50 mg once a day for 4 weeks, followed by 2 weeks without treatment | 28/425 (6.6%) | 20/425 (4.7%) | 4/425 (0.9%) | 4/425 (0.9%) | 82/425 (19.3%) | 0/425 (0.0%) |
| **CLEAR [63]-[64]** | Lenvatinib PO at a dose of 20 mg once a day + pembrolizumab IV at a dose of 200 mg once every 3 weeks | 15/352 (4.3%) | 34/352 (9.7%) | 9/352 (2,6%) | 12/352 (3.4%) | 97/352 (27.6%) | 0/352 (0%) |
|  | Lenvatinib PO at a dose of 18 mg once a day + everolimus PO at a dose of 5 mg once a day | 27/355 (7.6%) | 41/355 (11.5%) | 9/355 (2,5%) | 10/355 (2.8%) | 80/355 (22.5%) | 2/355 (0.6%) |
|  | Sunitinib PO at a dose of 50 mg once a day for 4 weeks, followed by 2 weeks without treatment | 15/340 (4.4%) | 18/340 (5.3%) | 2/340 (0,6%) | 5/340 (1.5%) | 64/340 (18.8%) | 0/340 (0%) |
| **JAVELIN Renal 101 [65]-[67]** | Avelumab IV at a dose of 10 mg per kilogram of body weight every 2 weeks + axitinib PO at a dose of 5 mg twice a day | 15/434 (3.5%) | 29/434 (6.7%) | 6/434 (1.4%) | 4/434 (0.9%) | 111/434 (25.6%) | 2/434 (0.5%) |
|  | Sunitinib PO at a dose of 50 mg once a day for 4 weeks, followed by 2 weeks without treatment | 16/439 (3.6%) | 12/439 (2.7%) | 7/439 (1.6%) | 7/439 (1.6%) | 75/439 (17.1%) | 0/439 (0%) |

* for study KEYNOTE-426 values given for the first period of treatment; ^ in studies where grade 3 and 4 TAEA results were reported separately and each patient was included only in the highest severity AEs observed, we decided to add up the reported grade 3, 4 and 5 AEs; IV – intravenously; SU – subcutaneously; PO – per os (orally).

## **Results of NMA (all TKIs, all comparisons).**


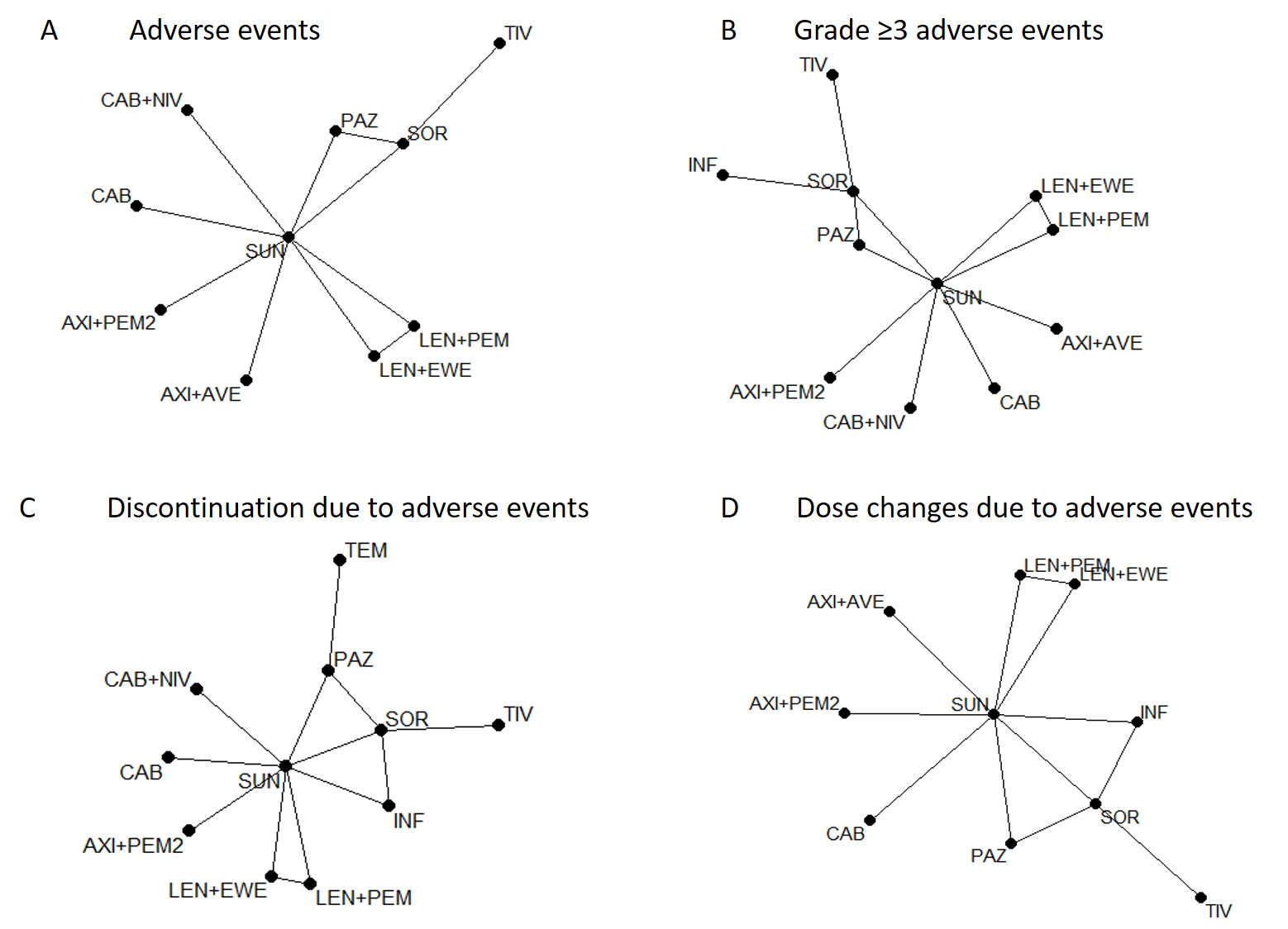


**Supplementary Figure 2. Network diagrams for A – adverse events; B - grade ≥3 of adverse events, C – discontinuation due to adverse events, D – dose modifications due to adverse events. AXI+AVE – axitinib + avelumab, AXI+PEM2 – axitnib + pembrolizumab, CAB – cabozantinib, CAB+NIV – cabozantinib + nivolumab, INF – interferon alfa, LEN+EWE – lenvatinib + everolimus, LEN+PEM – lenvatinib + pembrolizumab, PAZ – pazopanib, SOR – sorafenib, SUN – sunitinib, TEM – temsirolimus, TIV – tivozanib.**


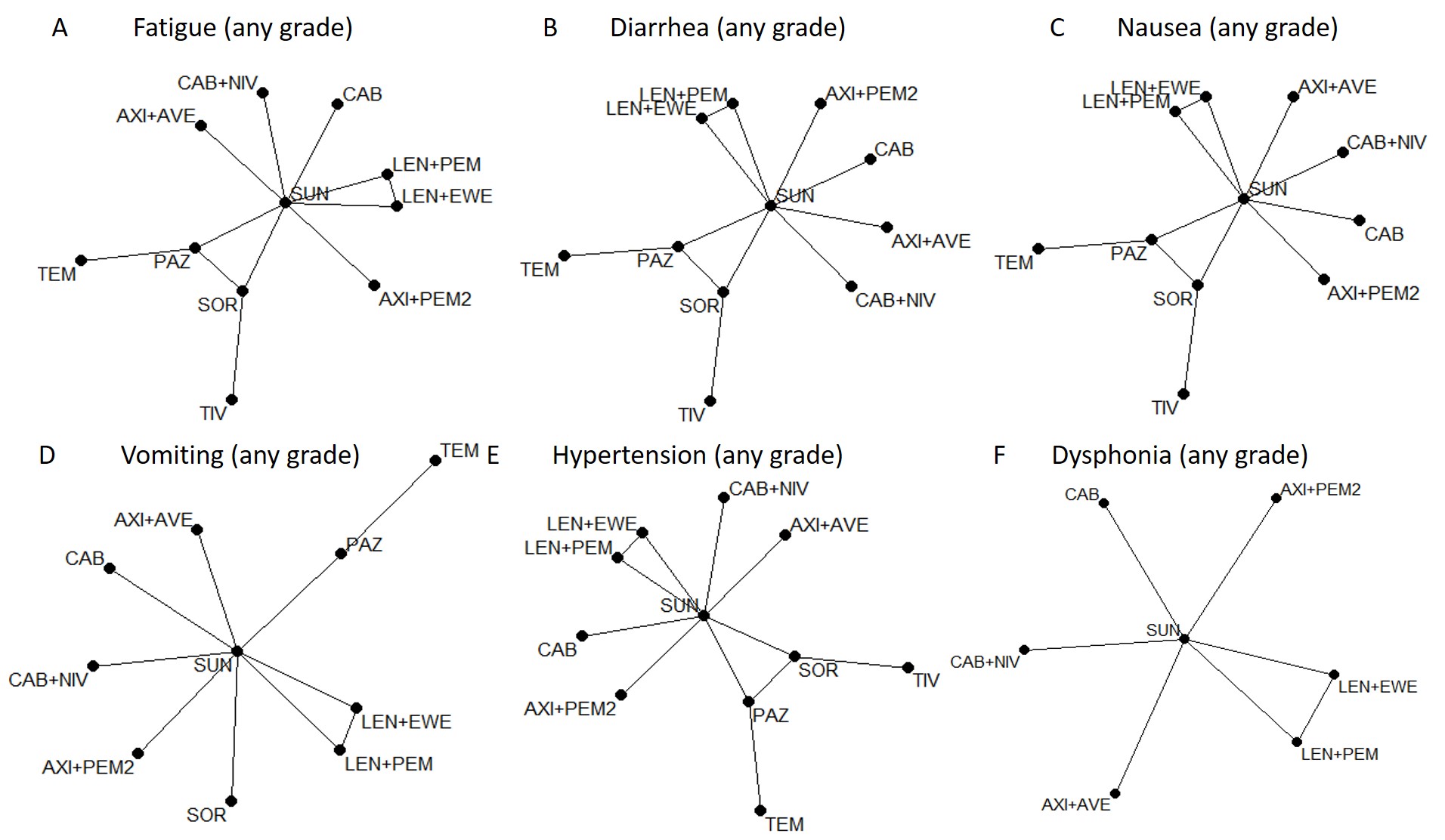


**Supplementary Figure 3. Network diagrams for any grade A – fatigue, B – diarrhea, C – nausea, D – vomiting, E – hypertension, F - dysphonia. AXI+AVE – axitinib + avelumab, AXI+PEM2 – axitnib + pembrolizumab, CAB – cabozantinib, CAB+NIV – cabozantinib + nivolumab, INF – interferon alfa, LEN+EWE – lenvatinib+everolimus, LEN+PEM – lenvatinib + pembrolizumab, PAZ – pazopanib, SOR – sorafenib, SUN – sunitinib, TEM – temsirolimus, TIV – tivozanib.**


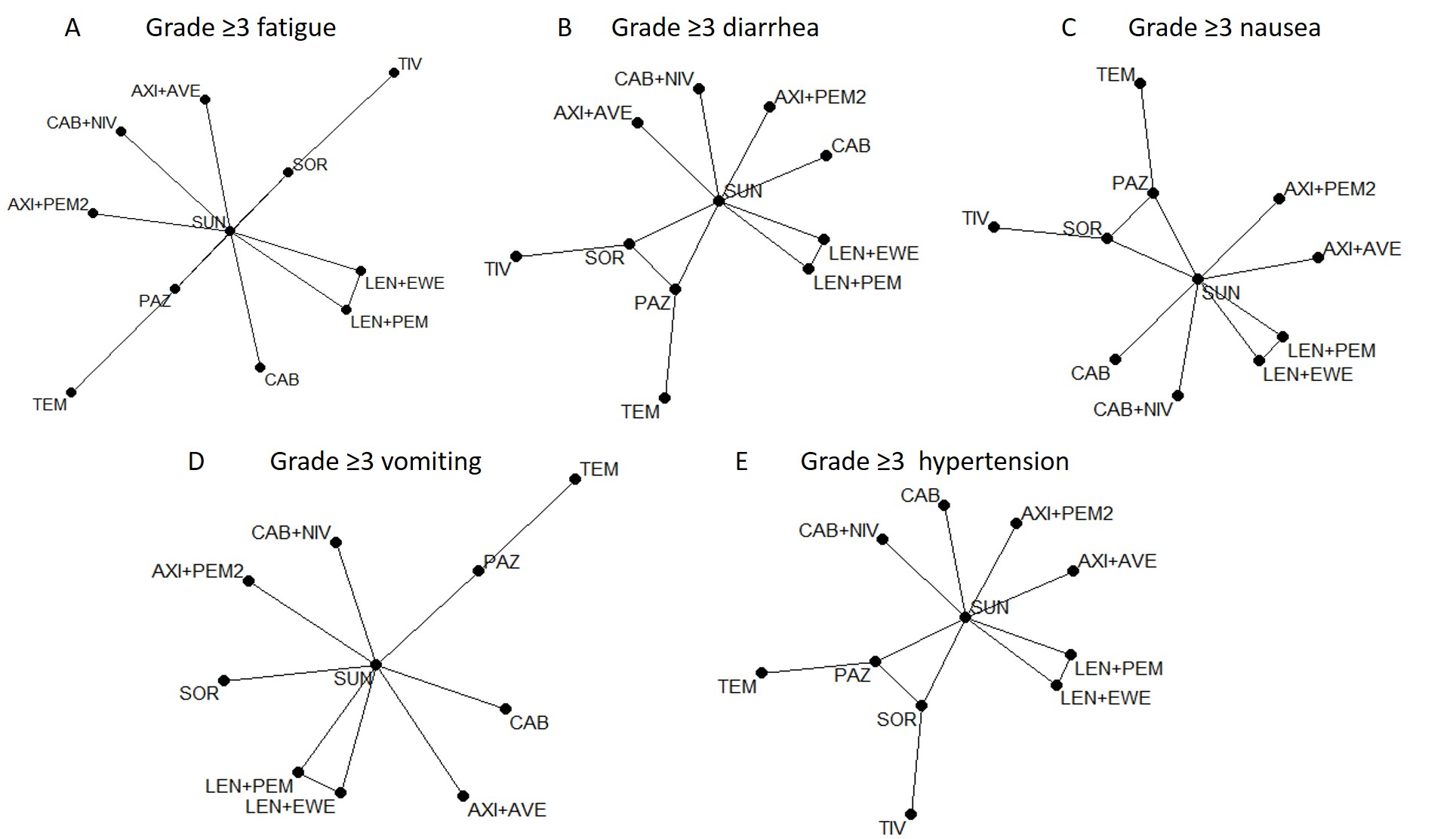


**Supplementary Figure 4. Network diagrams for grade ≥3: A – fatigue, B – diarrhea, C – nausea, D – vomiting, E – hypertension, F - dysphonia. AXI+AVE – axitinib + avelumab, AXI+PEM2 – axitnib + pembrolizumab, CAB – cabozantinib, CAB+NIV – cabozantinib + nivolumab, INF – interferon alfa, LEN+EWE – lenvatinib +everolimus, LEN+PEM – lenvatinib + pembrolizumab, PAZ – pazopanib, SOR – sorafenib, SUN – sunitinib, TEM – temsirolimus, TIV – tivozanib.**

**Supplementary Table 12. P score (overall rank based on P score among all treatment options assessed in clinical trials) for assessed endpoints.**

|  | **Adverse events** | **Grade 3+ adverse events** | **Discontinuation due to adverse events** | **Dose change due to adverse events** | **Fatigue** | **Diarrhea** | **Nausea** | **Vomiting** | **Hypertension** | **Dysphonia** | **Fatigue grade 3+** | **Diarrhea grade 3+** | **Nausea grade 3+** | **Vomiting grade 3+** | **Hypertension grade 3+** |
| --- | --- | --- | --- | --- | --- | --- | --- | --- | --- | --- | --- | --- | --- | --- | --- |
| **CAB** | 2 | 8 | 3 | 8 | 5 | 11 | 3 | 7 | 11 | 4 | 1 | 4 | 3 | 4 | 9 |
| **PAZ** | 7 | 7 | 2 | 5 | 3 | 5 | 8 | 8 | 8 | n/a | 5 | 5 | 2 | 6 | 8 |
| **SOR** | 4 | 3 | 1 | 4 | 2 | 7 | 2 | 2 | 2 | n/a | 2 | 9 | 4 | 3 | 2 |
| **SUN** | 5 | 5 | 5 | 7 | 7 | 2 | 9 | 6 | 5 | 1 | 10 | 3 | 8 | 7 | 4 |
| **TIV** | 1 | 2 | 4 | 1 | 4 | 3 | 7 | n/a | 6 | n/a | 6 | 1 | 5 | n/a | 5 |
| **INF** | n/a | 1 | 8 | 2 | n/a | n/a | n/a | n/a | n/a | n/a | n/a | n/a | n/a | n/a | n/a |
| **TEM** | n/a | n/a | 7 | n/a | 1 | 1 | 1 | 1 | 1 | n/a | 4 | 2 | 1 | 1 | 1 |
| **CAB+**  **NIV** | 8 | 9 | 6 | n/a | 6 | 9 | 5 | 4 | 3 | 2 | 7 | 6 | 9 | 10 | 3 |
| **LEN+**  **PEM** | 9 | 10 | 11 | 10 | 10 | 6 | 10 | 9 | 10 | 6 | 8 | 7 | 11 | 9 | 10 |
| **AXI+**  **AVE** | 6 | 4 | n/a | 6 | 9 | 8 | 4 | 5 | 9 | 7 | 9 | 11 | 6 | 5 | 11 |
| **AXI+**  **PEM2** | 3 | 6 | 10 | 3 | 8 | 4 | 6 | 3 | 4 | 5 | 3 | 8 | 7 | 2 | 6 |
| **LEN+**  **EWE** | 10 | 11 | 9 | 9 | 11 | 10 | 11 | 10 | 7 | 3 | 11 | 10 | 10 | 8 | 7 |

CAB – cabozantinib, PAZ – pazopanib, SOR – sorafenib, SUN – sunitinib, TIV – tivozanib, INF – interferon alfa, TEM – temsirolimus, CAB+NIV – cabozantinib+nivolumab, LEN+PEM – lenvatinib+pembrolizumab, AXI+AVE – axitinib+avelumab, LEN+EWE – lenvatinib+everolimus, AXI+PEM2 – axitnib+pembrolizumab, n/a – not assessable.

**Supplementary Table 13. Results of a comparative analysis of all TKIs and other interventions assessed in included trials, in terms of adverse events presented as ORs with 95% CIs (upper table, the results of direct comparisons are presented above the abbreviations of TKIs) and p-values (bottom table).**

| **TIV** | . | . | 0.31 (0.14 - 0.71) | . | . | . | . | . | . |
| --- | --- | --- | --- | --- | --- | --- | --- | --- | --- |
| 1.55 (0.06 - 42.70) | **CAB** | . | . | 0.15 (0.01 - 2.93) | . | . | . | . | . |
| 0.81 (0.09 - 6.91) | 0.52 (0.02 - 15.21) | **AXI+PEM2** | . | 0.29 (0.06 - 1.38) | . | . | . | . | . |
| 0.31 (0.14 - 0.71) | 0.20 (0.01 - 5.05) | 0.39 (0.05 - 2.83) | **SOR** | 0.80 (0.21 - 3.03) | . | 0.25 (0.03 - 2.22) | . | . | . |
| 0.23 (0.05 - 0.99) | 0.15 (0.01 - 2.93) | 0.29 (0.06 - 1.38) | 0.73 (0.22 - 2.44) | **SUN** | 0.67 (0.11 - 4.05) | 0.49 (0.09 - 2.70) | 0.33 (0.03 - 3.20) | 0.19 (0.02 - 1.64) | 0.19 (0.02 - 1.63) |
| 0.16 (0.02 - 1.56) | 0.10 (0.00 - 3.25) | 0.19 (0.02 - 2.09) | 0.49 (0.06 - 4.27) | 0.67 (0.11 - 4.05) | **AXI+AVE** | . | . | . | . |
| 0.10 (0.02 - 0.56) | 0.06 (0.00 - 1.72) | 0.12 (0.01 - 1.02) | 0.31 (0.07 - 1.46) | 0.43 (0.10 - 1.76) | 0.63 (0.06 - 6.25) | **PAZ** | . | . | . |
| 0.08 (0.01 - 1.13) | 0.05 (0.00 - 2.09) | 0.09 (0.01 - 1.50) | 0.24 (0.02 - 3.16) | 0.33 (0.03 - 3.20) | 0.49 (0.03 - 8.88) | 0.78 (0.05 - 11.27) | **CAB+NIV** | . | . |
| 0.04 (0.00 - 0.59) | 0.03 (0.00 - 1.12) | 0.05 (0.00 - 0.78) | 0.14 (0.01 - 1.64) | 0.19 (0.02 - 1.64) | 0.28 (0.02 - 4.67) | 0.45 (0.03 - 5.89) | 0.58 (0.03 - 13.14) | **LEN+PEM** | 0.99 (0.06 - 15.91) |
| 0.04 (0.00 - 0.59) | 0.03 (0.00 - 1.11) | 0.05 (0.00 - 0.78) | 0.14 (0.01 - 1.63) | 0.19 (0.02 - 1.63) | 0.28 (0.02 - 4.63) | 0.44 (0.03 - 5.84) | 0.57 (0.03 - 13.03) | 0.99 (0.06 - 15.91) | **LEN+EWE** |

| **TIV** |  |  |  |  |  |  |  |  |  |
| --- | --- | --- | --- | --- | --- | --- | --- | --- | --- |
| 0.796 | **CAB** |  |  |  |  |  |  |  |  |
| 0.846 | 0.705 | **AXI+PEM2** |  |  |  |  |  |  |  |
| 0.006 | 0.331 | 0.351 | **SOR** |  |  |  |  |  |  |
| 0.048 | 0.210 | 0.119 | 0.612 | **SUN** |  |  |  |  |  |
| 0.114 | 0.195 | 0.175 | 0.521 | 0.665 | **AXI+AVE** |  |  |  |  |
| 0.009 | 0.102 | 0.052 | 0.139 | 0.240 | 0.697 | **PAZ** |  |  |  |
| 0.061 | 0.115 | 0.094 | 0.280 | 0.340 | 0.631 | 0.853 | **CAB+NIV** |  |  |
| 0.018 | 0.058 | 0.032 | 0.118 | 0.132 | 0.378 | 0.541 | 0.730 | **LEN+PEM** |  |
| 0.018 | 0.057 | 0.032 | 0.116 | 0.130 | 0.375 | 0.536 | 0.726 | 0.995 | **LEN+EWE** |

**Supplementary Table 14. Results of a comparative analysis of all TKIs and other interventions assessed in included trials in terms of grade ≥3 adverse events presented as ORs with 95% CIs (upper table, the results of direct comparisons are presented above the abbreviations of TKIs) and p-values (bottom table).**

| **INF** | . | 0.51 (0.28 - 0.93) | . | . | . | . | . | . | . | . |
| --- | --- | --- | --- | --- | --- | --- | --- | --- | --- | --- |
| 0.74 (0.36 - 1.49) | **TIV** | 0.69 (0.48 - 1.00) | . | . | . | . | . | . | . | . |
| 0.51 (0.28 - 0.93) | 0.69 (0.48 - 1.00) | **SOR** | . | 0.84 (0.56 - 1.26) | . | 0.81 (0.53 - 1.24) | . | . | . | . |
| 0.44 (0.21 - 0.92) | 0.60 (0.34 - 1.05) | 0.86 (0.56 - 1.32) | **AXI+AVE** | 0.98 (0.73 - 1.32) | . | . | . | . | . | . |
| 0.43 (0.22 - 0.85) | 0.59 (0.36 - 0.95) | 0.85 (0.62 - 1.16) | 0.98 (0.73 - 1.32) | **SUN** | 1.00 (0.74 - 1.34) | 0.95 (0.73 - 1.24) | 0.89 (0.45 - 1.75) | 0.79 (0.56 - 1.12) | 0.54 (0.38 - 0.78) | 0.52 (0.36 - 0.74) |
| 0.43 (0.21 - 0.90) | 0.59 (0.33 - 1.03) | 0.85 (0.55 - 1.30) | 0.98 (0.65 - 1.49) | 1.00 (0.74 - 1.34) | **AXI+PEM2** | . | . | . | . | . |
| 0.41 (0.21 - 0.81) | 0.56 (0.34 - 0.91) | 0.81 (0.59 - 1.11) | 0.94 (0.64 - 1.37) | 0.95 (0.75 - 1.21) | 0.95 (0.65 - 1.40) | **PAZ** | . | . | . | . |
| 0.38 (0.15 - 1.00) | 0.52 (0.23 - 1.20) | 0.75 (0.36 - 1.59) | 0.87 (0.42 - 1.83) | 0.89 (0.45 - 1.75) | 0.89 (0.42 - 1.86) | 0.93 (0.45 - 1.92) | **CAB** | . | . | . |
| 0.34 (0.16 - 0.73) | 0.46 (0.26 - 0.84) | 0.67 (0.42 - 1.07) | 0.78 (0.49 - 1.22) | 0.79 (0.56 - 1.12) | 0.79 (0.50 - 1.25) | 0.83 (0.54 - 1.27) | 0.89 (0.41 - 1.91) | **CAB+NIV** | . | . |
| 0.23 (0.11 - 0.51) | 0.32 (0.17 - 0.58) | 0.46 (0.29 - 0.74) | 0.53 (0.34 - 0.85) | 0.54 (0.38 - 0.78) | 0.54 (0.34 - 0.87) | 0.57 (0.37 - 0.88) | 0.61 (0.28 - 1.32) | 0.69 (0.42 - 1.14) | **LEN+PEM** | 0.95 (0.64 - 1.41) |
| 0.22 (0.10 - 0.48) | 0.30 (0.17 - 0.56) | 0.44 (0.27 - 0.71) | 0.51 (0.32 - 0.81) | 0.52 (0.36 - 0.74) | 0.52 (0.32 - 0.83) | 0.54 (0.35 - 0.84) | 0.58 (0.27 - 1.26) | 0.66 (0.40 - 1.09) | 0.95 (0.64 - 1.41) | **LEN+EWE** |

| **INF** |  |  |  |  |  |  |  |  |  |  |
| --- | --- | --- | --- | --- | --- | --- | --- | --- | --- | --- |
| 0.392 | **TIV** |  |  |  |  |  |  |  |  |  |
| 0.028 | - | **SOR** |  |  |  |  |  |  |  |  |
| 0.029 | 0.073 | 0.497 | **AXI+AVE** |  |  |  |  |  |  |  |
| 0.015 | 0.030 | 0.303 | 0.915 | **SUN** |  |  |  |  |  |  |
| 0.026 | 0.064 | 0.447 | 0.932 | 0.989 | **AXI+PEM2** |  |  |  |  |  |
| 0.010 | 0.019 | 0.186 | 0.736 | 0.690 | 0.807 | **PAZ** |  |  |  |  |
| 0.050 | 0,125 | 0.455 | 0.718 | 0.729 | 0.754 | 0.848 | **CAB** |  |  |  |
| 0.006 | 0.011 | 0.093 | 0.275 | 0.182 | 0.312 | 0.387 | 0.762 | **CAB+NIV** |  |  |
| 0.000 | 0.000 | 0.002 | 0.008 | 0.001 | 0.011 | 0.012 | 0.212 | 0.148 | **LEN+PEM** |  |
| 0.000 | 0.000 | 0.001 | 0.005 | 0.000 | 0.006 | 0.006 | 0.170 | 0.102 | 0.802 | **LEN+EWE** |

**Supplementary Table 15. Results of a comparative analysis of all TKIs and other interventions assessed in included trials in terms of discontinuation of study drug(s) due to adverse events presented as ORs with 95% CIs (upper table, the results of direct comparisons are presented above the abbreviations of TKIs) and p-values (bottom table).**

| **SOR** | 1.58 (0.55 - 4.52) | . | 0.95 (0.30 - 3.03) | 0.64 (0.28 - 1.45) | . | . | 0.69 (0.19 - 2.47) | . | . | . |
| --- | --- | --- | --- | --- | --- | --- | --- | --- | --- | --- |
| 1.01 (0.46 - 2.25) | **PAZ** | . | . | 1.25 (0.47 - 3.37) | . | 0.67 (0.08 - 5.35) | . | . | . | . |
| 0.95 (0.24 - 3.82) | 0.94 (0.22 - 4.02) | **CAB** | . | 0.90 (0.26 - 3.08) | . | . | . | . | . | . |
| 0.95 (0.30 - 3.03) | 0.94 (0.23 - 3.84) | 1.00 (0.16 - 6.12) | **TIV** | . | . | . | . | . | . | . |
| 0.86 (0.45 - 1.65) | 0.85 (0.39 - 1.85) | 0.90 (0.26 - 3.08) | 0.90 (0.24 - 3.41) | **SUN** | 0.83 (0.30 - 2.31) | . | 0.73 (0.27 - 2.01) | 0.45 (0.16 - 1.26) | 0.37 (0.13 - 1.00) | 0.28 (0.10 - 0.78) |
| 0.71 (0.21 - 2.40) | 0.70 (0.19 - 2.55) | 0.75 (0.15 - 3.71) | 0.75 (0.14 - 4.02) | 0.83 (0.30 - 2.31) | **CAB+NIV** | . | . | . | . | . |
| 0.68 (0.07 - 6.28) | 0.67 (0.08 - 5.35) | 0.71 (0.06 - 9.01) | 0.71 (0.06 - 8.77) | 0.79 (0.09 - 7.28) | 0.95 (0.08 - 11.01) | **TEM** | . | . | . | . |
| 0.65 (0.27 - 1.58) | 0.64 (0.22 - 1.88) | 0.69 (0.16 - 3.02) | 0.69 (0.16 - 2.95) | 0.76 (0.33 - 1.74) | 0.92 (0.24 - 3.44) | 0.97 (0.09 - 10.05) | **INF** | . | . | . |
| 0.39 (0.12 - 1.31) | 0.38 (0.11 - 1.39) | 0.41 (0.08 - 2.02) | 0.41 (0.08 - 2.19) | 0.45 (0.16 - 1.26) | 0.55 (0.13 - 2.33) | 0.58 (0.05 - 6.67) | 0.60 (0.16 - 2.23) | **LEN+EWE** | . | 0.63 (0.23 - 1.70) |
| 0.31 (0.09 - 1.05) | 0.31 (0.09 - 1.11) | 0.33 (0.07 - 1.62) | 0.33 (0.06 - 1.75) | 0.37 (0.13 - 1.00) | 0.44 (0.11 - 1.87) | 0.47 (0.04 - 5.35) | 0.48 (0.13 - 1.78) | 0.81 (0.19 - 3.38) | **AXI+PEM2** | . |
| 0.24 (0.07 - 0.82) | 0.24 (0.07 - 0.87) | 0.26 (0.05 - 1.26) | 0.26 (0.05 - 1.37) | 0.28 (0.10 - 0.78) | 0.34 (0.08 - 1.46) | 0.36 (0.03 - 4.17) | 0.37 (0.10 - 1.39) | 0.63 (0.23 - 1.70) | 0.77 (0.19 - 3.24) | **LEN+PEM** |

| **SOR** |  |  |  |  |  |  |  |  |  |  |
| --- | --- | --- | --- | --- | --- | --- | --- | --- | --- | --- |
| 0.974 | **PAZ** |  |  |  |  |  |  |  |  |  |
| 0.943 | 0.931 | **CAB** |  |  |  |  |  |  |  |  |
| 0.933 | 0.930 | 0.999 | **TIV** |  |  |  |  |  |  |  |
| 0.648 | 0.678 | 0.871 | 0.880 | **SUN** |  |  |  |  |  |  |
| 0.583 | 0.591 | 0.722 | 0.734 | 0.719 | **CAB+NIV** |  |  |  |  |  |
| 0.730 | 0.703 | 0.792 | 0.789 | 0.833 | 0.967 | **TEM** |  |  |  |  |
| 0.346 | 0.421 | 0.619 | 0.613 | 0.518 | 0.899 | 0.977 | **INF** |  |  |  |
| 0.128 | 0.146 | 0.274 | 0.297 | 0.130 | 0.416 | 0.660 | 0.443 | **LEN+EWE** |  |  |
| 0.059 | 0.072 | 0.172 | 0.194 | 0.051 | 0.267 | 0.540 | 0.273 | 0.770 | **AXI+PEM2** |  |
| 0.022 | 0.030 | 0.094 | 0.111 | 0.015 | 0.147 | 0.414 | 0.141 | 0.357 | 0.726 | **LEN+PEM** |

**Supplementary Table 16. Results of a comparative analysis of all TKIs and other interventions assessed in included trials in terms of dose modifications due to adverse events as ORs with 95% CIs (upper table, the results of direct comparisons are presented above the abbreviations of TKIs) and p-values (bottom table).**

| **TIV** | . | . | 0.22 (0.11 - 0.42) | . | . | . | . | . | . |
| --- | --- | --- | --- | --- | --- | --- | --- | --- | --- |
| 0.37 (0.16 - 0.88) | **INF** | . | 0.74 (0.33 - 1.64) | . | . | 0.37 (0.21 - 0.65) | . | . | . |
| 0.26 (0.10 - 0.70) | 0.70 (0.33 - 1.50) | **AXI+PEM2** | . | . | . | 0.59 (0.33 - 1.05) | . | . | . |
| 0.22 (0.11 - 0.42) | 0.58 (0.34 - 1.02) | 0.83 (0.40 - 1.74) | **SOR** | 0.69 (0.36 - 1.32) | . | 0.99 (0.52 - 1.90) | . | . | . |
| 0.18 (0.08 - 0.41) | 0.48 (0.26 - 0.90) | 0.69 (0.33 - 1.43) | 0.83 (0.50 - 1.35) | **PAZ** | . | 0.76 (0.44 - 1.30) | . | . | . |
| 0.16 (0.06 - 0.42) | 0.42 (0.20 - 0.89) | 0.60 (0.27 - 1.34) | 0.72 (0.35 - 1.48) | 0.87 (0.43 - 1.79) | **AXI+AVE** | 0.98 (0.56 - 1.71) | . | . | . |
| 0.16 (0.07 - 0.34) | 0.41 (0.25 - 0.68) | 0.59 (0.33 - 1.05) | 0.71 (0.45 - 1.12) | 0.86 (0.55 - 1.35) | 0.98 (0.56 - 1.71) | **SUN** | 0.62 (0.27 - 1.41) | 0.37 (0.21 - 0.66) | 0.28 (0.15 - 0.50) |
| 0.10 (0.03 - 0.30) | 0.26 (0.10 - 0.67) | 0.37 (0.13 - 1.00) | 0.44 (0.17 - 1.12) | 0.53 (0.21 - 1.36) | 0.61 (0.23 - 1.64) | 0.62 (0.27 - 1.41) | **CAB** | . | . |
| 0.06 (0.02 - 0.15) | 0.15 (0.07 - 0.33) | 0.22 (0.10 - 0.49) | 0.26 (0.13 - 0.55) | 0.32 (0.15 - 0.66) | 0.36 (0.16 - 0.81) | 0.37 (0.21 - 0.66) | 0.60 (0.22 - 1.62) | **LEN+EWE** | 0.75 (0.42 - 1.37) |
| 0.04 (0.02 - 0.12) | 0.12 (0.05 - 0.25) | 0.16 (0.07 - 0.38) | 0.20 (0.09 - 0.42) | 0.24 (0.11 - 0.50) | 0.27 (0.12 - 0.61) | 0.28 (0.15 - 0.50) | 0.45 (0.16 - 1.23) | 0.75 (0.42 - 1.37) | **LEN+PEM** |

| **TIV** |  |  |  |  |  |  |  |  |  |
| --- | --- | --- | --- | --- | --- | --- | --- | --- | --- |
| 0.024 | **INF** |  |  |  |  |  |  |  |  |
| 0.008 | 0.361 | **AXI+PEM2** |  |  |  |  |  |  |  |
| 0.000 | 0.058 | 0.626 | **SOR** |  |  |  |  |  |  |
| 0.000 | 0.022 | 0.317 | 0.444 | **PAZ** |  |  |  |  |  |
| 0.000 | 0.023 | 0.213 | 0.373 | 0.713 | **AXI+AVE** |  |  |  |  |
| 0.000 | 0.000 | 0.074 | 0.139 | 0.512 | 0.950 | **SUN** |  |  |  |
| 0.000 | 0.005 | 0.049 | 0.086 | 0.188 | 0.327 | 0.253 | **CAB** |  |  |
| 0.000 | 0.000 | 0.000 | 0.000 | 0.002 | 0.013 | 0.001 | 0.311 | **LEN+EWE** |  |
| 0.000 | 0.000 | 0.000 | 0.000 | 0.000 | 0.002 | 0.000 | 0.119 | 0.352 | **LEN+PEM** |

**Supplementary Table 17. Results of a comparative analysis of all TKIs and other interventions assessed in included trials in terms of diarrhea presented as ORs with 95% CIs (upper table, the results of direct comparisons are presented above the abbreviations of TKIs) and p-values (bottom table).**

| **TEM** | . | . | . | 0.09 (0.02 - 0.30) | . | . | . | . | . | . |
| --- | --- | --- | --- | --- | --- | --- | --- | --- | --- | --- |
| 0.13 (0.03 - 0.48) | **SUN** | . | 0.69 (0.40 - 1.18) | 0.80 (0.47 - 1.35) | 0.61 (0.35 - 1.07) | 0.50 (0.30 - 0.84) | 0.55 (0.32 - 0.95) | 0.51 (0.29 - 0.89) | 0.49 (0.28 - 0.86) | 0.44 (0.19 - 0.99) |
| 0.13 (0.03 - 0.55) | 0.99 (0.47 - 2.09) | **TIV** | . | . | . | 0.61 (0.33 - 1.12) | . | . | . | . |
| 0.09 (0.02 - 0.37) | 0.69 (0.40 - 1.18) | 0.69 (0.28 - 1.74) | **AXI+PEM2** | . | . | . | . | . | . | . |
| 0.09 (0.02 - 0.30) | 0.67 (0.43 - 1.03) | 0.67 (0.31 - 1.46) | 0.97 (0.48 - 1.94) | **PAZ** | . | 1.17 (0.63 - 2.18) | . | . | . | . |
| 0.08 (0.02 - 0.33) | 0.61 (0.35 - 1.07) | 0.62 (0.24 - 1.58) | 0.90 (0.41 - 1.94) | 0.92 (0.45 - 1.87) | **LEN+PEM** | . | . | . | 0.80 (0.46 - 1.40) | . |
| 0.08 (0.02 - 0.29) | 0.60 (0.39 - 0.93) | 0.61 (0.33 - 1.12) | 0.88 (0.44 - 1.75) | 0.90 (0.56 - 1.45) | 0.98 (0.48 - 1.98) | **SOR** | . | . | . | . |
| 0.07 (0.02 - 0.30) | 0.55 (0.32 - 0.95) | 0.56 (0.22 - 1.40) | 0.80 (0.38 - 1.72) | 0.83 (0.41 - 1.66) | 0.90 (0.41 - 1.94) | 0.92 (0.46 - 1.83) | **AXI+AVE** | . | . | . |
| 0.07 (0.02 - 0.27) | 0.51 (0.29 - 0.89) | 0.51 (0.20 - 1.31) | 0.74 (0.34 - 1.61) | 0.76 (0.37 - 1.56) | 0.83 (0.37 - 1.82) | 0.84 (0.41 - 1.72) | 0.92 (0.42 - 2.00) | **CAB+NIV** | . | . |
| 0.06 (0.02 - 0.27) | 0.49 (0.28 - 0.86) | 0.50 (0.20 - 1.26) | 0.72 (0.33 - 1.56) | 0.74 (0.36 - 1.50) | 0.80 (0.46 - 1.40) | 0.82 (0.40 - 1.66) | 0.89 (0.41 - 1.94) | 0.97 (0.44 - 2.14) | **LEN+EWE** | . |
| 0.06 (0.01 - 0.27) | 0.44 (0.19 - 0.99) | 0.44 (0.14 - 1.34) | 0.63 (0.24 - 1.70) | 0.65 (0.26 - 1.67) | 0.71 (0.26 - 1.91) | 0.72 (0.28 - 1.84) | 0.79 (0.29 - 2.11) | 0.86 (0.32 - 2.33) | 0.88 (0.33 - 2.39) | **CAB** |

| **TEM** |  |  |  |  |  |  |  |  |  |  |
| --- | --- | --- | --- | --- | --- | --- | --- | --- | --- | --- |
| 0.002 | **SUN** |  |  |  |  |  |  |  |  |  |
| 0.006 | 0.979 | **TIV** |  |  |  |  |  |  |  |  |
| 0.001 | 0.171 | 0.436 | **AXI+PEM2** |  |  |  |  |  |  |  |
| 0.000 | 0.071 | 0.314 | 0.933 | **PAZ** |  |  |  |  |  |  |
| 0.001 | 0.086 | 0.316 | 0.780 | 0.824 | **LEN+PEM** |  |  |  |  |  |
| 0.000 | 0.023 | 0.108 | 0.708 | 0.674 | 0.951 | **SOR** |  |  |  |  |
| 0.000 | 0.030 | 0.214 | 0.574 | 0.595 | 0.784 | 0.808 | **AXI+AVE** |  |  |  |
| 0.000 | 0.018 | 0.162 | 0.448 | 0.457 | 0.636 | 0.642 | 0.835 | **CAB+NIV** |  |  |
| 0.000 | 0.013 | 0.142 | 0.400 | 0.404 | 0.435 | 0.580 | 0.773 | 0.939 | **LEN+EWE** |  |
| 0.000 | 0.048 | 0.148 | 0.365 | 0.373 | 0.496 | 0.498 | 0.637 | 0.762 | 0.808 | **CAB** |

**Supplementary Table 18. Results of a comparative analysis of all TKIs and other interventions assessed in included trials in terms of grade ≥3 diarrhea presented as ORs with 95% CIs (upper table, the results of direct comparisons are presented above the abbreviations of TKIs) and p-values (bottom table).**

| **TIV** | . | . | . | . | . | . | . | 0.33 (0.13 - 0.86) | . | . |
| --- | --- | --- | --- | --- | --- | --- | --- | --- | --- | --- |
| 2.02 (0.06 - 63.82) | **TEM** | . | . | 0.31 (0.01 - 7.99) | . | . | . | . | . | . |
| 0.74 (0.22 - 2.50) | 0.37 (0.01 - 9.60) | **SUN** | 1.09 (0.39 - 3.09) | 0.86 (0.56 - 1.32) | 0.62 (0.31 - 1.23) | 0.52 (0.29 - 0.95) | 0.49 (0.28 - 0.86) | 0.45 (0.16 - 1.27) | 0.43 (0.24 - 0.76) | 0.39 (0.20 - 0.78) |
| 0.81 (0.16 - 4.00) | 0.40 (0.01 - 12.33) | 1.09 (0.39 - 3.09) | **CAB** | . | . | . | . | . | . | . |
| 0.63 (0.19 - 2.13) | 0.31 (0.01 - 7.99) | 0.85 (0.57 - 1.29) | 0.78 (0.26 - 2.39) | **PAZ** | . | . | . | 0.53 (0.19 - 1.46) | . | . |
| 0.46 (0.11 - 1.86) | 0.23 (0.01 - 6.39) | 0.62 (0.31 - 1.23) | 0.57 (0.16 - 1.97) | 0.73 (0.32 - 1.62) | **CAB+NIV** | . | . | . | . | . |
| 0.39 (0.10 - 1.50) | 0.19 (0.01 - 5.29) | 0.52 (0.29 - 0.95) | 0.48 (0.14 - 1.58) | 0.61 (0.30 - 1.26) | 0.84 (0.34 - 2.09) | **LEN+PEM** | . | . | 0.82 (0.51 - 1.32) | . |
| 0.37 (0.10 - 1.39) | 0.18 (0.01 - 4.97) | 0.49 (0.28 - 0.86) | 0.45 (0.14 - 1.46) | 0.58 (0.29 - 1.16) | 0.80 (0.33 - 1.93) | 0.94 (0.42 - 2.13) | **AXI+PEM2** | . | . | . |
| 0.33 (0.13 - 0.86) | 0.17 (0.01 - 4.60) | 0.45 (0.21 - 0.96) | 0.41 (0.11 - 1.49) | 0.53 (0.25 - 1.12) | 0.73 (0.26 - 2.03) | 0.86 (0.33 - 2.26) | 0.91 (0.36 - 2.34) | **SOR** | . | . |
| 0.32 (0.08 - 1.22) | 0.16 (0.01 - 4.32) | 0.43 (0.24 - 0.76) | 0.39 (0.12 - 1.28) | 0.50 (0.25 - 1.02) | 0.69 (0.28 - 1.70) | 0.82 (0.51 - 1.32) | 0.87 (0.39 - 1.93) | 0.95 (0.37 - 2.46) | **LEN+EWE** | . |
| 0.29 (0.07 - 1.17) | 0.14 (0.01 - 4.05) | 0.39 (0.20 - 0.78) | 0.36 (0.10 - 1.24) | 0.46 (0.21 - 1.02) | 0.63 (0.24 - 1.67) | 0.75 (0.30 - 1.86) | 0.79 (0.33 - 1.92) | 0.87 (0.31 - 2.42) | 0.92 (0.37 - 2.25) | **AXI+AVE** |
|  |  |  |  |  |  |  |  |  |  |  |
| **TIV** |  |  |  |  |  |  |  |  |  |  |
| 0.691 | **TEM** |  |  |  |  |  |  |  |  |  |
| 0.630 | 0.548 | **SUN** |  |  |  |  |  |  |  |  |
| 0.798 | 0.602 | 0.865 | **CAB** |  |  |  |  |  |  |  |
| 0.461 | 0.483 | 0.456 | 0.665 | **PAZ** |  |  |  |  |  |  |
| 0.275 | 0.385 | 0.173 | 0.371 | 0.432 | **CAB+NIV** |  |  |  |  |  |
| 0.169 | 0.330 | 0.032 | 0.226 | 0.182 | 0.714 | **LEN+PEM** |  |  |  |  |
| 0.140 | 0.312 | 0.013 | 0.185 | 0.121 | 0.615 | 0.890 | **AXI+PEM2** |  |  |  |
| 0.024 | 0.289 | 0.039 | 0.177 | 0.097 | 0.543 | 0.764 | 0.851 | **SOR** |  |  |
| 0.094 | 0.274 | 0.004 | 0.121 | 0.056 | 0.419 | 0.415 | 0.727 | 0.914 | **LEN+EWE** |  |
| 0.083 | 0.255 | 0.008 | 0.106 | 0.057 | 0.357 | 0.535 | 0.610 | 0.789 | 0.849 | **AXI+AVE** |

**Supplementary Table 19. Results of a comparative analysis of all TKIs and other interventions assessed in included trials in terms of nausea presented as ORs with 95% CIs (upper table, the results of direct comparisons are presented above the abbreviations of TKIs) and p-values (bottom table).**

| **TEM** | . | . | . | . | . | . | 0.15 (0.05 - 0.49) | . | . | . |
| --- | --- | --- | --- | --- | --- | --- | --- | --- | --- | --- |
| 0.28 (0.08 - 0.95) | **SOR** | . | . | . | . | 0.59 (0.29 - 1.19) | 0.58 (0.32 - 1.04) | 0.51 (0.30 - 0.86) | . | . |
| 0.20 (0.05 - 0.82) | 0.71 (0.29 - 1.71) | **CAB** | . | . | . | . | . | 0.74 (0.34 - 1.60) | . | . |
| 0.18 (0.05 - 0.66) | 0.65 (0.35 - 1.23) | 0.92 (0.37 - 2.27) | **AXI+AVE** | . | . | . | . | 0.80 (0.50 - 1.28) | . | . |
| 0.18 (0.05 - 0.66) | 0.64 (0.33 - 1.25) | 0.90 (0.36 - 2.28) | 0.98 (0.49 - 1.96) | **CAB+NIV** | . | . | . | 0.82 (0.49 - 1.37) | . | . |
| 0.17 (0.05 - 0.64) | 0.63 (0.33 - 1.20) | 0.89 (0.36 - 2.20) | 0.96 (0.49 - 1.88) | 0.98 (0.49 - 1.98) | **AXI+PEM2** | . | . | 0.83 (0.52 - 1.35) | . | . |
| 0.16 (0.04 - 0.67) | 0.59 (0.29 - 1.19) | 0.83 (0.27 - 2.57) | 0.90 (0.35 - 2.33) | 0.92 (0.35 - 2.43) | 0.93 (0.36 - 2.43) | **TIV** | . | . | . | . |
| 0.15 (0.05 - 0.49) | 0.56 (0.36 - 0.87) | 0.79 (0.33 - 1.87) | 0.85 (0.47 - 1.57) | 0.87 (0.46 - 1.66) | 0.89 (0.48 - 1.64) | 0.95 (0.41 - 2.20) | **PAZ** | 0.96 (0.61 - 1.50) | . | . |
| 0.15 (0.04 - 0.49) | 0.52 (0.34 - 0.81) | 0.74 (0.34 - 1.60) | 0.80 (0.50 - 1.28) | 0.82 (0.49 - 1.37) | 0.83 (0.52 - 1.35) | 0.89 (0.39 - 2.05) | 0.94 (0.64 - 1.39) | **SUN** | 0.89 (0.55 - 1.46) | 0.76 (0.46 - 1.23) |
| 0.13 (0.04 - 0.48) | 0.47 (0.24 - 0.90) | 0.66 (0.27 - 1.65) | 0.72 (0.36 - 1.41) | 0.73 (0.36 - 1.49) | 0.74 (0.37 - 1.48) | 0.80 (0.30 - 2.09) | 0.84 (0.45 - 1.57) | 0.89 (0.55 - 1.46) | **LEN+PEM** | 0.85 (0.52 - 1.38) |
| 0.11 (0.03 - 0.40) | 0.40 (0.21 - 0.76) | 0.56 (0.22 - 1.39) | 0.61 (0.31 - 1.19) | 0.62 (0.31 - 1.26) | 0.63 (0.32 - 1.25) | 0.68 (0.26 - 1.77) | 0.71 (0.38 - 1.33) | 0.76 (0.46 - 1.23) | 0.85 (0.52 - 1.38) | **LEN+EWE** |

| **TEM** |  |  |  |  |  |  |  |  |  |  |
| --- | --- | --- | --- | --- | --- | --- | --- | --- | --- | --- |
| 0.041 | **SOR** |  |  |  |  |  |  |  |  |  |
| 0.026 | 0.443 | **CAB** |  |  |  |  |  |  |  |  |
| 0.010 | 0.189 | 0.861 | **AXI+AVE** |  |  |  |  |  |  |  |
| 0.010 | 0.190 | 0.832 | 0.955 | **CAB+NIV** |  |  |  |  |  |  |
| 0.008 | 0.158 | 0.800 | 0.914 | 0.961 | **AXI+PEM2** |  |  |  |  |  |
| 0.012 | 0.141 | 0.746 | 0.826 | 0.861 | 0.886 | **TIV** |  |  |  |  |
| 0.001 | 0.011 | 0.589 | 0.612 | 0.675 | 0.703 | 0.906 | **PAZ** |  |  |  |
| 0.002 | 0.003 | 0.446 | 0.359 | 0.445 | 0.457 | 0.791 | 0.755 | **SUN** |  |  |
| 0.002 | 0.023 | 0.376 | 0.337 | 0.388 | 0.399 | 0.646 | 0.584 | 0.651 | **LEN+PEM** |  |
| 0.001 | 0.005 | 0.213 | 0.148 | 0.184 | 0.186 | 0.424 | 0.283 | 0.261 | 0.500 | **LEN+EWE** |

**Supplementary Table 20. Results of a comparative analysis of all TKIs and other interventions assessed in included trials in terms of grade ≥3 nausea presented as ORs with 95% CIs (upper table, the results of direct comparisons are presented above the abbreviations of TKIs) and p-values (bottom table).**

| **TEM** | 0.13 (0.00 - 4.63) | . | . | . | . | . | . | . | . | . |
| --- | --- | --- | --- | --- | --- | --- | --- | --- | --- | --- |
| 0.13 (0.00 - 4.63) | **PAZ** | . | 0.20 (0.01 - 3.67) | . | . | . | 0.99 (0.12 - 8.43) | . | . | . |
| 0.10 (0.00 - 13.49) | 0.83 (0.03 - 21.60) | **CAB** | . | . | . | . | 0.61 (0.04 - 8.93) | . | . | . |
| 0.09 (0.00 - 5.74) | 0.70 (0.09 - 5.76) | 0.85 (0.03 - 22.17) | **SOR** | 1.01 (0.03 - 30.60) | . | . | 0.36 (0.04 - 3.08) | . | . | . |
| 0.09 (0.00 - 19.58) | 0.71 (0.01 - 38.96) | 0.85 (0.01 - 96.05) | 1.01 (0.03 - 30.60) | **TIV** | . | . | . | . | . | . |
| 0.07 (0.00 - 7.56) | 0.58 (0.03 - 10.76) | 0.70 (0.02 - 23.63) | 0.83 (0.04 - 15.39) | 0.82 (0.01 - 73.42) | **AXI+AVE** | . | 0.87 (0.09 - 8.36) | . | . | . |
| 0.06 (0.00 - 7.14) | 0.50 (0.02 - 10.63) | 0.61 (0.02 - 22.86) | 0.72 (0.03 - 15.20) | 0.72 (0.01 - 69.51) | 0.87 (0.03 - 24.15) | **AXI+PEM2** | 0.99 (0.09 - 11.18) | . | . | . |
| 0.06 (0.00 - 3.62) | 0.50 (0.08 - 3.17) | 0.61 (0.04 - 8.93) | 0.71 (0.11 - 4.53) | 0.71 (0.01 - 34.37) | 0.87 (0.09 - 8.36) | 0.99 (0.09 - 11.18) | **SUN** | 0.50 (0.02 - 11.27) | 0.23 (0.02 - 2.80) | 0.23 (0.02 - 2.78) |
| 0.03 (0.00 - 5.22) | 0.25 (0.01 - 9.34) | 0.30 (0.00 - 18.56) | 0.36 (0.01 - 13.36) | 0.35 (0.00 - 51.34) | 0.43 (0.01 - 20.38) | 0.49 (0.01 - 25.63) | 0.50 (0.02 - 11.27) | **CAB+NIV** | . | . |
| 0.01 (0.00 - 1.68) | 0.11 (0.01 - 2.57) | 0.14 (0.00 - 5.47) | 0.16 (0.01 - 3.67) | 0.16 (0.00 - 16.42) | 0.20 (0.01 - 5.80) | 0.23 (0.01 - 7.39) | 0.23 (0.02 - 2.80) | 0.46 (0.01 - 25.01) | **LEN+EWE** | 0.99 (0.11 - 8.89) |
| 0.01 (0.00 - 1.67) | 0.11 (0.00 - 2.55) | 0.14 (0.00 - 5.42) | 0.16 (0.01 - 3.64) | 0.16 (0.00 - 16.27) | 0.20 (0.01 - 5.75) | 0.22 (0.01 - 7.33) | 0.23 (0.02 - 2.78) | 0.45 (0.01 - 24.79) | 0.99 (0.11 - 8.89) | **LEN+PEM** |

| **TEM** |  |  |  |  |  |  |  |  |  |  |
| --- | --- | --- | --- | --- | --- | --- | --- | --- | --- | --- |
| 0.261 | **PAZ** |  |  |  |  |  |  |  |  |  |
| 0.363 | 0.909 | **CAB** |  |  |  |  |  |  |  |  |
| 0.255 | 0.740 | 0.921 | **SOR** |  |  |  |  |  |  |  |
| 0.380 | 0.865 | 0.948 | 0.996 | **TIV** |  |  |  |  |  |  |
| 0.269 | 0.713 | 0.842 | 0.898 | 0.931 | **AXI+AVE** |  |  |  |  |  |
| 0.253 | 0.660 | 0.790 | 0.834 | 0.886 | 0.936 | **AXI+PEM2** |  |  |  |  |
| 0.181 | 0.462 | 0.715 | 0.721 | 0.862 | 0.900 | 0.994 | **SUN** |  |  |  |
| 0.185 | 0.452 | 0.569 | 0.577 | 0.682 | 0.669 | 0.726 | 0.662 | **CAB+NIV** |  |  |
| 0.081 | 0.172 | 0.291 | 0.253 | 0.439 | 0.346 | 0.403 | 0.248 | 0.701 | **LEN+EWE** |  |
| 0.080 | 0.170 | 0.289 | 0.251 | 0.437 | 0.344 | 0.400 | 0.245 | 0.698 | 0.994 | **LEN+PEM** |

**Supplementary Table 21. Results of a comparative analysis of all TKIs and other interventions assessed in included trials in terms of vomiting presented as ORs with 95% CIs (upper table, the results of direct comparisons are presented above the abbreviations of TKIs) and p-values (bottom table).**

| **TEM** | . | . | . | . | . | . | 0.08 (0.02 - 0.37) | . | . |
| --- | --- | --- | --- | --- | --- | --- | --- | --- | --- |
| 0.74 (0.08 - 6.82) | **SOR** | . | . | . | 0.11 (0.02 - 0.52) | . | . | . | . |
| 0.10 (0.02 - 0.54) | 0.14 (0.03 - 0.69) | **AXI+PEM2** | . | . | 0.78 (0.55 - 1.12) | . | . | . | . |
| 0.10 (0.02 - 0.54) | 0.14 (0.03 - 0.68) | 0.98 (0.57 - 1.67) | **CAB+NIV** | . | 0.80 (0.54 - 1.19) | . | . | . | . |
| 0.09 (0.02 - 0.46) | 0.12 (0.03 - 0.59) | 0.86 (0.52 - 1.40) | 0.87 (0.52 - 1.47) | **AXI+AVE** | 0.91 (0.65 - 1.28) | . | . | . | . |
| 0.08 (0.02 - 0.41) | 0.11 (0.02 - 0.52) | 0.78 (0.55 - 1.12) | 0.80 (0.54 - 1.19) | 0.91 (0.65 - 1.28) | **SUN** | 0.95 (0.44 - 2.05) | 0.93 (0.72 - 1.22) | 0.71 (0.49 - 1.01) | 0.54 (0.38 - 0.76) |
| 0.08 (0.01 - 0.46) | 0.11 (0.02 - 0.59) | 0.74 (0.32 - 1.74) | 0.76 (0.32 - 1.80) | 0.87 (0.38 - 2.01) | 0.95 (0.44 - 2.05) | **CAB** | . | . | . |
| 0.08 (0.02 - 0.37) | 0.10 (0.02 - 0.49) | 0.73 (0.47 - 1.14) | 0.75 (0.46 - 1.20) | 0.85 (0.56 - 1.31) | 0.93 (0.72 - 1.22) | 0.98 (0.44 - 2.21) | **PAZ** | . | . |
| 0.06 (0.01 - 0.30) | 0.08 (0.02 - 0.38) | 0.55 (0.33 - 0.92) | 0.56 (0.33 - 0.96) | 0.65 (0.40 - 1.06) | 0.71 (0.49 - 1.01) | 0.74 (0.32 - 1.73) | 0.76 (0.48 - 1.18) | **LEN+PEM** | 0.76 (0.55 - 1.05) |
| 0.04 (0.01 - 0.23) | 0.06 (0.01 - 0.29) | 0.42 (0.25 - 0.69) | 0.43 (0.25 - 0.72) | 0.49 (0.30 - 0.79) | 0.54 (0.38 - 0.76) | 0.56 (0.24 - 1.30) | 0.57 (0.37 - 0.89) | 0.76 (0.55 - 1.05) | **LEN+EWE** |

| **TEM** |  |  |  |  |  |  |  |  |  |
| --- | --- | --- | --- | --- | --- | --- | --- | --- | --- |
| 0.790 | **SOR** |  |  |  |  |  |  |  |  |
| 0.007 | 0.015 | **AXI+PEM2** |  |  |  |  |  |  |  |
| 0.007 | 0.015 | 0.939 | **CAB+NIV** |  |  |  |  |  |  |
| 0.004 | 0.009 | 0.535 | 0.611 | **AXI+AVE** |  |  |  |  |  |
| 0.002 | 0.005 | 0.180 | 0.267 | 0.603 | **SUN** |  |  |  |  |
| 0.005 | 0.010 | 0.495 | 0.534 | 0.746 | 0.901 | **CAB** |  |  |  |
| 0.001 | 0.004 | 0.170 | 0.231 | 0.474 | 0.619 | 0.964 | **PAZ** |  |  |
| 0.001 | 0.002 | 0.022 | 0.036 | 0.081 | 0.056 | 0.488 | 0.217 | **LEN+PEM** |  |
| 0.000 | 0.000 | 0.001 | 0.002 | 0.004 | 0.000 | 0.179 | 0.012 | 0.096 | **LEN+EWE** |

**Supplementary Table 22. Results of a comparative analysis of all TKIs and other interventions assessed in included trials in terms of grade ≥3 vomiting presented as ORs with 95% CIs (upper table, the results of direct comparisons are presented above the abbreviations of TKIs) and p-values (bottom table).**

| **TEM** | . | . | . | . | 0.18 (0.01 - 3.96) | . | . | . | . |
| --- | --- | --- | --- | --- | --- | --- | --- | --- | --- |
| 0.50 (0.01 - 23.71) | **AXI+PEM2** | . | . | . | . | 0.25 (0.03 - 2.21) | . | . | . |
| 0.42 (0.00 - 38.15) | 0.83 (0.02 - 40.87) | **SOR** | . | . | . | 0.30 (0.01 - 7.43) | . | . | . |
| 0.27 (0.01 - 14.66) | 0.54 (0.02 - 14.22) | 0.65 (0.01 - 36.70) | **CAB** | . | . | 0.45 (0.04 - 5.12) | . | . | . |
| 0.21 (0.01 - 6.45) | 0.43 (0.03 - 5.32) | 0.52 (0.02 - 16.27) | 0.79 (0.05 - 12.01) | **AXI+AVE** | . | 0.57 (0.17 - 1.98) | . | . | . |
| 0.18 (0.01 - 3.96) | 0.37 (0.04 - 3.75) | 0.44 (0.02 - 12.09) | 0.67 (0.05 - 8.59) | 0.85 (0.20 - 3.67) | **PAZ** | 0.67 (0.31 - 1.46) | . | . | . |
| 0.12 (0.01 - 2.94) | 0.25 (0.03 - 2.21) | 0.30 (0.01 - 7.43) | 0.45 (0.04 - 5.12) | 0.57 (0.17 - 1.98) | 0.67 (0.31 - 1.46) | **SUN** | 0.51 (0.17 - 1.52) | 0.42 (0.15 - 1.21) | 0.16 (0.02 - 1.37) |
| 0.06 (0.00 - 1.81) | 0.13 (0.01 - 1.47) | 0.15 (0.01 - 4.57) | 0.23 (0.02 - 3.33) | 0.30 (0.06 - 1.53) | 0.35 (0.09 - 1.32) | 0.51 (0.17 - 1.52) | **LEN+EWE** | 0.82 (0.35 - 1.93) | . |
| 0.05 (0.00 - 1.47) | 0.10 (0.01 - 1.19) | 0.13 (0.00 - 3.72) | 0.19 (0.01 - 2.70) | 0.24 (0.05 - 1.23) | 0.28 (0.08 - 1.06) | 0.42 (0.15 - 1.21) | 0.82 (0.35 - 1.93) | **LEN+PEM** | . |
| 0.02 (0.00 - 0.92) | 0.04 (0.00 - 0.86) | 0.05 (0.00 - 2.30) | 0.07 (0.00 - 1.87) | 0.09 (0.01 - 1.10) | 0.11 (0.01 - 1.06) | 0.16 (0.02 - 1.37) | 0.32 (0.03 - 3.45) | 0.39 (0.04 - 4.15) | **CAB+NIV** |

| **TEM** |  |  |  |  |  |  |  |  |  |
| --- | --- | --- | --- | --- | --- | --- | --- | --- | --- |
| 0.726 | **AXI+PEM2** |  |  |  |  |  |  |  |  |
| 0.704 | 0.925 | **SOR** |  |  |  |  |  |  |  |
| 0.522 | 0.713 | 0.835 | **CAB** |  |  |  |  |  |  |
| 0.376 | 0.510 | 0.707 | 0.866 | **AXI+AVE** |  |  |  |  |  |
| 0.279 | 0.396 | 0.627 | 0.762 | 0.830 | **PAZ** |  |  |  |  |
| 0.196 | 0.210 | 0.460 | 0.523 | 0.379 | 0.319 | **SUN** |  |  |  |
| 0.107 | 0.098 | 0.278 | 0.283 | 0.146 | 0.120 | 0.230 | **LEN+EWE** |  |  |
| 0.083 | 0.069 | 0.230 | 0.221 | 0.088 | 0.060 | 0.110 | 0.651 | **LEN+PEM** |  |
| 0.045 | 0.039 | 0.125 | 0.114 | 0.059 | 0.056 | 0.095 | 0.347 | 0.434 | **CAB+NIV** |

**Supplementary Table 23. Results of a comparative analysis of all TKIs and other interventions assessed in included trials in terms of fatigue presented as ORs with 95% CIs (upper table, the results of direct comparisons are presented above the abbreviations of TKIs) and p-values (bottom table).**

| **TEM** | . | 0.46 (0.17 - 1.21) | . | . | . | . | . | . | . | . |
| --- | --- | --- | --- | --- | --- | --- | --- | --- | --- | --- |
| 0.56 (0.20 - 1.55) | **SOR** | 0.73 (0.48 - 1.11) | 0.79 (0.50 - 1.25) | . | . | 0.67 (0.46 - 0.97) | . | . | . | . |
| 0.46 (0.17 - 1.21) | 0.82 (0.60 - 1.11) | **PAZ** | . | . | . | 0.71 (0.56 - 0.90) | . | . | . | . |
| 0.44 (0.15 - 1.35) | 0.79 (0.50 - 1.25) | 0.97 (0.56 - 1.67) | **TIV** | . | . | . | . | . | . | . |
| 0.41 (0.12 - 1.35) | 0.72 (0.35 - 1.52) | 0.88 (0.43 - 1.80) | 0.91 (0.38 - 2.17) | **CAB** | . | 0.84 (0.43 - 1.65) | . | . | . | . |
| 0.38 (0.13 - 1.08) | 0.68 (0.44 - 1.06) | 0.83 (0.56 - 1.23) | 0.85 (0.45 - 1.61) | 0.94 (0.44 - 1.99) | **CAB+NIV** | 0.89 (0.64 - 1.24) | . | . | . | . |
| 0.34 (0.13 - 0.92) | 0.61 (0.45 - 0.81) | 0.74 (0.59 - 0.92) | 0.76 (0.44 - 1.31) | 0.84 (0.43 - 1.65) | 0.89 (0.64 - 1.24) | **SUN** | 0.98 (0.74 - 1.29) | 0.94 (0.72 - 1.24) | 0.87 (0.64 - 1.18) | 0.80 (0.59 - 1.09) |
| 0.33 (0.12 - 0.93) | 0.59 (0.39 - 0.89) | 0.72 (0.51 - 1.03) | 0.75 (0.41 - 1.37) | 0.82 (0.39 - 1.70) | 0.87 (0.57 - 1.34) | 0.98 (0.74 - 1.29) | **AXI+PEM2** | . | . | . |
| 0.32 (0.11 - 0.90) | 0.57 (0.38 - 0.85) | 0.70 (0.49 - 0.99) | 0.72 (0.39 - 1.32) | 0.79 (0.38 - 1.64) | 0.84 (0.55 - 1.29) | 0.94 (0.72 - 1.24) | 0.97 (0.66 - 1.42) | **AXI+AVE** | . | . |
| 0.30 (0.10 - 0.84) | 0.53 (0.34 - 0.81) | 0.64 (0.44 - 0.94) | 0.66 (0.36 - 1.24) | 0.73 (0.35 - 1.53) | 0.78 (0.50 - 1.22) | 0.87 (0.64 - 1.18) | 0.89 (0.59 - 1.35) | 0.92 (0.61 - 1.39) | **LEN+PEM** | 0.92 (0.68 - 1.25) |
| 0.27 (0.10 - 0.77) | 0.49 (0.32 - 0.75) | 0.59 (0.41 - 0.87) | 0.61 (0.33 - 1.14) | 0.67 (0.32 - 1.42) | 0.72 (0.46 - 1.12) | 0.80 (0.59 - 1.09) | 0.82 (0.55 - 1.24) | 0.85 (0.57 - 1.28) | 0.92 (0.68 - 1.25) | **LEN+EWE** |

| **TEM** |  |  |  |  |  |  |  |  |  |  |
| --- | --- | --- | --- | --- | --- | --- | --- | --- | --- | --- |
| 0.263 | **SOR** |  |  |  |  |  |  |  |  |  |
| 0.115 | 0.202 | **PAZ** |  |  |  |  |  |  |  |  |
| 0.153 | 0.318 | 0.906 | **TIV** |  |  |  |  |  |  |  |
| 0.141 | 0.391 | 0.730 | 0.834 | **CAB** |  |  |  |  |  |  |
| 0.070 | 0.085 | 0.348 | 0.628 | 0.867 | **CAB+NIV** |  |  |  |  |  |
| 0.033 | 0.001 | 0.007 | 0.331 | 0.610 | 0.503 | **SUN** |  |  |  |  |
| 0.036 | 0.011 | 0.070 | 0.344 | 0.590 | 0.532 | 0.862 | **AXI+PEM2** |  |  |  |
| 0.030 | 0.006 | 0.044 | 0.291 | 0.530 | 0.434 | 0.677 | 0.868 | **AXI+AVE** |  |  |
| 0.021 | 0.003 | 0.022 | 0.199 | 0.406 | 0.273 | 0.374 | 0.586 | 0.694 | **LEN+PEM** |  |
| 0.014 | 0.001 | 0.007 | 0.125 | 0.298 | 0.148 | 0.160 | 0.356 | 0.438 | 0.605 | **LEN+EWE** |

**Supplementary Table 24. Results of a comparative analysis of all TKIs and other interventions assessed in included trials in terms of grade ≥3 fatigue presented as ORs with 95% CIs (upper table, the results of direct comparisons are presented above the abbreviations of TKIs) and p-values (bottom table).**

| **CAB** | . | . | . | . | . | . | . | . | 0.34 (0.09 - 1.36) | . |
| --- | --- | --- | --- | --- | --- | --- | --- | --- | --- | --- |
| 0.82 (0.15 - 4.39) | **SOR** | . | . | 1.00 (0.16 - 6.15) | 0.64 (0.19 - 2.10) | . | . | . | 0.38 (0.13 - 1.12) | . |
| 0.84 (0.15 - 4.84) | 1.02 (0.24 - 4.33) | **AXI+PEM2** | . | . | . | . | . | . | 0.41 (0.14 - 1.20) | . |
| 0.79 (0.09 - 7.03) | 0.97 (0.15 - 6.13) | 0.95 (0.13 - 7.04) | **TEM** | 0.80 (0.18 - 3.47) | . | . | . | . | . | . |
| 0.63 (0.13 - 3.17) | 0.77 (0.25 - 2.36) | 0.76 (0.19 - 2.96) | 0.80 (0.18 - 3.47) | **PAZ** | . | . | . | . | 0.58 (0.23 - 1.42) | . |
| 0.52 (0.07 - 4.08) | 0.64 (0.19 - 2.10) | 0.62 (0.10 - 4.04) | 0.66 (0.07 - 5.90) | 0.82 (0.16 - 4.21) | **TIV** | . | . | . | . | . |
| 0.47 (0.08 - 2.85) | 0.58 (0.13 - 2.58) | 0.56 (0.12 - 2.74) | 0.60 (0.08 - 4.60) | 0.75 (0.18 - 3.09) | 0.91 (0.13 - 6.16) | **CAB+NIV** | . | . | 0.72 (0.23 - 2.29) | . |
| 0.36 (0.06 - 2.08) | 0.43 (0.10 - 1.87) | 0.42 (0.09 - 1.99) | 0.45 (0.06 - 3.37) | 0.56 (0.14 - 2.24) | 0.68 (0.10 - 4.51) | 0.75 (0.15 - 3.71) | **LEN+PEM** | . | 0.96 (0.32 - 2.93) | 0.54 (0.19 - 1.56) |
| 0.36 (0.06 - 2.11) | 0.44 (0.10 - 1.89) | 0.43 (0.09 - 2.02) | 0.46 (0.06 - 3.42) | 0.57 (0.14 - 2.27) | 0.69 (0.11 - 4.57) | 0.76 (0.16 - 3.76) | 1.02 (0.21 - 4.86) | **AXI+AVE** | 0.95 (0.32 - 2.84) | . |
| 0.34 (0.09 - 1.36) | 0.42 (0.16 - 1.08) | 0.41 (0.14 - 1.20) | 0.43 (0.08 - 2.33) | 0.54 (0.24 - 1.24) | 0.66 (0.14 - 3.04) | 0.72 (0.23 - 2.29) | 0.96 (0.32 - 2.93) | 0.95 (0.32 - 2.84) | **SUN** | 0.56 (0.19 - 1.61) |
| 0.19 (0.03 - 1.09) | 0.23 (0.06 - 0.97) | 0.23 (0.05 - 1.04) | 0.24 (0.03 - 1.77) | 0.30 (0.08 - 1.16) | 0.37 (0.06 - 2.37) | 0.41 (0.09 - 1.94) | 0.54 (0.19 - 1.56) | 0.53 (0.12 - 2.44) | 0.56 (0.19 - 1.61) | **LEN+EWE** |

| **CAB** |  |  |  |  |  |  |  |  |  |  |
| --- | --- | --- | --- | --- | --- | --- | --- | --- | --- | --- |
| 0.817 | **SOR** |  |  |  |  |  |  |  |  |  |
| 0.845 | 0.975 | **AXI+PEM2** |  |  |  |  |  |  |  |  |
| 0.836 | 0.973 | 0.957 | **TEM** |  |  |  |  |  |  |  |
| 0.579 | 0.651 | 0.687 | 0.763 | **PAZ** |  |  |  |  |  |  |
| 0.535 | 0.457 | 0.618 | 0.706 | 0.813 | **TIV** |  |  |  |  |  |
| 0.414 | 0.471 | 0.477 | 0.619 | 0.686 | 0.922 | **CAB+NIV** |  |  |  |  |
| 0.252 | 0.262 | 0.277 | 0.435 | 0.413 | 0.691 | 0.725 | **LEN+PEM** |  |  |  |
| 0.259 | 0.271 | 0.285 | 0.444 | 0.425 | 0.705 | 0.741 | 0.981 | **AXI+AVE** |  |  |
| 0.128 | 0.073 | 0.105 | 0.329 | 0.147 | 0.591 | 0.582 | 0.949 | 0.922 | **SUN** |  |
| 0.063 | 0.046 | 0.056 | 0.162 | 0.082 | 0.293 | 0.258 | 0.254 | 0.416 | 0.283 | **LEN+EWE** |

**Supplementary Table 25. Results of a comparative analysis of all TKIs and other interventions assessed in included trials in terms of hypertension presented as ORs with 95% CIs (upper table; the results of direct comparisons are presented above the abbreviations of TKIs) and p-values (bottom table).**

| **TEM** | . | . | . | . | . | . | 0.11 (0.03 - 0.41) | . | . | . |
| --- | --- | --- | --- | --- | --- | --- | --- | --- | --- | --- |
| 0.21 (0.06 - 0.80) | **SOR** | . | . | 0.84 (0.55 - 1.30) | 0.65 (0.42 - 1.02) | . | 0.45 (0.27 - 0.75) | . | . | . |
| 0.18 (0.04 - 0.69) | 0.83 (0.48 - 1.43) | **CAB+NIV** | . | 0.90 (0.59 - 1.36) | . | . | . | . | . | . |
| 0.16 (0.04 - 0.64) | 0.77 (0.46 - 1.29) | 0.93 (0.53 - 1.64) | **AXI+PEM2** | 0.96 (0.66 - 1.41) | . | . | . | . | . | . |
| 0.16 (0.04 - 0.58) | 0.74 (0.52 - 1.06) | 0.90 (0.59 - 1.36) | 0.96 (0.66 - 1.41) | **SUN** | . | 0.84 (0.56 - 1.26) | 0.79 (0.55 - 1.13) | 0.57 (0.39 - 0.84) | 0.57 (0.38 - 0.85) | 0.40 (0.20 - 0.82) |
| 0.14 (0.03 - 0.56) | 0.65 (0.42 - 1.02) | 0.79 (0.39 - 1.60) | 0.85 (0.43 - 1.68) | 0.88 (0.50 - 1.55) | **TIV** | . | . | . | . | . |
| 0.13 (0.03 - 0.52) | 0.63 (0.37 - 1.07) | 0.76 (0.42 - 1.35) | 0.81 (0.47 - 1.42) | 0.84 (0.56 - 1.26) | 0.96 (0.48 - 1.93) | **LEN+EWE** | . | . | 0.68 (0.45 - 1.01) | . |
| 0.11 (0.03 - 0.41) | 0.54 (0.37 - 0.79) | 0.65 (0.39 - 1.10) | 0.70 (0.43 - 1.15) | 0.73 (0.53 - 1.00) | 0.83 (0.46 - 1.48) | 0.86 (0.52 - 1.43) | **PAZ** | . | . | . |
| 0.09 (0.02 - 0.35) | 0.43 (0.25 - 0.72) | 0.51 (0.29 - 0.90) | 0.55 (0.32 - 0.95) | 0.57 (0.39 - 0.84) | 0.65 (0.33 - 1.29) | 0.68 (0.39 - 1.18) | 0.79 (0.48 - 1.29) | **AXI+AVE** | . | . |
| 0.09 (0.02 - 0.35) | 0.42 (0.25 - 0.72) | 0.51 (0.29 - 0.91) | 0.55 (0.32 - 0.96) | 0.57 (0.38 - 0.85) | 0.65 (0.32 - 1.30) | 0.68 (0.45 - 1.01) | 0.79 (0.47 - 1.31) | 1.00 (0.57 - 1.73) | **LEN+PEM** | . |
| 0.06 (0.01 - 0.28) | 0.30 (0.13 - 0.66) | 0.36 (0.16 - 0.82) | 0.39 (0.17 - 0.87) | 0.40 (0.20 - 0.82) | 0.46 (0.18 - 1.13) | 0.47 (0.21 - 1.07) | 0.55 (0.25 - 1.20) | 0.70 (0.31 - 1.57) | 0.70 (0.31 - 1.59) | **CAB** |

| **TEM** |  |  |  |  |  |  |  |  |  |  |
| --- | --- | --- | --- | --- | --- | --- | --- | --- | --- | --- |
| 0.022 | **SOR** |  |  |  |  |  |  |  |  |  |
| 0.013 | 0.500 | **CAB+NIV** |  |  |  |  |  |  |  |  |
| 0.009 | 0.324 | 0.801 | **AXI+PEM2** |  |  |  |  |  |  |  |
| 0.006 | 0.100 | 0.611 | 0.853 | **SUN** |  |  |  |  |  |  |
| 0.006 | 0.059 | 0.507 | 0.634 | 0.653 | **TIV** |  |  |  |  |  |
| 0.004 | 0.088 | 0.348 | 0.466 | 0.408 | 0.912 | **LEN+EWE** |  |  |  |  |
| 0.001 | 0.001 | 0.110 | 0.158 | 0.047 | 0.524 | 0.564 | **PAZ** |  |  |  |
| 0.001 | 0.001 | 0.021 | 0.030 | 0.004 | 0.221 | 0.169 | 0.346 | **AXI+AVE** |  |  |
| 0.001 | 0.002 | 0.024 | 0.034 | 0.006 | 0.225 | 0.054 | 0.355 | 0.989 | **LEN+PEM** |  |
| 0.000 | 0.003 | 0.015 | 0.021 | 0.012 | 0.091 | 0.074 | 0.134 | 0.384 | 0.396 | **CAB** |

**Supplementary Table 26. Results of a comparative analysis of all TKIs and other interventions assessed in included trials in terms of grade ≥3 hypertension presented as ORs with 95% CIs (upper table) and p-values (bottom table).**

| **TEM** | . | . | . | . | . | . | 0.15 (0.03 - 0.82) | . | . | . |
| --- | --- | --- | --- | --- | --- | --- | --- | --- | --- | --- |
| 0.27 (0.04 - 1.75) | **SOR** | . | 0.87 (0.42 - 1.78) | 0.59 (0.27 - 1.26) | . | . | 0.37 (0.15 - 0.88) | . | . | . |
| 0.20 (0.03 - 1.45) | 0.72 (0.27 - 1.94) | **CAB+NIV** | 0.95 (0.43 - 2.08) | . | . | . | . | . | . | . |
| 0.19 (0.03 - 1.16) | 0.68 (0.37 - 1.25) | 0.95 (0.43 - 2.08) | **SUN** | . | 0.84 (0.41 - 1.72) | 0.80 (0.38 - 1.66) | 1.00 (0.49 - 2.05) | 0.67 (0.25 - 1.80) | 0.61 (0.29 - 1.27) | 0.60 (0.29 - 1.23) |
| 0.16 (0.02 - 1.20) | 0.59 (0.27 - 1.26) | 0.82 (0.23 - 2.88) | 0.87 (0.33 - 2.30) | **TIV** | . | . | . | . | . | . |
| 0.16 (0.02 - 1.12) | 0.57 (0.22 - 1.46) | 0.79 (0.27 - 2.31) | 0.84 (0.41 - 1.72) | 0.97 (0.29 - 3.25) | **AXI+PEM2** | . | . | . | . | . |
| 0.15 (0.02 - 1.07) | 0.54 (0.21 - 1.40) | 0.75 (0.26 - 2.21) | 0.80 (0.38 - 1.66) | 0.92 (0.27 - 3.12) | 0.95 (0.34 - 2.65) | **LEN+EWE** | . | . | 0.76 (0.37 - 1.57) | . |
| 0.15 (0.03 - 0.82) | 0.53 (0.27 - 1.03) | 0.74 (0.27 - 2.00) | 0.78 (0.43 - 1.43) | 0.90 (0.33 - 2.48) | 0.93 (0.36 - 2.38) | 0.98 (0.38 - 2.54) | **PAZ** | . | . | . |
| 0.12 (0.02 - 1.00) | 0.45 (0.14 - 1.45) | 0.63 (0.18 - 2.24) | 0.67 (0.25 - 1.80) | 0.77 (0.19 - 3.09) | 0.80 (0.24 - 2.70) | 0.84 (0.25 - 2.88) | 0.86 (0.27 - 2.73) | **CAB** | . | . |
| 0.11 (0.02 - 0.82) | 0.41 (0.16 - 1.07) | 0.58 (0.20 - 1.69) | 0.61 (0.29 - 1.27) | 0.70 (0.21 - 2.38) | 0.73 (0.26 - 2.02) | 0.76 (0.37 - 1.57) | 0.78 (0.30 - 2.02) | 0.91 (0.27 - 3.10) | **LEN+PEM** | . |
| 0.11 (0.02 - 0.80) | 0.41 (0.16 - 1.04) | 0.57 (0.20 - 1.64) | 0.60 (0.29 - 1.23) | 0.69 (0.21 - 2.32) | 0.71 (0.26 - 1.97) | 0.75 (0.27 - 2.10) | 0.77 (0.30 - 1.96) | 0.90 (0.26 - 3.03) | 0.98 (0.35 - 2.74) | **AXI+AVE** |

| **TEM** |  |  |  |  |  |  |  |  |  |  |
| --- | --- | --- | --- | --- | --- | --- | --- | --- | --- | --- |
| 0.171 | **SOR** |  |  |  |  |  |  |  |  |  |
| 0.110 | 0.513 | **CAB+NIV** |  |  |  |  |  |  |  |  |
| 0.072 | 0.211 | 0.889 | **SUN** |  |  |  |  |  |  |  |
| 0.075 | 0.173 | 0.757 | 0.776 | **TIV** |  |  |  |  |  |  |
| 0.065 | 0.242 | 0.673 | 0.635 | 0.959 | **AXI+PEM2** |  |  |  |  |  |
| 0.058 | 0.207 | 0.607 | 0.546 | 0.891 | 0.919 | **LEN+EWE** |  |  |  |  |
| 0.029 | 0.062 | 0.551 | 0.426 | 0.839 | 0.879 | 0.967 | **PAZ** |  |  |  |
| 0.050 | 0.182 | 0.478 | 0.426 | 0.714 | 0.715 | 0.782 | 0.795 | **CAB** |  |  |
| 0.031 | 0.069 | 0.315 | 0.184 | 0.570 | 0.539 | 0.467 | 0.609 | 0.880 | **LEN+PEM** |  |
| 0.029 | 0.061 | 0.296 | 0.162 | 0.549 | 0.514 | 0.587 | 0.581 | 0.859 | 0.975 | **AXI+AVE** |

**Supplementary Table 27. Results of a comparative analysis of all TKIs and other interventions assessed in included trials in terms of dysphonia presented as ORs with 95% CIs (upper table) and p-values (bottom table).**

| **SUN** | 0.17 (0.09 - 0.33) | 0.14 (0.08 - 0.25) | 0.10 (0.02 - 0.46) | 0.10 (0.06 - 0.18) | 0.10 (0.06 - 0.18) | 0.07 (0.04 - 0.13) |
| --- | --- | --- | --- | --- | --- | --- |
| 0.17 (0.09 - 0.33) | **CAB+NIV** | . | . | . | . | . |
| 0.14 (0.08 - 0.25) | 0.81 (0.33 - 1.97) | **LEN+EWE** | . | . | 0.73 (0.52 - 1.02) | . |
| 0.10 (0.02 - 0.46) | 0.60 (0.12 - 3.10) | 0.74 (0.15 - 3.72) | **CAB** | . | . | . |
| 0.10 (0.06 - 0.18) | 0.58 (0.24 - 1.41) | 0.72 (0.32 - 1.64) | 0.98 (0.19 - 4.89) | **AXI+PEM2** | . | . |
| 0.10 (0.06 - 0.18) | 0.59 (0.24 - 1.43) | 0.73 (0.52 - 1.02) | 0.99 (0.20 - 4.95) | 1.01 (0.45 - 2.29) | **LEN+PEM** | . |
| 0.07 (0.04 - 0.13) | 0.43 (0.18 - 1.05) | 0.54 (0.24 - 1.22) | 0.73 (0.15 - 3.64) | 0.75 (0.33 - 1.68) | 0.74 (0.33 - 1.67) | **AXI+AVE** |

| **SUN** |  |  |  |  |  |  |
| --- | --- | --- | --- | --- | --- | --- |
| 0.000 | **CAB+NIV** |  |  |  |  |  |
| 0.000 | 0.638 | **LEN+EWE** |  |  |  |  |
| 0.003 | 0.540 | 0.715 | **CAB** |  |  |  |
| 0.000 | 0.230 | 0.437 | 0.976 | **AXI+PEM2** |  |  |
| 0.000 | 0.241 | 0.064 | 0.986 | 0.981 | **LEN+PEM** |  |
| 0.000 | 0.063 | 0.138 | 0.698 | 0.477 | 0.465 | **AXI+AVE** |

## **Heterogeneity assessments**

**Supplementary Table 28. Heterogeneity assessment.**

| **Endpoint** | **Tau** | **I^2^** | **Q value - total** | **p value (Q test) - total** | **Q value - within designs** | **p value**  **(Q test) – within designs** | **Q value – between designs** | **p value**  **(Q test) - between designs** |
| --- | --- | --- | --- | --- | --- | --- | --- | --- |
| 1. Adverse events | 0.0 | 0% | 0.09 | 0.7642 | 0.00 | - | 0.09 | 0.7642 |
| 2. Grade ≥3 adverse events | 0.0 | 0% | 1.92 | 0.3825 | 1.92 | 0.1659 | 0.00 | 0.9624 |
| 3. Discontinuation due to adverse events | **0.4826** | **75.1%** | 12.04 | **0.0072** | 0.53 | 0.4662 | **11.51** | **0.0032** |
| 4. Dose changes due to adverse events | 0.2478 | 57.9% | 4.75 | 0.0930 | 0.00 | - | 4.75 | 0.0930 |
| 5. Fatigue | 0.0 | 0% | 0.85 | 0.6549 | 0.19 | 0.6662 | 0.66 | 0.4164 |
| 6. Diarrhea | 0.2374 | 51.3% | 4.11 | 0.1284 | 0.48 | 0.4883 | 3.62 | 0.0569 |
| 7. Nausea | 0.1930 | 35.7% | 3.11 | 0.2111 | 3.11 | 0.0780 | 0.01 | 0.9421 |
| 8. Vomiting | NA | NA | - | - | - | - | - | - |
| 9. Hypertension | 0.1362 | 25.1% | 2.67 | 0.2631 | 1.01 | 0.3137 | 1.66 | 0.1982 |
| 10. Dysphonia | NA | NA | - | - | - | - | - | - |
| 11. Grade ≥3 fatigue | 0.4256 | 29.1% | 2.82 | 0.2438 | 2.74 | 0.0979 | 0.08 | 0.7724 |
| 12. Grade ≥3 diarrhea | 0.0 | 0% | 0.94 | 0.6248 | 0.94 | 0.3321 | 0.00 | 0.9902 |
| 13. Grade ≥3 nausea | 1.0123 | 49.8% | 3.98 | 0.1364 | 0.89 | 0.3445 | 3.09 | 0.078 |
| 14. Grade ≥3 vomiting | NA | NA | - | - | - | - | - | - |
| 15. Grade ≥3 hypertension | 0.3248 | 48.9% | 3.92 | 0.1411 | 0.48 | 0.4858 | 3.43 | 0.0640 |

NA – not assessable.
